# Modular-designed engineered bacteria for precision tumor immunotherapy via spatiotemporal manipulation by magnetic field

**DOI:** 10.1038/s41467-023-37225-1

**Published:** 2023-03-23

**Authors:** Xiaotu Ma, Xiaolong Liang, Yao Li, Qingqing Feng, Keman Cheng, Nana Ma, Fei Zhu, Xinjing Guo, Yale Yue, Guangna Liu, Tianjiao Zhang, Jie Liang, Lei Ren, Xiao Zhao, Guangjun Nie

**Affiliations:** 1 CAS Key Laboratory for Biomedical Effects of Nanomaterials and Nanosafety & CAS Center for Excellence in Nanoscience, National Center for Nanoscience and Technology, Beijing, 100190 China; 2 Department of Ultrasound, Peking University Third Hospital, Beijing, 100191 China; 3 IGDB-NCNST Joint Research Center, Institute of Genetics and Developmental Biology, Chinese Academy of Sciences, Beijing, 100101 China; 4 The Higher Educational Key Laboratory of Biomedical Engineering of Fujian Province, Research Center of Biomedical Engineering of Xiamen, Department of Biomaterials, College of Materials, Xiamen University, Xiamen, Fujian 361005 China; 5 Center of Materials Science and Optoelectronics Engineering, University of Chinese Academy of Sciences, Beijing, 100049 China; 6 The GBA National Institute for Nanotechnology Innovation, Guangdong, 510700 China

**Keywords:** Cancer immunotherapy, Bacterial synthetic biology, Nanotechnology in cancer

## Abstract

Micro-nano biorobots based on bacteria have demonstrated great potential for tumor diagnosis and treatment. The bacterial gene expression and drug release should be spatiotemporally controlled to avoid drug release in healthy tissues and undesired toxicity. Herein, we describe an alternating magnetic field-manipulated tumor-homing bacteria developed by genetically modifying engineered *Escherichia coli* with Fe_3_O_4_@lipid nanocomposites. After accumulating in orthotopic colon tumors in female mice, the paramagnetic Fe_3_O_4_ nanoparticles enable the engineered bacteria to receive and convert magnetic signals into heat, thereby initiating expression of lysis proteins under the control of a heat-sensitive promoter. The engineered bacteria then lyse, releasing its anti-CD47 nanobody cargo, that is pre-expressed and within the bacteria. The robust immunogenicity of bacterial lysate cooperates with anti-CD47 nanobody to activate both innate and adaptive immune responses, generating robust antitumor effects against not only orthotopic colon tumors but also distal tumors in female mice. The magnetically engineered bacteria also enable the constant magnetic field-controlled motion for enhanced tumor targeting and increased therapeutic efficacy. Thus, the gene expression and drug release behavior of tumor-homing bacteria can be spatiotemporally manipulated in vivo by a magnetic field, achieving tumor-specific CD47 blockage and precision tumor immunotherapy.

## Abstract (web summary)

Several strategies have been employed to enhance the tumor-targeting and anti-cancer properties of engineered bacteria. Here the authors describe the design of alternating magnetic field-manipulated bacteria engineered to release an anti-CD47 nanobody, promoting anti-tumor immune response in preclinical cancer models.

## Introduction

Bacteria have been exploited for tumor treatment since as early as the nineteenth century^1^. An in-depth understanding of the tumor microenvironment reveals that the success of bacterial therapy is owed to the natural tumor-targeting ability of many genera of bacteria, including *Escherichia*, *Salmonella*, *Listeria*, *Clostridium* and *Bifidobacterium*, all of which preferentially accumulate in tumor tissues^1^. Obligate anaerobic bacteria (e.g., *Clostridium* spp.) can specifically colonize the hypoxic areas of solid tumors^2^. Facultative anaerobes (e.g., *Escherichia* spp.) have chemotactic receptors and flagella for sensing and propulsion, respectively^1,3,4^. The chemotactic receptors direct the bacteria towards the molecular signals generated in the tumor microenvironment^1,3^, while the flagella enable the bacteria to penetrate various physiological barriers and “swim” into the deep tumor tissue^1,2,4^. The immunosuppressive tumor microenvironment prevents those bacteria from being eliminated by the immune system, facilitating the preferential growth of bacteria in tumors^5^. The first generation of bacterial therapy used natural, live bacteria, inactivated or deactivated, to treat tumors^1,2^. One successful example is Bacillus Calmette-Guérin (BCG), composed of live *Mycobacterium tuberculosis*, for the treatment of bladder cancer in the clinic. The mechanisms of first-generation bacterial therapy to destroy solid tumors depend on the intrinsic antitumor activities of bacteria, including the direct lysis of tumor cells, competition for nutrients, and/or sensitization of antitumor immune responses^6^. With the development of molecular cloning technology, the second generation of bacterial therapy employed genetically engineered bacteria to possess enhanced and integrated antitumor functions, as well as improved biosafety^7^. The knocking out of certain genes of virulence factors generates attenuated bacteria with reduced toxicity (e.g., the deletion of the *msbB* gene in *Salmonella* spp. leads to the loss of lipopolysaccharide (LPS) and toxicity reduced by 10,000-fold)^7^. Engineering bacteria to express cytotoxic agents or tumor-targeting molecules has enhanced anticancer efficiency or improved tumor-targeting. For example, the surface display of antibody fragments against the colorectal cancer-associated carcinoembryonic antigen (CEA) made engineered *Salmonella* spp. more effective in treating CEA-expressed tumors^6^. Additionally, bacteria engineered with inducible promoters can achieve temporally or spatially controlled gene expression. For example, ionic irradiation at 2 Gy enabled the activation of the irradiation-inducible promoter, RecA, on a plasmid transfected into *Clostridium* spp. to achieve irradiation-controlled gene expression^1^.

Recently, with the rapid development of nanotechnology, a third generation of customized nanomaterial-assisted bacteria, integrating multiple functions and exhibiting biological robots-like behavior, have emerged^8^. Nanomaterial modification can significantly expand the functions of engineered bacteria to include activity that cannot be achieved via traditional genetic engineering^8^. The conjugation of aptamers to bacterial surface achieves targeted intratumoral localization and enhanced tumor biotherapy^9^. Tumor-targeting *Escherichia* spp. can be modified with drug-loaded microparticles, realizing the tumor-specific delivery of various anticancer drugs, especially small molecular drugs (e.g., chemotherapeutic doxorubicin)^10^ and complex protein drugs (such as immunotherapeutic anti-programmed death ligand 1 [PD-L1] antibody)^11,12^ that can hardly be synthesized through genetic engineering of the bacteria. Tumor-targeting *Salmonella* spp., modified by nano-photosensitizers (indocyanine green-loaded nanoparticles, polydopamine, and melanin), can realize tumor-specific photothermal/photodynamic therapy^11–14^. It has also been reported that modification of *Spirulina* microalgae with nano-contrast agents (superparamagnetic magnetite suspensions) enabled the tracing of bacterial behavior in vivo by magnetic resonance imaging^15,16^. Coating the gut microbe, *Escherichia coli (E. coli) Nissle* 1917, with a self-assembled and biocompatible lipid membrane can improve bacterial survival in the intestine and increase the oral bioavailability of transplanted microbiota^17^. Coating *Escherichia* or *Salmonella* spp. with erythrocyte membranes generated stealth bacteria could enter the blood without inducing a marked inflammatory response and evade elimination by macrophages^18^. In short, the versatility of this third-generation of engineered bacteria holds great potential for cancer diagnosis and therapy.

The clinical translation of bacterial therapy has been largely restricted by the lack of in vivo controllability, which may lead to unavoidable side effects and unsatisfactory treatment efficacy. The foundation of the safety and effectiveness of bacterial therapy is the spatiotemporal manipulation of the in vivo behavior of engineered bacteria, including the control of bacterial colonization, reproduction, payload synthesis, and drug release behavior^19^. Precisely modifying tumor-homing bacteria with nanomaterials provides a way to achieve real-time and spatiotemporal manipulation in vivo^8^. For example, up-conversion nanomaterial microgel-modified *E. coli Nissle* 1917 can convert near-infrared light into blue light to activate blue light-sensitive plasmids transfected into the recombinant bacteria^20^. This nano-optogenetics technology enabled the in vivo manipulation of engineered bacteria by near-infrared light to realize controlled and effective colonization of *E. coli* in the gut. However, even though the tissue-penetrability of near-infrared light is greater than that of blue light, the light penetration is still limited (only a few millimeters)^21,22^, which restricts the clinical application of light-manipulation. Therefore, there is an urgent need to develop novel engineered bacteria that can be spatiotemporally manipulated in deep tissue by exogenous signals with strong tissue-penetrability. Alternating magnetic field (AMF) is an ideal signal for manipulating bacteria due to its virtually limitless tissue-penetrating capability and excellent biosafety^22–24^. However, a specific method for AMF manipulation of gene expression of tumor-homing bacteria has remained elusive.

Herein, we describe an AMF-manipulated tumor-homing bacteria (AMF-Bac), constructed by modifying genetically engineered *E. coli BL21* attenuated strain with a Fe_3_O_4_@lipid nanocomposite. We modularly design and fabricate the AMF-Bac utilizing both genetic engineering and nanotechnology. Similar to machinery robots, the AMF-Bac is equipped and assembled with five functional modules of a robotic system: “active navigation”, “signal decoding”, “signal feedback”, “signal process” and “signal output”. Upon colon-specific administration, the active navigation module provides AMF-Bac with active tumor-targeting to orthotopic colon tumors. After reaching the tumor tissue, the Fe_3_O_4_ nanoparticles (NPs) in the signal decoding module enables AMF-Bac to receive magnetic signals and transform them into heat signals, which initiate the signal feedback and signal process modules. Heat-induced fluorescent dye release from the signal feedback module realizes fluorescence imaging and monitoring of signal decoding behavior, while the heat signals initiate the expression of bacteria lysis proteins which are under the control of a heat-sensitive promoter in the signal process module. Next, the AMF-Bac lyses and releases an immunotherapeutic drug, anti-CD47 nanobody (CD47nb), that is pre-expressed and stored in the signal output module of the AMF-Bac. Eventually, the AMF-Bac enables tumor-targeted delivery of CD47nb to avoid hematologic toxicity and increase CD47nb accumulation in tumors for precision tumor immunotherapy.

## Results

### Design of the AMF-Bac and assembly of the active navigation module

**Fig. 1:**
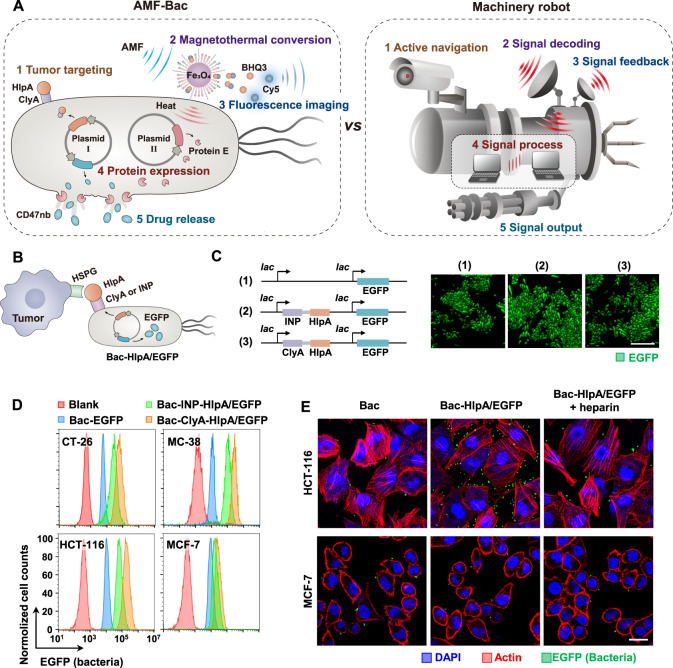
Design of AMF-Bac and its analogy with a machinery robot, and the assembly of the active navigation module. **A** Illustration of AMF-Bac, comprising five modules: active navigation, signal decoding, signal feedback, signal process, and signal output. The five modules respectively carry out the physiological functions of tumor targeting, magnetothermal conversion, fluorescence imaging, protein expression, and drug release. **B** Diagram illustrating the tumor-targeting ability of Bac-HlpA/EGFP. HlpA is displayed on the surface of Bac-HlpA/EGFP for targeting to the HSPG on colon tumor cells, through two different surface display scaffolds, ClyA and INP. **C** Expression plasmid design and EGFP expression verification. Bacteria were transformed with the indicated plasmids, including pACYC-EGFP (1), pACYC-INP-HlpA-EGFP (2), and pACYC-ClyA-HlpA-EGFP (3), and the corresponding engineered bacteria were termed Bac-EGFP, Bac-INP-HlpA/EGFP and Bac-ClyA-HlpA/EGFP, respectively. *lac* represents the IPTG-inducible promotor. Successful expression of EGFP was verified by observation under a confocal laser scanning microscope (CLSM). Scale bar, 10 μm. **D**, **E** The tumor-targeting ability of Bac-HlpA/EGFP. Engineered bacteria were incubated with the indicated cell lines for 2 h at 4 °C, and the bacterial fluorescence intensities of the cells were examined using flow cytometry (**D**). The binding of bacteria onto cells was also directly visualized using CLSM (**E**). Bacteria were tracked by their expression of EGFP (green), while tumor cell actin was stained with phalloidine (red). Cell nuclei were stained with DAPI (blue). Scale bar, 40 μm. These experiments (C-E) were repeated three times independently with similar results. Source data are provided as a Source Data file.

As illustrated in Fig. 1A, the AMF-Bac is composed of five modules, including active navigation, signal decoding, signal feedback, signal process, and signal output. The five modules carry out their respective functions of tumor targeting, magnetothermal conversion, fluorescence imaging, protein expression, and drug release.

We chose heparan sulfate proteoglycans (HSPG), which are known to be overexpressed in colon cancer^25^, as the tumor target. Histone-like protein A (HlpA) has a high affinity for HSPG^25^. Therefore, we engineered bacteria to express HlpA on their surface, to form the active navigation module, through two different surface display scaffolds, ClyA (*E. coli* Cytolysin A)^26^ and ice nucleation protein (INP)^25^ (Fig. 1B). *E. coli BL21* were transformed with the plasmid pACYC-ClyA-HlpA-EGFP (or pACYC-INP-HlpA-EGFP), which used pACYCDuet-1, which contains two isopropyl-β-d-thiogalactoside (IPTG)-inducible promoters, as the plasmid backbone enabling the simultaneous co-expression of two separate proteins: the fusion protein ClyA-HlpA (or INP-HlpA) and the enhanced green fluorescent protein (EGFP) (Fig. 1C). For examining the tumor-targeting ability of the bacteria, we first evaluated the expression levels of HSPG in different tumor cell lines using confocal laser scanning microscopy (CLSM) after the immunofluorescence staining of HSPG (Supplementary Fig. 1), which showed that HSPG was highly expressed on three colon tumor cell lines (mouse CT-26, MC-38 and human HCT-116), compared with the relatively low expression in the breast cancer cell line, MCF-7. Next, we incubated the tumor cells with EGFP-expressed (Bac-EGFP) or HlpA/EGFP-co-expressed bacteria (Bac-HlpA/EGFP), and examined the bacterial EGFP fluorescence in the tumor cells by flow cytometry. In all three colon tumor cell lines, the fluorescence intensities in the two Bac-HlpA/EGFP groups were higher than that in the Bac-EGFP group (Fig. 1D). In addition, the fluorescence intensities in the ClyA-HlpA-expressing group (Bac-ClyA-HlpA/EGFP) were higher than that in the INP-HlpA group (Bac-INP-HlpA/EGFP; Fig. 1D). Therefore, the ClyA display system was adopted for subsequent experiments to establish Bac-HlpA/EGFP. The fluorescence intensities of Bac-HlpA/EGFP in CT-26, MC-38 and HCT-116 cells were also higher than those in MCF-7 cells (Fig. 1D), demonstrating the potential of HlpA to target colon cancer cells. We also directly visualized the binding of the bacteria to tumor cells by CLSM, which revealed markedly greater Bac-HlpA/EGFP associated with HCT-116 cells than with MCF-7 cells (Fig. 1E). The binding of Bac-HlpA/EGFP to HCT-116 cells could be blocked by excess heparin (Fig. 1E), which is a specific ligand of HSPG, further proving the specificity of the binding between the HlpA in the active navigation module and the HSPG on colon tumor cells.

### Assembly of the signal decoding module

**Fig. 2:**
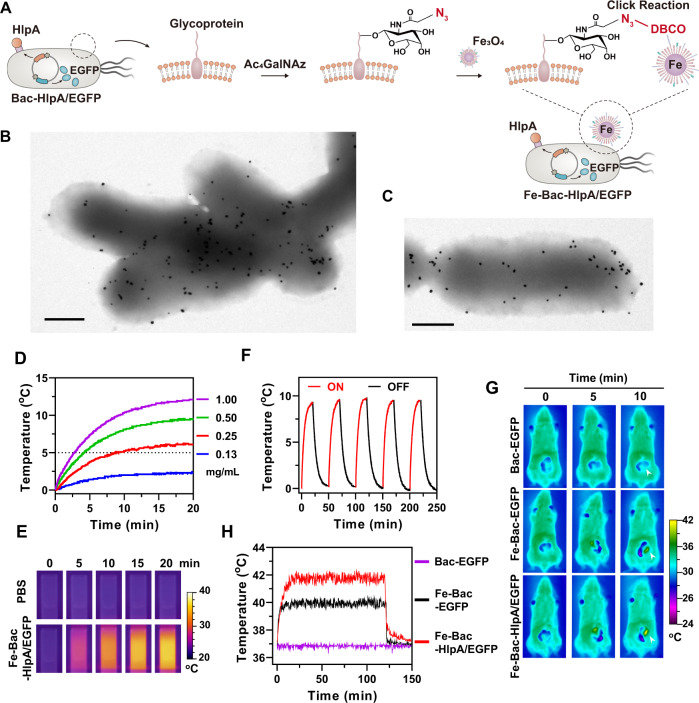
Assembly of the signal decoding module. **A** Diagram of site-specific coupling of DBCO-Fe_3_O_4_ onto the tumor-homing bacteria. The reactive functional groups (-N_3_) were introduced onto the bacterial surface via metabolic oligosaccharide engineering, through culturing bacteria with a non-natural form of galactosamine (Ac_4_GalNAc). DBCO-Fe_3_O_4_ were conjugated onto the N_3_-modified bacteria through the click chemistry reaction between DBCO and -N_3_ moieties. **B**, **C** TEM images of bacteria modified with Fe_3_O_4_ NPs. Site-specific (**B**) and non-site-specific (**C**) labeling methods were employed to conjugate Fe_3_O_4_ NPs onto bacteria. Scale bar, 0.5 μm. **D** Temperature elevation of aqueous solutions of Fe-Bac-HlpA/EGFP at different Fe_3_O_4_ concentrations (0.13–1.00 mg/mL) during treatment with AMF at a fixed frequency of 310 kHz and intensity of 14.6 kA/m. **E** Infrared imaging of Fe-Bac-HlpA/EGFP solutions (1.00 mg/mL Fe_3_O_4_) upon AMF treatment (310 kHz and 14.6 kA/m) for the indicated time intervals (0–20 min). **F** Temperature changes of Fe-Bac-HlpA/EGFP solutions (0.5 mg/mL Fe_3_O_4_) during five cycles of AMF treatment (ON; 310 kHz and 14.6 kA/m) and natural cooling-down (OFF). **G** Infrared imaging of BALB/c mice with CT-26 colonic orthotopic xenografts upon AMF treatment (310 kHz and 23.8 kA/m) for the indicated time intervals (0–10 min). The mice were treated with the indicated engineered bacteria (1 × 10^8^ CFU) by colon-specific administration at 24 h prior to AMF treatment, and laparotomized before infrared imaging. The white arrows indicate the tumor. **H** Intratumoral temperature of CT-26 colonic orthotopic xenografts upon AMF treatment (310 kHz and 23.8 kA/m) for 120 min. The temperature in tumors was monitored by inserting an electronic needle thermometer into the tumor. These experiments (B-H) were repeated three times independently with similar results. Source data are provided as a Source Data file.

To assemble a signal decoding module that can receive and convert magnetic signals, we synthesized oleic acid-stabilized Fe_3_O_4_ NPs. Next, the Fe_3_O_4_ NPs was coated with a lipid monolayer to form Fe_3_O_4_@lipid nanocomposites, and carboxyl (-COOH) or dibenzocyclooctyne (-DBCO) moieties were introduced onto the surface of the lipid monolayer of the Fe_3_O_4_@lipid nanocomposites (Fe_3_O_4_-COOH or Fe_3_O_4_-DBCO; Supplementary Fig. 2), for subsequent conjugation onto the Bac-HlpA/EGFP through both non-site-specific or site-specific labeling methods, respectively. The traditional non-site-specific labeling method utilizes the free amino groups in bacterial outer membrane proteins to react with the Fe_3_O_4_-COOH, while, for the site-specific labeling method, the azide moieties (-N_3_) were introduced onto the bacterial surface via the metabolic oligosaccharide engineering^27^ (Supplementary Fig. 3), and the Fe_3_O_4_-DBCO were conjugated onto the bacteria surface through the click chemistry reaction between -DBCO and -N_3_ moieties (Fig. 2A)^28^. As shown in the transmission electron microscopy (TEM) images in Supplementary Fig. 4, no Fe_3_O_4_-DBCO was found on the Bac-HlpA/EGFP without N_3_ moieties, but abundant Fe_3_O_4_-DBCO were conjugated onto Bac-HlpA/EGFP with N_3_ moieties (Fig. 2B). Abundant Fe_3_O_4_-COOH could also be conjugated onto Bac-HlpA/EGFP by the non-site-specific labeling method (Fig. 2C). However, the site-specific labeling method had significantly less influence on bacterial growth compared with the non-site-specific method (Supplementary Fig. 5), and the site-specific labeling method did not induce cross-linking between bacteria (Supplementary Fig. 6). Therefore, we adopted the site-specific labeling method to construct Fe_3_O_4_ NP-decorated bacteria with expression of HlpA and EGFP (Fe-Bac-HlpA/EGFP) for subsequent experiments.

Next, we tested whether the engineered bacteria equipped with a signal decoding module can receive and convert magnetic signals into heat signals. Upon AMF treatment, the temperature of an aqueous suspension of Fe-Bac-HlpA/EGFP significantly increased by as much as 12 °C (Fig. 2D, E), exhibiting the efficacy of the magnetothermal effect. The temperature changes of the Fe-Bac-HlpA/EGFP suspension remained stable throughout five cycles of AMF treatments and natural cooling (Fig. 2F), exhibiting the stability of the magnetothermal effect. By adjusting the AMF intensity, the temperature elevation could be precisely controlled from 0 to 5 °C (Supplementary Fig. 7), exhibiting the controllability of magnetothermal effect.

The signal decoding function (magnetothermal effect) was also evaluated in vivo using BALB/c mice with CT-26 colonic orthotopic xenografts (Supplementary Fig. 8). Tumor-bearing mice were treated with Fe-Bac-HlpA/EGFP (1 × 10^8^ colony forming units [CFU]) by colon-specific administration 24 h before AMF treatment. Bac-EGFP and Fe-Bac-EGFP were used as a non-signal decoding control and non-tumor-targeting control, respectively. After laparotomy, whole-body infrared imaging revealed that only the temperature at the tumor sites was elevated with a 10 min AMF treatment, while the temperature of other organs remained unchanged (Fig. 2G). The temperature in the tumors was also precisely monitored by inserting an electronic thermometer needle into the tumor. During the long-term AMF treatment, the Bac-EGFP without the signal decoding module did not generate heat signals at the tumor site, while the Fe-Bac-HlpA/EGFP achieved a significant temperature elevation (~2 °C) over controls, owing to the HlpA-based tumor-targeting ability. Moreover, the temperature in the tumor could be maintained at 42 °C for at least 120 min (Fig. 2H). In conclusion, the Fe_3_O_4_-based signal decoding module in the engineered bacteria was proved to be able to receive and convert magnetic signals into heat signals in vitro and in vivo, thereby raising the temperature at the tumor site to 42 °C.

### Assembly of the signal feedback module

**Fig. 3:**
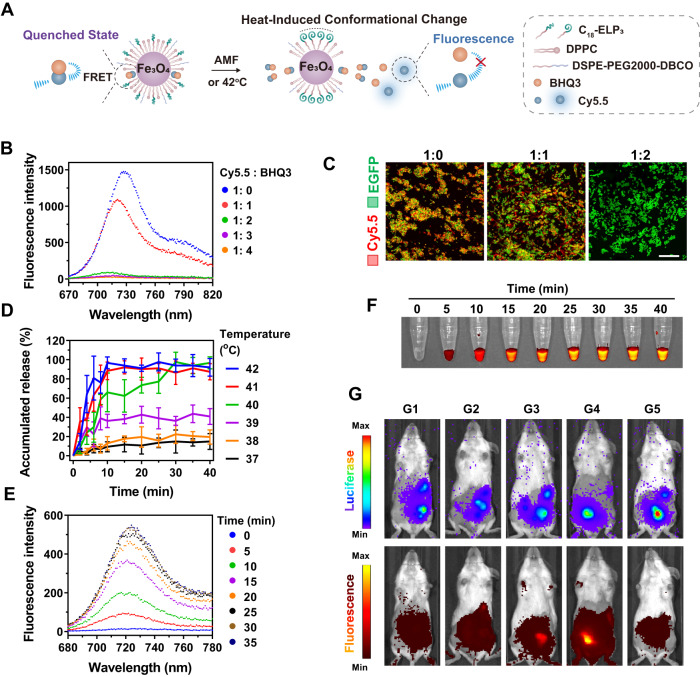
Assembly of signal feedback module. **A** Illustration of signal feedback module for AMF-responsive fluorescence imaging. Fe_3_O_4_ NPs was coated with a monolayer of LTSL (LTSL@Fe), which was composed of DSPE-PEG2000-DBCO and temperature-sensitive lipids, including DPPC and C_18_-ELP_3_. The fluorescent dye, Cy5.5, and its black-hole quencher, BHQ3, were co-loaded into the LTSL monolayer (LTSL@Fe/C/B). In the LTSL@Fe/C/B, the fluorescence of Cy5.5 was quenched by BHQ3, which is recovered by the heat-induced conformational change and Cy5.5 release. **B** Fluorescence emission spectra of LTSL@Fe/C/B at different molar ratios of Cy5.5 and BHQ3. **C** Observation of LTSL@Fe/C/B-Bac-HlpA/EGFP at different molar ratios of Cy5.5 and BHQ3 using CLSM. Bacteria were tracked by EGFP expression. Scale bar, 20 μm. **D** The percentage of accumulated release of Cy5.5 from LTSL@Fe/C/B-Bac-HlpA/EGFP upon water-bath treatment for 40 min at the indicated temperatures (37–42 °C). The data are shown as the mean ± SD (*n* = 3 independent experiments). **E**, **F** Fluorescence emission spectra (**E**) and fluorescence imaging (**F**) of LTSL@Fe/C/B-Bac-HlpA/EGFP after AMF treatment (310 kHz and 23.8 kA/m) for the indicated time intervals. **G** Fluorescence imaging of mice with CT-26-luc colonic orthotopic xenografts after the indicated treatments: G1, Fe/C/B-Bac-HlpA/EGFP + AMF; G2, LTSL@Fe/C/B-Bac-EGFP; G3, LTSL@Fe/C/B-Bac-EGFP + AMF; G4, LTSL@Fe/C/B-Bac-HlpA/EGFP + AMF; G5, PBS + AMF. The mice were treated with the indicated engineered bacteria (1 × 10^8^ CFU) via colon-specific administration at 24 h before AMF treatment. The AMF treatment (310 kHz and 23.8 kA/m) lasted for 30 min prior to fluorescence imaging. The tumor sites were tracked by bioluminescence imaging of luciferase expression in the tumor cells. For Fe/C/B-Bac-HlpA/EGFP, Fe_3_O_4_ NPs were coated with the temperature-insensitive lipid DSPC (distearoyl phosphatidylcholine), rather than LTSL. These experiments (**B**, **C**, **E**–**G**) were repeated three times independently with similar results. Source data are provided as a Source Data file.

A signal feedback module based on a low temperature-sensitive lipid (LTSL) was assembled into the engineered bacteria to monitor the signal decoding process (Fig. 3A). Briefly, the Fe_3_O_4_ NPs were coated with a monolayer of LTSL (LTSL@Fe), which was composed of DSPE-PEG2000-DBCO (for conjugation to the Fe-Bac-HlpA/EGFP) and temperature-sensitive lipids, including DPPC (dipalmitoyl phosphatidylcholine) and C_18_-ELP_3_ (a fatty acid conjugated elastin-like polypeptide)^29^. In addition, the fluorescent dye Cy5.5 and its black-hole quencher, BHQ3, were co-loaded into the LTSL monolayer (LTSL@Fe/C/B)^30,31^. In the LTSL@Fe/C/B-modified Bac-HlpA/EGFP (LTSL@Fe/C/B-Bac-HlpA/EGFP), the fluorescence of Cy5.5 was quenched by BHQ3. When the signal decoding module received and converted the magnetic signals into heat signals, the LTSL monolayer underwent a conformational change and the Cy5.5 was released from the quencher to recover fluorescence, which could be used as a feedback signal for the magnetothermal process (Fig. 3A).

Next, we adjusted and evaluated the signal feedback module. The molar ratio of Cy5.5 and BHQ3 was optimized to a 1: 2 ratio so that the Cy5.5 fluorescence was almost entirely quenched by BHQ3 in LTSL@Fe/C/B (Fig. 3B) and LTSL@Fe/C/B-Bac-HlpA/EGFP (Fig. 3C), when examined by fluorescence emission spectra and CLSM, respectively. A 42 °C water-bath treatment induced almost 100% Cy5.5 release from LTSL@Fe/C/B within 10 min, while only 15% Cy5.5 was released with 37 °C treatment (Fig. 3D). Upon AMF treatment, the Cy5.5 fluorescence in LTSL@Fe/C/B also rapidly appeared within 25 min in vitro (Fig. 3E, F). The AMF-triggered fluorescence imaging was finally examined in vivo using mice with luciferase-expressing CT-26 (CT-26-luc) colonic orthotopic xenografts. After the colon-specific administration of LTSL@Fe/C/B-modified Bac-EGFP (LTSL@Fe/C/B-Bac-EGFP) without AMF treatment, there was no apparent Cy5.5 fluorescence at the tumor site (Fig. 3G). However, a strong Cy5.5 fluorescence appeared at the tumor site in the LTSL@Fe/C/B-Bac-EGFP + AMF group, demonstrating the AMF-responsiveness of fluorescence imaging. Importantly, the Cy5.5 fluorescence was stronger in the LTSL@Fe/C/B-Bac-HlpA/EGFP + AMF group than in the LTSL@Fe/C/B-Bac-EGFP + AMF group, owing to the tumor-targeting effect of the HlpA (Fig. 3G). Therefore, the signal feedback module (AMF-responsive imaging) could be used to monitor the distribution and signal decoding behavior (magnetothermal effect) of the engineered bacteria.

### Assembly of signal process and signal output modules

CD47 is a transmembrane protein known as a “don’t eat me” signal, which is ubiquitously expressed on all human cells^32^. The cognate receptor for CD47, signal regulatory protein α (SIRPα), is expressed on phagocytic macrophages and dendritic cells (DCs)^33^. The CD47-SIRPα interaction prevents phagocytosis, acting as an important phagocytosis checkpoint^34^. Increased expression of CD47 has been observed in nearly all types of tumors, protecting tumor cells from phagocytosis and clearance^32^. The systemic blockade of the CD47-SIRPα axis using anti-CD47 antibodies has been shown to elicit an antitumor effect in preclinical models, leading to multiple clinical trials (ongoing at the time of writing)^33^. However, the anti-CD47 antibodies often bind to non-tumor cells, especially red blood cells, due to the ubiquitous expression of CD47, which leads to hematologic toxicity and limited antitumor efficacy^32^. The preferential blockage of CD47 on tumor cells remains a significant challenge. The AMF-Bac we describe can enable tumor-targeted delivery of CD47nb to avoid hematologic toxicity and increase CD47nb accumulation in tumors.

**Fig. 4:**
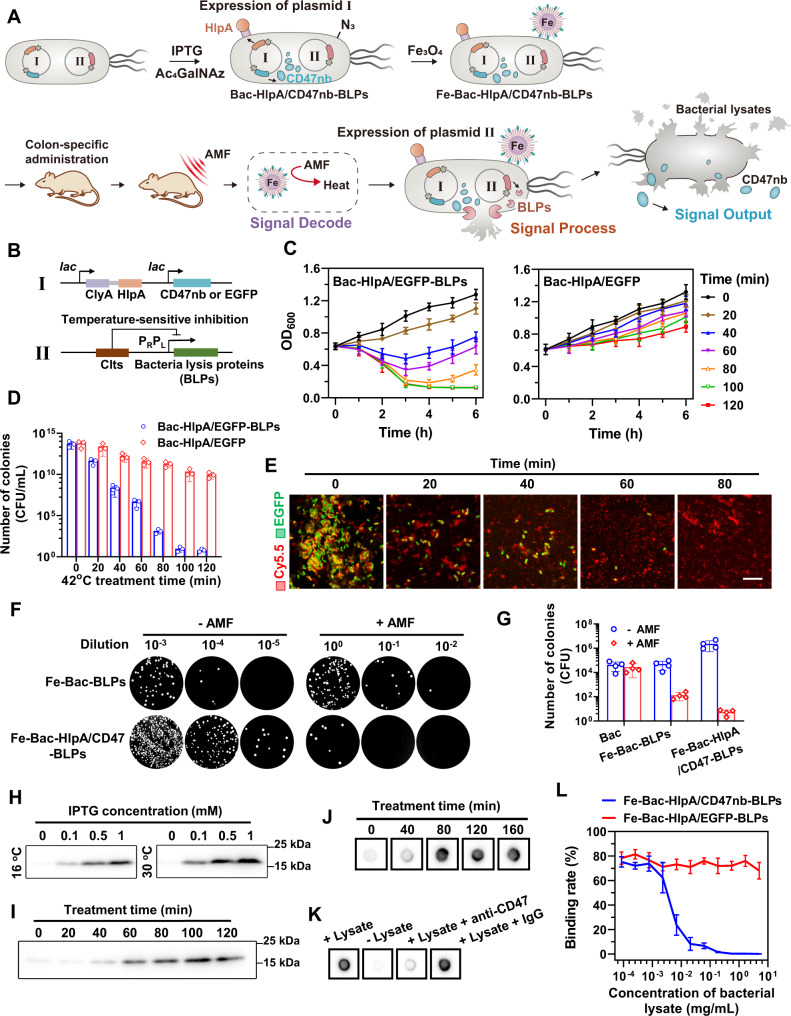
Assembly of signal process and signal output modules. **A** Illustration of the assembly process and working principle of AMF-Bac. **B** The design of plasmids I and II. **C** The growth of Bac-HlpA/EGFP-BLPs and Bac-HlpA/EGFP after 42 °C treatment for the indicated times (0–120 min), as monitored by OD_600_. The data are shown as the mean ± SD (n = 3 independent experiments). **D** Bacteria from the different groups at 6 h in **C** were seeded on agar plates, and the number of colonies formed was counted. CFU, colony forming unit. The data are shown as the mean ± SD (*n* = 3 independent experiments). **E** The lysis of Fe-Bac-HlpA/EGFP-BLPs after AMF treatment (310 kHz and 23.8 kA/m) for the indicated times (0–80 min), as observed by CLSM. The bacteria were tracked by their EGFP expression, and the Fe_3_O_4_ NPs on the bacterial membrane were labeled with Cy5.5. Scale bar, 10 μm. **F**, **G** The lysis of engineered bacteria induced by AMF treatment in vivo. Mice bearing CT-26-luc xenografts were treated with engineered bacteria. The number of live bacteria in the tumor was measured by the spread plate method (**G**). The data (**G**) are shown as the mean ± SD (*n* = 4 mice). **H** Optimization of the inducible expression conditions for overnight expression of CD47nb in the Fe-Bac-HlpA/CD47nb-BLPs. **I** The release of CD47nb (containing a Myc tag) from Fe-Bac-HlpA/CD47nb-BLPs upon 42 °C treatment for the indicated times (0–120 min) by western blot analysis using an anti-Myc tag antibody. **J** The release of CD47nb from Fe-Bac-HlpA/CD47nb-BLPs after AMF treatment (310 kHz and 23.8 kA/m) for the indicated times (0–160 min), as examined by dot blotting. **K** The affinity of the released CD47nb to the CD47 protein was verified by dot blotting. **L** Competition binding assay using the anti-CD47 antibody to examine the affinity of released CD47nb to CD47 in CT-26 cells. The data are shown as the mean ± SD (*n* = 3 independent experiments). These experiments (**E**, **H**–**K**) were repeated three times independently with similar results. Source data are provided as a Source Data file.

Figure 4A presents a schematic illustration of the signal process and signal output modules of the engineered bacteria. In the first part of the active navigation module design, we used the pACYCDuet-1 plasmid to achieve co-expression of the tumor-targeting fusion protein, ClyA-HlpA, and the indicator protein, EGFP (pACYC-ClyA-HlpA-EGFP), which could be induced by IPTG. To establish the signal output module, the indicator protein, EGFP, was replaced with the antitumor protein, CD47nb (pACYC-ClyA-HlpA-CD47nb), which we designated “plasmid I” (Fig. 4B). Nanobody is the antigen-binding fragment of heavy-chain antibodies (HcAbs) that circulate in sera of camelids, which is also called VHH (variable domain of the heavy chain from HcAbs) or single domain antibody. As the smallest antigen-binding unit of an antibody, nanobody exhibits full antigen-binding capacity with high specificity and affinity^35^. The simple structure and small size (15 kDa) of nanobody enables its efficiently recombinant expression in bacteria, while the traditional antibodies with multiple subunits and large size (150 kDa) can hardly be expressed with correct protein folding in prokaryotic expression system^36^.

We next employed the pBV220 plasmid to establish the signal process module. The heat-sensitive promoter in pBV220 permits gene expression only when the temperature rises to 42 °C; the bacteria lysis proteins (BLPs) were inserted into this plasmid (pBV220-BLPs), which we designated “plasmid II” (Fig. 4B). As shown the assembly process illustration in Fig. 4A, we co-transformed bacteria with plasmids I and II, and added IPTG to induce the expression of the active navigation module, ClyA-HlpA, and signal output module, CD47nb, which was already expressed and stored in the engineered bacteria (Bac-HlpA/CD47nb-BLPs). In addition, we performed metabolic oligosaccharide engineering to introduce N_3_ moieties into the bacterial surface for conjugation of the signal decoding module, Fe_3_O_4_-DBCO, to form the final AMF-Bac (Fe-Bac-HlpA/CD47nb-BLPs). After guidance of the AMF-Bac to the tumor site by the active navigation module, and application of the AMF, the signal decoding module (magnetothermal conversion by Fe_3_O_4_ NPs), signal process module (heat-induced BLPs expression), and signal output module (bacterial lysis and cargo release) worked sequentially, ultimately releasing CD47nb into the tumor tissue. In the following studies, Fe-Bac-HlpA/EGFP-BLPs without signal output module (CD47nb) and Fe-Bac-HlpA/CD47nb without signal process module (BLPs) were used as the control groups for their comparison with AMF-Bac.

We examined the heat-induced bacteria lysis function of the signal process module. Bac-HlpA/EGFP-BLPs and Bac-HlpA/EGFP were cultured at 30 °C, and their growth rates were monitored by OD_600 nm_. The growth rate of Bac-HlpA/EGFP-BLPs had little difference from that of Bac-HlpA/EGFP (Supplementary Fig. 9), demonstrating that the plasmid II pBV220-BLPs could hardly work at 30 °C. Upon a 42 °C treatment, the growth rate (Fig. 4C) and colony formation ability (Fig. 4D) of the bacteria transfected with plasmid I and II (Bac-HlpA/EGFP-BLPs) were significantly inhibited, compared with the bacteria transfected with plasmid I (Bac-HlpA/EGFP), which is consistent with heat-induced bacteria lysis.

We next evaluated the AMF-responsive bacteria lysis of the AMF-Bac in vitro and in vivo. As shown in the CLSM images presented in Fig. 4E, AMF treatment effectively induced the lysis of Fe-Bac-HlpA/EGFP-BLPs, as indicated by the gradual disappearance of EGFP fluorescence. Similarly, mice bearing CT-26 colonic orthotopic xenografts treated with Fe-Bac-HlpA/EGFP-BLPs by colon-specific administration exhibited a significantly decreased number of live bacteria in tumors (Fig. 4F, G). The AMF-responsive intratumoral release of CD47nb from AMF-Bac was examined in vivo. Mice bearing CT-26 colonic orthotopic xenografts were administrated with Fe-Bac-HlpA/CD47-BLPs followed by AMF treatment. Bacterial membrane of Fe-Bac-HlpA/CD47-BLPs in the frozen sections of tumors was stained with FITC-conjugated anti-*E. coli* O + *E. coli* K antibodies, which react with O or K antigenic serotypes of *E. coli* membrane. Without the AMF treatment, the FITC fluorescence exhibited spot-like shapes in tumor tissue sections (Supplementary Fig. 10), indicating intact bacterial membranes. However, The FITC fluorescence was weakly dispersed in large areas rather than exhibiting spot-like shapes after the AMF treatment, possibly due to the lysis of bacteria and the crushing of bacterial membrane. Meanwhile, high intensity of CD47nb immunofluorescence largely distributed in tumor tissue sections after the AMF treatment, and the relative fluorescence intensity was 17.5 folds compared with the control without AMF treatment (Supplementary Fig. 11), demonstrating the release of CD47nb from those bacteria. These results demonstrate that the magnetic manipulation of the AMF-Bac may be used to precisely achieve the control of bacteria lysis at the tumor site.

The inducible expression of CD47nb in the Fe-Bac-HlpA/CD47nb-BLPs was optimized to overnight culture with 1 mM IPTG at 30 °C (Fig. 4H). Treatment at 42 °C or with AMF efficiently triggered the release of CD47nb from Fe-Bac-HlpA/CD47nb-BLPs (Fig. 4I, J). The released CD47nb had a high affinity for CD47 protein, and the binding of released CD47nb to CD47 was blocked by an anti-CD47 antibody (Fig. 4K), demonstrating the specificity of the released CD47nb. The binding competition assay using the anti-CD47 antibody showed that the released CD47nb also had a specific affinity for CD47 in CT-26 cells in vitro (Fig. 4L), further demonstrating the bioactivity of the released CD47nb.

We then determined the interaction between CD47 on tumor cells and SIRPα on macrophages in vitro and in vivo. The phagocytosis efficacy of bone marrow-derived macrophages (BMDMs) was examined by flow cytometry in vitro. Green fluorescent protein (GFP)-transfected CT-26 cells (CT-26^GFP+^) were respectively incubated with PBS (G-I), Fe-Bac-HlpA/CD47nb-BLPs (G-II), the lysis of Fe-Bac-HlpA-BLPs (G-III), or the lysis of Fe-Bac-HlpA/CD47nb-BLPs (G-IV), followed by the incubation with BMDMs. The proportions of GFP^+^ cells in F4/80^+^ BMDMs were analyzed by flow cytometry to identify the BMDMs that engulfed CT-26^GFP+^ cells (Supplementary Fig. 12A). Only a small portion of BMDMs (1.21%, Supplementary Fig. 12B, C) phagocytosed CT-26^GFP+^ cells upon the addition of PBS. The average proportions of BMDMs that engulfed CT-26^GFP+^ cells were slightly increased to 3.65% and 5.35% upon the addition of Fe-Bac-HlpA/CD47nb-BLPs or the lysis of Fe-Bac-HlpA-BLPs (Supplementary Fig. 12C), possibly because the abundant immunogenic substances (e.g., flagella) could activate macrophages and enhance their phagocytosis^37^. Compared with G-III, the proportions of BMDMs that engulfed CT-26^GFP+^ cells in G-IV were significantly increased to 15.03% (***P = 0.0002, Supplementary Fig. 12C), which demonstrated that CD47nb in AMF-Bac could enhance the phagocytosis of BMDMs in vitro. We further conducted in vivo antitumor therapy to investigate whether macrophage depletion could influence the therapeutic effects of AMF-Bac. Mice bearing CT-26-luc subcutaneous tumors were randomized into six groups with different treatments (Supplementary Fig. 13A): G1, PBS; G2, rat IgG1 isotype control (Rat IgG); G3, rat anti-mouse colony-stimulating factor (CSF1) neutralizing antibody (Anti-CSF1); G4, Fe-Bac-HlpA/CD47nb-BLPs + AMF; G5, Fe-Bac-HlpA/CD47nb-BLPs + AMF + Rat IgG; G6, Fe-Bac-HlpA/CD47nb-BLPs + AMF + Anti-CSF1. The selective removal of tumor-associated macrophages was achieved by the intratumoral injection of Anti-CSF1 every 3 days, and Rat IgG was employed as the isotype control^38,39^. The comparison of G4 and G5 showed that there was no significant different of their tumor weight (Supplementary Fig. 13B, C) and overall survival time (Supplementary Fig. 13D), demonstrating the Rat IgG had little influence on the therapeutic effects of AMF-Bac. Importantly, the tumor weight of G6 was 2.23 folds compared with G5 with a significant difference (Supplementary Fig. 13C). The overall survival time of G6 was also significantly higher than that of G5 (Supplementary Fig. 13D), demonstrating that macrophage depletion could decrease the therapeutic effects of AMF-Bac, which was possibly resulted from the disability of CD47nb to block the interaction between CD47 on tumor cells and SIRPα on macrophages.

### Therapeutic effects of AMF-Bac in the colonic orthotopic model

**Fig. 5:**
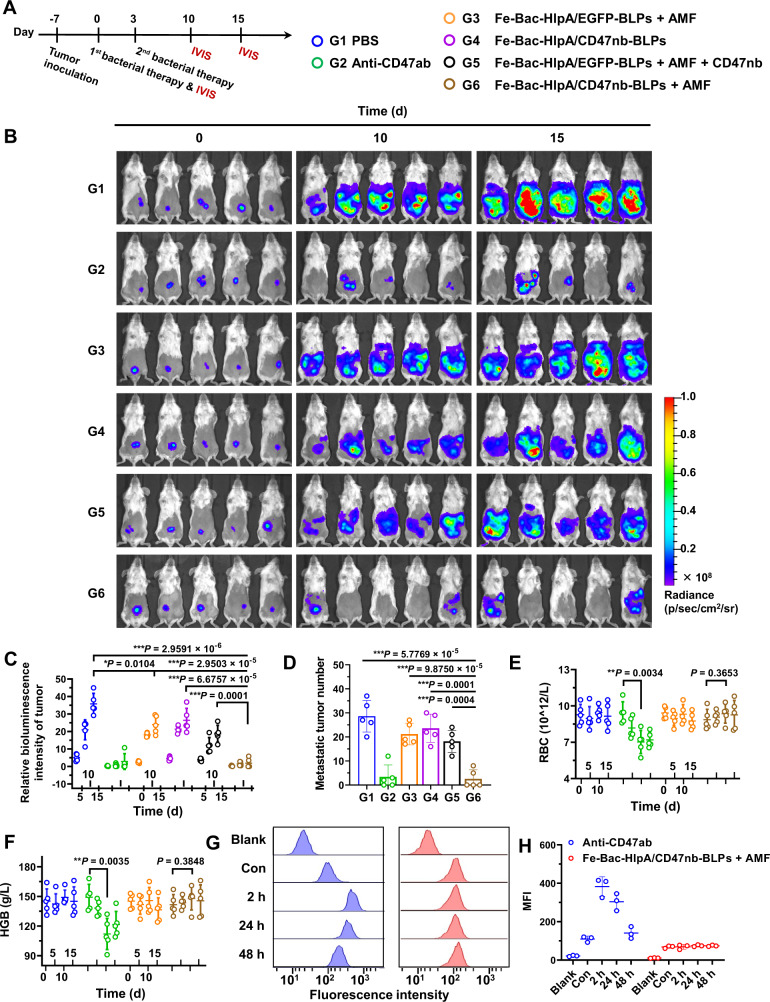
Therapeutic effects of the engineered bacteria in the colonic orthotopic model. **A** Scheme and grouping of in vivo therapy (*n* = 5). The colons of BALB/c mice were inoculated with CT-26-luc cells (1 × 10^5^ cells/mouse) on day -7, and the mice were treated with the indicated engineered bacteria (1 × 10^8^ CFU) by colon-specific administration on days 0 and 3, followed by AMF treatment (310 kHz and 23.8 kA/m) for 80 min at 24 h after colon administration. An anti-CD47 antibody (Anti-CD47ab; 20 mg/kg) and CD47nb (20 mg/kg) were i.p. injected on days 0 and 3 in G2 and G5, respectively. **B** Bioluminescence imaging to monitor tumor growth on days 0, 10, and 15. **C** Semi-quantitative results of the bioluminescence intensity of the tumor regions shown in panel B (*n* = 5 mice). The bioluminescence intensities of each mouse at days 10 and 15 were normalized to day 0. **D** The number of metastatic tumors in the abdomen were counted on day 15 (*n* = 5 mice). **E**, **F** The changes in red blood cell count (RBC; **E**) and hemoglobin (HGB; **F**) during therapy (*n* = 5 mice). **G** Flow cytometry analysis of Anti-CD47ab (blue) or CD47nb (red) on RBCs in BALB/c mice bearing CT-26-luc colonic orthotopic xenografts after a single treatment with Anti-CD47ab or Fe-Bac-HlpA/CD47nb-BLPs + AMF. The Anti-CD47ab and CD47nb were detected using a fluorescein-labeled antibody against IgG and a nanobody, respectively. RBC without staining was used as the blank, and RBCs from healthy mice without treatments was used as control (Con). **H** Quantitative results of mean fluorescence intensity (MFI) in panel G (*n* = 3 independent experiments). The data (**C**–**F**, **H**) are shown as the mean ± SD. Statistical analysis was performed by a two-tailed unpaired *t* test. ^*^P < 0.05; ^**^P < 0.01^; ***^P < 0.001. Source data are provided as a Source Data file.

We evaluated the therapeutic effects of AMF-Bac using BALB/c mice bearing CT-26-luc colonic orthotopic xenografts. To optimize the treatment dosage, the intratumoral number of live bacteria, and the intratumoral CD47nb concentration, were examined after the administration of different dosages of Fe-Bac-HlpA/EGFP-BLPs (Supplementary Fig. 14). The dose of 1 × 10^8^ CFU per mouse was adopted, which could provide a sufficient number of AMF-Bac for targeting tumors. Seven days after tumor inoculation (designated as day 0), the mice were randomized into six groups: G1, PBS; G2, anti-CD47 antibodies (Anti-CD47ab); G3, Fe-Bac-HlpA/EGFP-BLPs + AMF; G4, Fe-Bac-HlpA/CD47nb-BLPs; G5, Fe-Bac-HlpA/EGFP-BLPs + AMF + CD47nb; G6, Fe-Bac-HlpA/CD47nb-BLPs + AMF. Treatments were administrated twice, on days 0 and 3 (Fig. 5A). Bioluminescence imaging was performed to monitor tumor growth after the various treatments (Fig. 5B). The tumor growth in G3 was lower than that in the control group (G1), with an inhibition rate of 33.0%, as calculated from the change in bioluminescence intensity on day 15 (Fig. 5C), indicating that the bacterial lysates in tumor tissue may slightly inhibit tumor growth. An strong inhibition of the bioluminescence intensity on day 15 (94.7% compared to G1) was achieved in G6, with 60% of the mice fully relieved of tumor burden (Fig. 5C). The number of abdominal metastatic tumors in G6 was decreased by 91.0% compared with G1 (Fig. 5D). G4 exhibited a less effective inhibition of tumor growth compared with G6, likely owing to the lack of AMF treatment, thus supporting the in vivo AMF-responsiveness of Fe-Bac-HlpA/CD47nb-BLPs. G6 also exhibited superior therapeutic efficacy compared to G5 (Fig. 5C), demonstrating the advantage of tumor-specific delivery of CD47nb by Fe-Bac-HlpA/CD47nb-BLPs.

Although the Anti-CD47ab in G2 exhibited a comparable therapeutic efficacy compared with Fe-Bac-HlpA/CD47nb-BLPs + AMF in G6, the intraperitoneally injected Anti-CD47ab also elicited hematologic toxicity, including a significant decrease in red blood cell (RBC) number, hemoglobin (HGB) levels and hematocrit (HCT; Fig. 5E, F, S15). Importantly, G6 had no apparent effect on blood parameters, likely owing to the tumor-targeted delivery of CD47nb. To verify this assumption, we examined the amount of Anti-CD47ab or CD47nb on RBCs after a single treatment with Anti-CD47ab or Fe-Bac-HlpA/CD47nb-BLPs + AMF, respectively. A significant amount of Anti-CD47ab was detected on RBCs after intraperitoneal injection of Anti-CD47ab, while CD47nb was undetectable on RBCs after treatment with Fe-Bac-HlpA/CD47nb-BLPs + AMF (Fig. 5G, H), which may be the reason for the hematologic toxicities in G2. Besides, the AMF-Bac treatment in G6 induced no apparent changes in the histopathology of the major organs, indicating that the formulation has a favorable biosafety profile (Supplementary Fig. 16).

Although the mice were treated with the bacteria by colon-specific administration from mouse anus (i.e., enteroclysis), oral administration of the bacteria can be simply achieved by loading bacteria into enteric capsules. The therapeutic efficacy of the oral administration of enteric capsule-loaded Fe-Bac-HlpA/CD47-BLPs (Cap-Fe-Bac-HlpA/CD47-BLPs) was examined. BALB/c mice bearing CT-26-luc colonic orthotopic tumors were randomized into six groups with different treatments (Supplementary Fig. 17A): (I) PBS; (II) CD47nb; (III) Fe-Bac-HlpA/CD47nb-BLPs + AMF; (IV) Cap + AMF; (V) Cap-Fe-Bac-CD47nb-BLPs + AMF; (VI) Cap-Fe-Bac-HlpA/CD47nb-BLPs + AMF. The intraperitoneal injection of CD47nb (group II) exhibited little therapeutic efficacy (Supplementary Fig. 17B, C), probably due to its short half-life in blood^40,41^. Importantly, the oral administration of Cap-loaded engineered bacteria (group VI) had similar tumor inhibition compared with the colon-specific administration of engineered bacteria (group III), as demonstrated by the similar tumor growth (Supplementary Fig. 17B-C), metastatic tumor number in abdomen (Supplementary Fig. 17D), and overall survival time (Supplementary Fig. 17E). Therefore, oral administration of the engineered bacteria could also achieve good therapeutic efficacy. Besides, the therapeutic efficacy of group V was obviously reduced compared with group VI (Supplementary Fig. 17B–E), most likely due to the lack of bacterial HlpA, demonstrating bacterial HlpA could improve the therapeutic efficacy of AMF-Bac in vivo.

Although we have demonstrated that Fe-Bac-HlpA/CD47nb-BLPs + AMF and Anti-CD47ab had comparable therapeutic effects on the small orthotopic tumors, interestingly it was found that Fe-Bac-HlpA/CD47nb-BLPs + AMF had superior therapeutic effects than Anti-CD47ab on the large orthotopic tumors (group G-C vs. group G-B, Supplementary Fig. 18A–E), which also had reduced hematologic toxicity compared with Anti-CD47ab treatment (Supplementary Fig. 18F–H). The superior therapeutic effects on large tumors were possibly due to the much higher intratumoral penetration of AMF-Bac. Motility is the key feature of bacterial therapies that enables intratumoral penetration. Therefore, the bacterial motility and penetration ability were further investigated. Using transwell assays, it was found the AMF-Bac could sense the concentration gradient of nutrient substances, penetrate the Matrigel, and swim from the apical chamber to the basolateral chamber due to the utility of flagella (Supplementary Fig. 19, 20). Transwell assay also demonstrated that bacterial HlpA could increase the tumor-targeting ability of AMF-Bac, showing the in vivo active navigation ability of AMF-Bac in long distances (Supplementary Fig. 21). CLSM images showed that significant amount of Fe-Bac-HlpA/EGFP-BLPs could be observed in deep tumor tissue, 24 h after the administration of Fe-Bac-HlpA/EGFP-BLPs (Supplementary Fig. 22), exhibiting the high penetration ability of AMF-Bac. There were still many live bacteria in tumors at 96 h after administration, suggesting a low clearance rate of bacteria in tumors (Supplementary Fig. 23). In summary, AMF-Bac exhibited excellent motility, active navigation ability, and intratumoral penetration ability, which led to enhanced therapeutic efficacy on large tumors.

### Immune responses induced by AMF-Bac

**Fig. 6:**
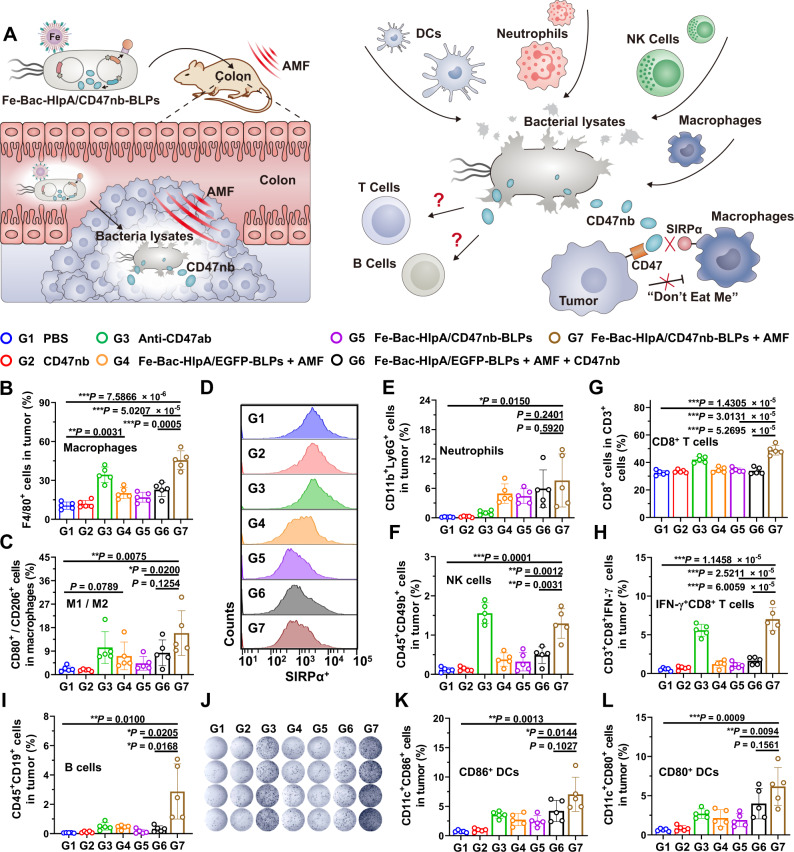
Immune responses induced by AMF-Bac. **A** Illustration of potential immune responses induced by AMF-Bac. AMF induces the lysis of bacteria, and bacterial lysates may recruit and/or activate macrophages, neutrophils, NK cells, and/or DCs. The release of CD47nb blocks the “Don’t eat me” pathway and enhances the phagocytosis of tumor cells by macrophages. Besides innate immune responses, the adaptive immune responses may also be affected. The colons of BALB/c mice were inoculated with CT-26 cells (1 × 10^5^ cells/mouse) on day -7, and mice were treated with the indicated engineered bacteria (1 × 10^8^ CFU) by colon-specific administration on days 0 and 3, followed by AMF treatment (310 kHz and 23.8 kA/m) for 80 min at 24 h after colon administration. The Anti-CD47ab (20 mg/kg) and CD47nb (20 mg/kg) were *i.p*. injected on days 0 and 3 in G3 and G2/G6, respectively. Analysis of the immune responses was performed on day 9. **B** Flow cytometry analysis of the percentage of macrophages (F4/80^+^ cells) in tumors (*n* = 5 mice). **C** The ratio of M1 to M2 macrophages (F4/80^+^CD80^+^ cells vs. F4/80^+^CD206^+^ cells) in tumors (*n* = 5 mice). **D** Flow cytometry analysis of the expression of SIRPα in tumor-infiltrating macrophages (*n* = 5 mice, cf. Supplementary Figure 25B). **E**–**I** Flow cytometry analysis of tumor-infiltrating neutrophils (CD11b^+^Ly6G^+^ cells; **E**), NK cells (CD45^+^CD49b^+^ cells; **F**), CD8^+^ T cells (CD3^+^CD8^+^ cells; **G**), effector T cells (CD3^+^CD8^+^IFN-γ^+^ cells; **H**) and B cells (CD45^+^CD19^+^ cells; **I**) (*n* = 5 mice). **J** IFN-γ secretion by the splenocytes, as determined by ELISPOT assay after re-stimulation with a CT-26-specific antigen peptide (sequence: SPSYVYHQF; *n* = 4 mice). **K**, **L** Flow cytometry analysis of tumor-infiltrating, mature DCs (*n* = 5 mice). The percentages of CD11c^+^CD86^+^ (**K**) or CD11c^+^CD80^+^ (**L**) cells are shown. The data (**B**, **C**, **E**–**I**, **K**, **L**) are shown as the mean ± SD. Statistical analysis was performed by a two-tailed unpaired *t* test. ^*^P < 0.05; ^**^P < 0.01^; ***^P < 0.001. Source data are provided as a Source Data file.

We conducted a panoramic analysis of immune cell status in the tumor microenvironment to investigate the mechanism of the antitumor immune responses induced by AMF-Bac (Fig. 6A). BALB/c mice bearing CT-26 colonic orthotopic xenografts received the following treatments twice, on days 0 and 3: G1, PBS; G2, CD47nb; G3, Anti-CD47ab; G4, Fe-Bac-HlpA/EGFP-BLPs + AMF; G5, Fe-Bac-HlpA/CD47nb-BLPs; G6, Fe-Bac-HlpA/EGFP-BLPs + AMF + CD47nb; G7, Fe-Bac-HlpA/CD47nb-BLPs + AMF. Flow cytometry analysis of the tumor-infiltrating immune cells was performed on day 9.

Compared with the PBS treatment control (G1), treatment with Fe-Bac-HlpA/EGFP-BLPs + AMF (G4) induced an increase in macrophages (Fig. 6B and S24), particularly M1 macrophages (Supplementary Fig. 25A, S26), and an elevation of the ratio of M1 to M2 macrophages (Fig. 6C) in tumors. There was an apparent downregulation of SIRPα in macrophages in G4 group, compared with G1 (Fig. 6D and S25B). Previous studies have shown that lipopolysaccharide exposure led to downregulation of SIRPα on macrophages^42,43^. We hypothesized that the abundant immunogenic adjuvants in bacterial lysis (e.g., flagellin) may lead to the reduced surface expression of SIRPα on tumor-associated macrophages. The changed number/phenotype of macrophages and expression level of SIRPα indicate that the bacterial lysates in tumors in G4 may have enhanced the therapeutic efficacy of CD47nb, since the main anticancer mechanism of CD47nb relies on the CD47-SIRPα signaling pathway to induce phagocytosis of tumor cells^34^. As expected, these changes in tumor-infiltrating macrophages were most significant in G7 (Fig. 6B–D), indicating the synergy of CD47nb and bacterial lysates. In addition, the percentage of neutrophils (Fig. 6E, S27) and natural killer cells (NK cells; Fig. 6F, S28) was markedly increased in tumor tissues in G7, which also may underlie the improved therapeutic effects.

In addition to innate immunity, treatment with Fe-Bac-HlpA/CD47nb-BLPs + AMF in G7 also elicited a robust stimulation of adaptive immunity. Although there was no change in the proportion of CD4^+^ T cells (Supplementary Fig. 25C and S29), the percentages of tumor-infiltrating CD3^+^ T cells (Supplementary Fig. 25D and S30), CD8^+^ T cells (Fig. 6G and S31), IFN-γ^+^CD8^+^ effector T cells (Fig. 6H) and B cells (Fig. 6I and S32) were significantly increased in G7 compared with those in G1, G4, and G6. After re-stimulation with the CT-26-specific antigen peptide, flow cytometry analysis (Supplementary Fig. 25E and S33) and enzyme-linked immunospot (ELISPOT) assay (Fig. 6J and S25F) of splenocytes revealed an increased proportion of antigen-specific T cells in G7 compared with G1, G4, and G6, demonstrating the robust adaptive immune responses induced by the AMF-Bac treatment.

Antigen-presenting cells bridge the innate immunity and adaptive immunity, with DCs the main antigen presentation cells^33^. Flow cytometry analysis showed that tumor-infiltrating DCs were markedly increased upon treatment with Fe-Bac-HlpA/CD47nb-BLPs + AMF (Supplementary Fig. 25G and S34). The percentages of CD80^+^ (Supplementary Fig. 25H, and S35A) and CD86^+^ (Supplementary Fig. 25I and S35B) cells in the DCs were also increased in G7 compared with those in G1, leading to the higher proportions of mature DCs infiltrating the tumors (Fig. 6K, L) in G7 compared with G1 and G4; these DCs may mediate the activation of adaptive immunity.

The treatment of Anti-CD47ab (G3) increased M1 TAMs (Fig. 6C), NK cells (Fig. 6F), and IFN-γ^+^CD8^+^ T cells (Fig. 6H), but the treatment of CD47nb (G2) induced little change of these tumor-infiltrating immune cells due, corresponding to the lack of therapeutic efficacy of systemically injected CD47nb (Supplementary Fig. 17). Besides, the Fc domain endows anti-CD47 antibodies with the effect of antibody-dependent cell-medicated cytotoxicity (ADCC) and antibody-dependent cellular phagocytosis (ADCP). The lack of Fc domain of CD47nb possibly also caused the different immune responses from Anti-CD47ab. For the treatment of Fe-Bac-HlpA/CD47nb-BLPs + AMF, the immune responses triggered by bacterial lysis could overcome the limitation of Fc-lacking CD47nb, generating high antitumor efficacy with reduced side effects.

Fe_3_O_4_ nanoparticles of AMF-Bac possibly also had immunomodulatory effects in the tumor microenvironment. In vitro assays exhibited 4.41% of Fe_3_O_4_ nanoparticles fell from Fe-Bac-HlpA/CD47nb-BLPs after 24 h of proliferation (Supplementary Fig. 36). The Fe_3_O_4_ nanoparticles could possibly be internalized and degraded by endolysosomes of immune cells, and/or by Fenton reaction to release free Fe ions and to facilitate reactive oxygen species production, leading to the polarization and reprogramming of M2 TAMs to M1 phenotype^44^.

### Activation of the type I IFN signaling pathway by AMF-Bac

**Fig. 7:**
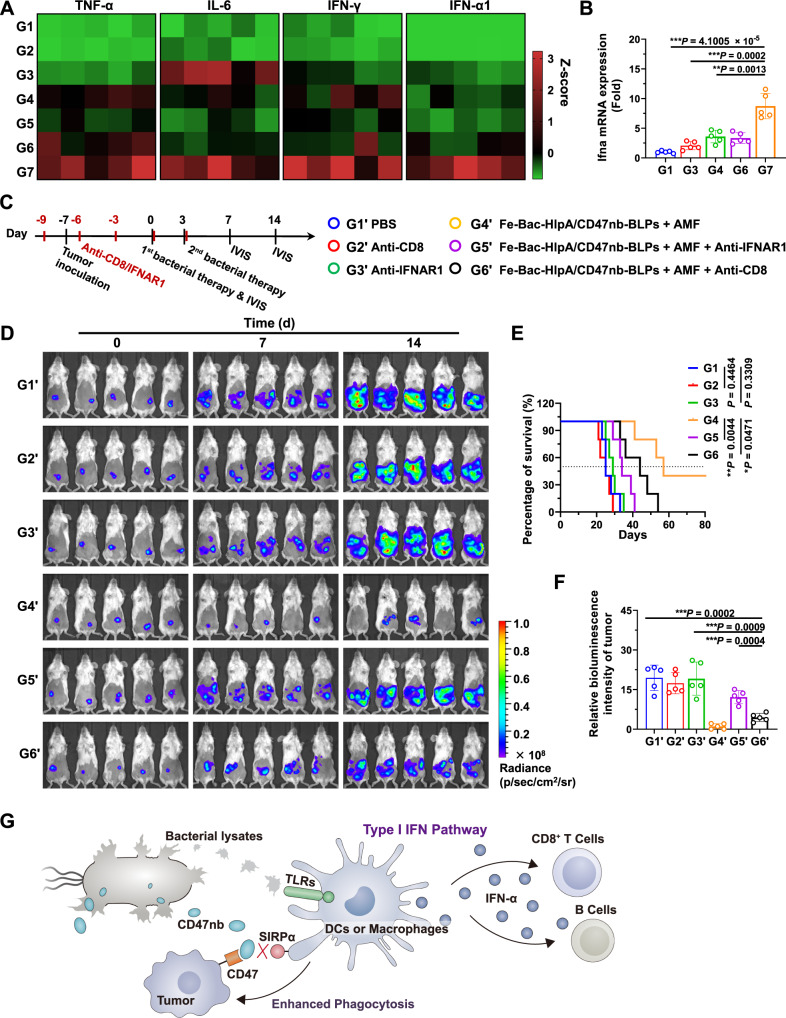
Activation of type I IFN signaling pathway by AMF-Bac. **A** Serum levels of TNF-α, IL-6, IFN-γ, and IFN-α1, as analyzed by ELISA on day 9 in the indicated groups introduced in Fig. 6A (*n* = 5). Cytokine concentration of each sample was normalized to Z-score (i.e., standard score), which was calculated as (*X* – E[*X*])/σ[*X*]. *X*, the cytokine concentration of the individual sample; E[*X*], the average concentration of all samples; σ[*X*], the standard deviation of cytokine concentrations of all samples. **B** *Ifna* mRNA expression levels in tumor-infiltrating DCs, as determined by RT-qPCR on day 9 (*n* = 5 mice). **C** Scheme and grouping of in vivo therapy to evaluate the role of the type I IFN signaling pathway and CD8^+^ T cells. The colons of BALB/c mice were inoculated with CT-26-luc cells on day -7, and the mice were treated with engineered bacteria (1 × 10^8^ CFU) by colon-specific administration on day 0 and day 3, followed by AMF treatment (310 kHz and 23.8 kA/m) for 80 min at 24 h after colon administration. Anti-CD8 (15 mg/kg, clone TIB210) or anti-IFNAR1 (15 mg/kg, clone R46A2) neutralization antibodies were *i.p*. injected on days −9, −6, −3, 0, and 3. **D** Bioluminescence imaging was performed to evaluate tumor growth on day 0, 7, and 14 (*n* = 5). **E** Survival curves of mice from the indicated groups for 80 days (*n* = 5 mice). **F** Semi-quantitative results of bioluminescence intensity in the tumor region on day 14, which were normalized to day 0 (*n* = 5 mice). **G** Schematic illustration of the type I IFN pathway and adaptive immunity activated by AMF-Bac. TLRs, Toll-like receptors. The data (**B**, **E**, **F**) are shown as the mean ± SD. Statistical analysis was performed by a two-tailed unpaired *t* test. Survival significance was analyzed by the log-rank test. ^*^P < 0.05; ^**^P < 0.01^; ***^P < 0.001. Source data are provided as a Source Data file.

We proceeded to explore the underlying mechanism of the stimulation of the adaptive immune response to understand how the engineered bacteria may activate both adaptive and innate immune responses. Treatment with Fe-Bac-HlpA/EGFP-BLPs + AMF (G4 in Fig. 6) and Fe-Bac-HlpA/CD47nb-BLPs + AMF (G7 in Fig. 6) upregulated the plasma concentration of proinflammatory factors, including TNF-α, IL-6, IFN-γ, and IFN-α1 (Fig. 7A). These factors can stimulate, recruit and expand immune cells, which may improve the immunotherapeutic effects of CD47nb. We were particularly interested in the increase in IFN-α1, which is mainly involved in the type I IFN signaling pathway and can promote adaptive immunity. After the treatment with Fe-Bac-HlpA/CD47nb-BLPs + AMF (G7 in Fig. 6), DCs from tumor tissues were sorted, and the mRNA level of *Ifna* (IFN-α1 encoding gene) was found to be markedly upregulated compared with G1, G3 and G4 in Fig. 6 (Fig. 7B). These results indicate that the AMF-Bac treatment significantly activated the type I IFN signaling pathway in the tumor tissue. To determine whether the activation of type I IFN signaling pathway was required for the antitumor effects of AMF-Bac treatment, mice were administered anti-IFNAR1 antibodies for blocking the type I IFN pathway before and during treatment with Fe-Bac-HlpA/CD47nb-BLPs + AMF (Fig. 7C). In addition, we also evaluated the role of CD8^+^ T cells in the antitumor effects of AMF-Bac treatment using anti-CD8 antibodies to deplete the CD8^+^ T cells. As shown in Fig. 7D–F, blockage of the type I IFN pathway (G5’) or depletion of CD8^+^ T cells (G6’) significantly impaired the therapeutic efficacy of AMF-Bac (G4’). Therefore, the type I IFN pathway, as well as CD8^+^ T cell-mediated adaptive immunity, plays a vital role in the antitumor efficacy of AMF-Bac.

Based on the results thus far, we formulated a hypothesis to explain the mechanism by which AMF-Bac promotes adaptive immunity (Fig. 7G): The bacterial lysates stimulate the type I IFN pathway in innate immune cells (e.g., DCs) through the activation of toll-like receptors (TLRs)^45^, while the CD47nb block the “don’t eat me” signaling pathway and increase phagocytosis of tumor cells by macrophages and DCs;^38^ the activation of the type I IFN pathway and increased uptake of tumor antigen ultimately promotes antigen presentation and adaptive immune responses^33,38^.

### Abscopal effects of the immunotherapy of AMF-Bac

**Fig. 8:**
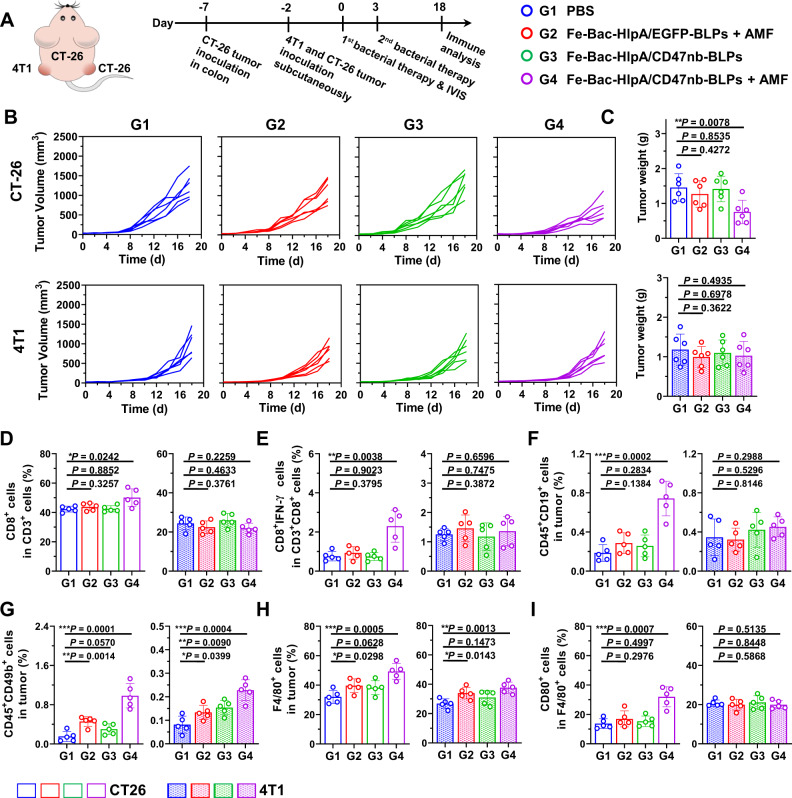
Abscopal effect of the immunotherapy of AMF-Bac. **A** Scheme and groups of in vivo therapy to evaluate the abscopal effect. BALB/c mice were inoculated with CT-26 cells in the colon on day -7, and subcutaneously inoculated with CT-26 and 4T1 cells in the right and left hind limb, respectively, on day -2. The mice were treated with engineered bacteria (1 × 10^8^ CFU) by colon-specific administration on days 0 and 3, followed by AMF treatment (310 kHz and 23.8 kA/m) for 80 min at 24 h after colon administration. Tumors were collected for flow cytometry on day 18. **B** Change in tumor volume of the subcutaneous CT-26 and 4T1 xenografts (n = 5). **C** Tumor weight of the subcutaneous CT-26 and 4T1 xenografts measured on day 18 (*n* = 5 mice). **D**, **E** Flow cytometry analysis of the percentages of tumor-infiltrating CD8^+^ T cells in the total tumor-infiltrating CD3^+^ T cells (**D**), and effector CD8^+^ T cells (CD3^+^CD8^+^IFN-γ^+^ cells) in the total tumor-infiltrating CD3^+^CD8^+^ T cells (**E**) (*n* = 5 mice). **F**, **G** Flow cytometry analysis of the percentages of B cells (CD45^+^CD19^+^ cells; **F**) and NK cells (CD45^+^CD49b^+^ cells; G) in tumors (*n* = 5 mice). **H**, **I** Flow cytometry analysis of the percentage of macrophages (F4/80^+^ cells) in tumors (**H**), and the percentage of M1 macrophages (F4/80^+^CD80^+^ cells) in tumor-infiltrating macrophages (**I**; *n* = 5 mice). The data (**C**, **D**–**I**) are shown as the mean ± SD. Statistical analysis was performed by a two-tailed unpaired *t* test. ^*^P < 0.05; ^**^P < 0.01^; ***^P < 0.001. Source data are provided as a Source Data file.

Since our results demonstrate that AMF-Bac can induce adaptive immune responses, we next examined the abscopal effects of the immune response elicited by AMF-Bac. To this end, we subcutaneously inoculated BALB/c mice bearing CT-26 colonic orthotopic xenografts with CT-26 and 4T1 cells in the right and left hind limb, respectively (Fig. 8A). Colon-specific treatment of orthotopic CT-26 tumors with Fe-Bac-HlpA/CD47nb-BLPs + AMF significantly delayed the growth of subcutaneous CT-26 tumors, but had little influence on 4T1 tumors (Fig. 8B, C). Tumor-infiltrating lymphocytes in the subcutaneous tumors were analyzed by flow cytometry. The amount of tumor-infiltrating CD4^+^ T cells exhibited little change in both subcutaneous CT-26 and 4T1 tumors after Fe-Bac-HlpA/CD47nb-BLPs + AMF treatment (Supplementary Fig. 37A and S38); however, the percentages of CD8^+^ T cells (Fig. 8D and S39), effector CD8^+^ T cells (CD3^+^CD8^+^IFN-γ^+^ cells; Fig. 8E and S40) and B cells (Fig. 8F and S41) significantly increased in the subcutaneous CT-26 tumors in G4 compared with those in G1, none of which were affected in the subcutaneous 4T1 tumors. Neutrophils exhibited little change in either of the subcutaneous tumor types in G4 compared with G1 (Supplementary Fig. 37B and S42). The percentages of NK cells (Fig. 8G and S43) and macrophages (Fig. 8H and S44) both increased in the subcutaneous CT-26 and 4T1 tumors after Fe-Bac-HlpA/CD47nb-BLPs + AMF treatment, but the percentage of M1 macrophages only increased in CT-26 subcutaneous tumors (Fig. 8I and S45), despite the absence of changes in the proportion of M2 macrophages (Fig S37C and S48). Therefore, immunotherapy with AMF-Bac elicited apparent abscopal effects, which were mainly mediated by CD8^+^ T cells and M1 macrophages.

### The constant magnetic field (CMF)-controlled motion of AMF-Bac

The gene expression and drug release behavior of AMF-Bac can be spatiotemporally manipulated in vivo by the AMF. The ultrasound-controlled bacterial gene expression has been reported, and ultrasound-activated therapeutic microbes can successfully turn on in situ to induce a marked suppression of tumor growth^46^. Compared with ultrasound manipulation, one of the advantages of the current method is that AMF-Bac can achieve CMF-controlled motion. The ferromagnetism of Fe_3_O_4_ nanoparticles enable directional movement of AMF-Bac in CMF. With the guidance of CMF, more AMF-Bac could target tumors, therefore enhancing the therapeutic efficacy of AMF-Bac.

**Fig. 9:**
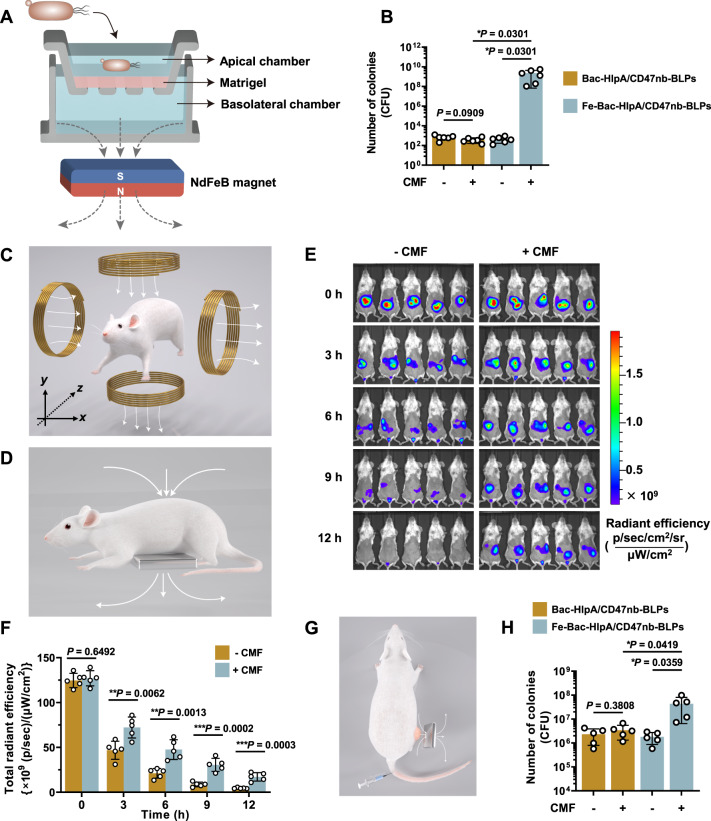
The constant magnetic field (CMF)-controlled motion of AMF-Bac for enhanced tumor targeting. **A** Illustration of transwell assays for analyzing the CMF-controlled motion of AMF-Bac in vitro. A layer of Matrigel was spread on the bottom of the apical chamber. Bac-HlpA/CD47nb-BLPs or Fe-Bac-HlpA/CD47nb-BLPs dispersed in RPMI-1640 medium at density of 1 × 10^8^ CFU mL^−1^ was added into the apical chamber, which was immersed in RPMI-1640 medium of the basolateral chamber. A NdFeB magnet was placed below the basolateral chamber for providing CMF. After incubating at 37 °C for 12 h, the medium of basolateral chamber was sampled for measuring the number of bacteria. **B** The number of live bacteria in the medium of basolateral chamber (*n* = 6 chambers) in panel A, measured by spread plate method. **C** Illustration of controlling AMF-Bac motion in vivo using two-dimension CMF. **D**–**F** Prolonged retention time of AMF-Bac in intestinal tracts by controlling AMF-Bac motion using one-dimension CMF. BALB/c mice were colon-specifically administrated with Cy5.5-labeled Fe-Bac-HlpA/CD47nb-BLPs. A NdFeB magnet was placed below mouse abdomen for providing the one-dimension CMF (**D**). In vivo fluorescence imaging was performed 0–12 h after administration (**E**). The fluorescence intensity of mouse abdomen (*n* = 5 mice) in **E** was semi-quantified (**F**). **G**, **H** Increased bacterial colonization in tumors by controlling AMF-Bac motion to target tumors using one-dimension CMF. BALB/c mice bearing subcutaneous CT-26-luc tumors in the right hind limb were i.v. injected with the Bac-HlpA/CD47nb-BLPs or Fe-Bac-HlpA/CD47nb-BLPs, and the NdFeB magnet was placed close to the tumor for 4 h after injection (**G**) The tumors were collected and ground at 24 h after injection, and the suspension was serially diluted. The number of live bacteria in the tumor (*n* = 5 tumors) was measured by the spread plate method (**H**). The data (**B**, **F**, **H**) are shown as the mean ± SD. Statistical analysis was performed by a two-tailed unpaired *t* test. Survival significance was analyzed by the log-rank test. ^*^P < 0.05; ^**^P < 0.01^; ***^P < 0.001. Source data are provided as a Source Data file.

The CMF-controlled motion of AMF-Bac was investigated in vitro by transwell assays (Fig. 9A, B). A layer of Matrigel was spread on the bottom of the apical chamber. Fe-Bac-HlpA/CD47nb-BLPs was added into the apical chamber. A NdFeB magnet was placed below the basolateral chamber for providing CMF (Fig. 9A), followed by incubating at 37 °C for 12 h. Upon the CMF treatment, the number of live bacteria in basolateral chamber was 4.92 × 10^6^ folds, compared to the control group without CMF treatment (Fig. 9B). The CMF treatment had no significant influence on the bacterial number when Bac-HlpA/CD47nb-BLPs was added into the apical chamber (Fig. 9B). Therefore, CMF could guide AMF-Bac penetrate Matrigel and swim from the apical chamber to the basolateral chamber.

The CMF-controlled motion of AMF-Bac was further investigated in vivo. Theoretically, the three-dimensional motion of Fe_3_O_4_ nanoparticles can be precisely controlled in vivo using two-dimension CMF, since the rotation of CMF in *x*-dimension or *y*-dimension can generate AMF-Bac motion in *z*-dimension (Fig. 9C) as reported^47^. The control of AMF-Bac motion by CMF in mouse intestinal tracts was then examined. BALB/c mice were colon-specifically administrated with Cy5.5-labeled Fe-Bac-HlpA/CD47nb-BLPs. A NdFeB magnet was placed below mouse abdomen for providing the one-dimension CMF (Fig. 9D). In vivo fluorescence imaging showed that the CMF treatment could obviously prolong the retention time of AMF-Bac in intestinal tracts (Fig. 9E). The fluorescence intensity of mouse abdomen upon CMF treatment at 12 h was 3.79 folds compare to the control group without CMF treatment (Fig. 9F, ***P = 0.0003). Therefore, one-dimensional CMF could control AMF-Bac motion in mouse intestinal tracts, leading to the prolong of retention time.

Whether CMF could control the motion of intravenously injected AMF-Bac to target tumors was further examined. The use of attenuated *E. coli* with deficiency in LPS enabled AMF-Bac for intravenous injection. BALB/c mice bearing CT-26-luc subcutaneous xenograft were intravenously injected with AMF-Bac, and the NdFeB magnet was placed close to the tumors for different time intervals (Fig. 9G). 4 h of CMF treatment was enough to significantly increased the bacterial number in tumors, which was 16.97 folds compared to the control group without CMF treatment (Supplementary Fig. 47A). The intratumoral CD47nb concentration upon CMF treatment was also increased to 6.13 times (***P < 0.0001, Supplementary Fig. 47B). When Bac-HlpA/CD47nb-BLPs was injected, the CMF treatment had no significant influence on the intratumoral bacterial number (Fig. 9H) and CD47nb concentration (Supplementary Fig. 47B). Therefore, CMF could control the motion of AMF-Bac, significantly enhancing their tumor-targeting ability.

### The combination of CMF-controlled motion and AMF-controlled gene expression

**Fig. 10:**
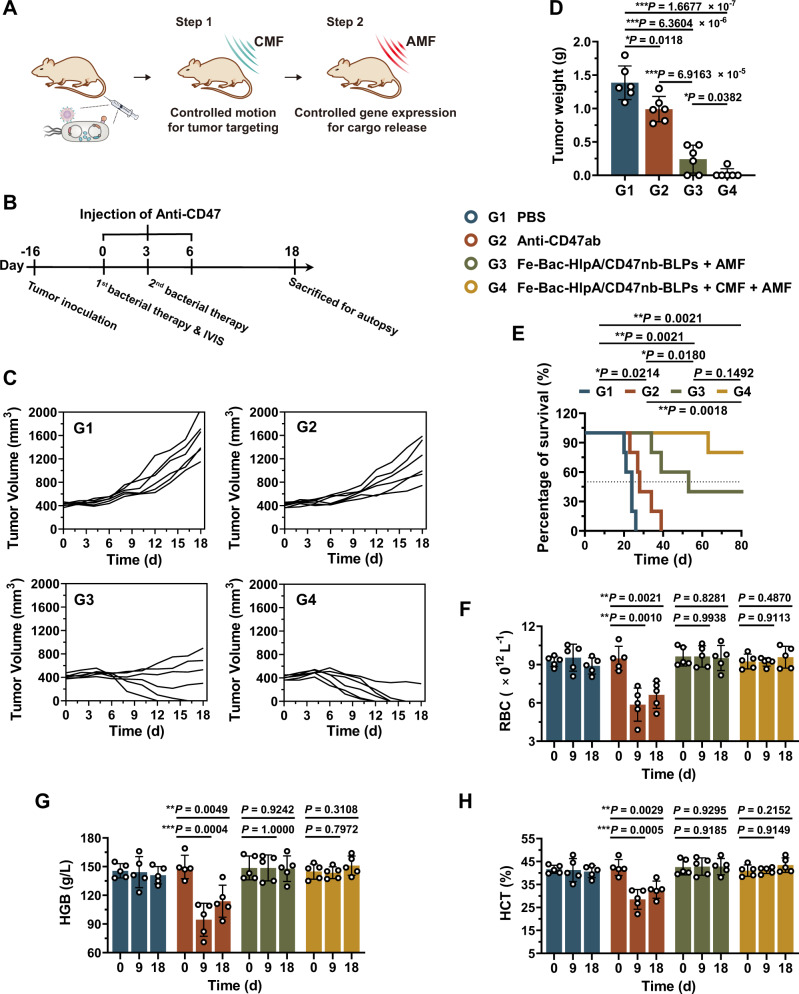
The combination of CMF-controlled motion and AMF-controlled gene expression could achieve excellent therapeutic efficacy with minimal side effects. **A** Illustration of the sequential combination of CMF-controlled motion for tumor targeting and AMF-controlled gene expression for cargo release to achieve excellent therapeutic effects of AMF-Bac. After i.v. administration, AMF-Bac exhibits biased and directional motion towards tumors upon the guidance of CMF (Step 1). Once reaching tumors, AMF-Bac initiates the gene expression of BLPs for bacterial lysis to release the CD47nb cargo (Step 2). **B** Scheme and grouping of in vivo antitumor therapy. BALB/c mice were subcutaneously inoculated with CT-26-luc cells (2 × 10^6^ cells/mouse) in the right hind limb on day −16 to allow the average tumor volume of ~420 mm^3^ at day 0, followed by different treatments. For G2, Anti-CD47ab (20 mg/kg) were *i.p*. injected on days 0, 3, and 6, respectively. For G3, mice were i.v. injected with the Fe-Bac-HlpA/CD47nb-BLPs (5 × 10^6^ CFU) on days 0 and 3, followed by AMF treatment (310 kHz and 23.8 kA/m) for 80 min at 24 h after injection. For G4, mice were i.v. injected with the Fe-Bac-HlpA/CD47nb-BLPs on days 0 and 3, and the NdFeB magnet was placed close to the tumor for 4 h after injection, enabling CMF to guide the bacterial motion swimming towards tumors, followed by the same AMF treatment for 80 min at 24 h after injection. **C** Change in tumor volume of the subcutaneous CT-26-luc (*n* = 6). **D** Tumor weight of the subcutaneous CT-26-luc xenografts measured on day 18 (*n* = 6 mice). **E** Survival curves of mice from the indicated groups for 80 days (n = 5 mice). **F**–**H** The changes in RBC (**E**), HGB (**F**), and HCT (**G**) during therapy (*n* = 5 mice). The data (**D**, **E**–**H**) are shown as the mean ± SD. Statistical analysis was performed by a two-tailed unpaired *t* test. Survival significance was analyzed by the log-rank test. ^*^P < 0.05; ^**^P < 0.01^; ***^P < 0.001. Source data are provided as a Source Data file.

Since the CMF guidance could increase the tumor-targeting of AMF-Bac, the sequential combination of CMF-controlled motion and AMF-controlled gene expression could possibly enhance therapeutic effects of AMF-Bac. Theoretically, AMF-Bac can exhibit biased and directional motion towards tumors upon the guidance of CMF after administration (Step 1, as illustrated in Fig. 10A); once reaching tumors, AMF-Bac can initiate the gene expression of BLPs for bacterial lysis to release the CD47nb cargo (Step 2). The therapeutic efficacy of the sequential combination was then examined in vivo using a subcutaneous tumor model with large tumor volume. BALB/c mice were subcutaneously inoculated with CT-26-luc cells in the right hind limb on day -16 to allow the average tumor volume of ~ 420 mm^3^ at day 0, followed by randomized into 4 groups with different treatments (Fig. 10B): G1, PBS; G2, Anti-CD47ab; G3, Fe-Bac-HlpA/CD47nb-BLPs + AMF; G4, Fe-Bac-HlpA/CD47nb-BLPs + CMF + AMF. For G4, mice were *i.v*. injected with the Fe-Bac-HlpA/CD47nb-BLPs, and the NdFeB magnet was placed close to the tumor for 4 h after injection, enabling CMF to guide the bacterial motion towards tumors, followed by the AMF treatment for 80 min at 24 h after injection. Anti-CD47ab could only slightly delay the tumor growth, and no mouse was free from tumor burdens (Fig. 10C). Compared with Anti-CD47ab (G2), Fe-Bac-HlpA/CD47nb-BLPs + AMF (G3) could highly inhibit tumor growth, and 33.3% of mice were totally free of tumors (Fig. 10C). The tumor inhibition rate of G3 was dramatically higher than G2 (82.5% *vs*. 28.5%, Fig. 10D). The overall survival time of G3 was also significantly longer than that of G2 (*P = 0.0180, Fig. 10E). Therefore, Fe-Bac-HlpA/CD47nb-BLPs + AMF had much better therapeutic efficacy than Anti-CD47ab on the large subcutaneous colonic tumors with the tumor volume of ~ 420 mm^3^.

With the combination of CMF and AMF, Fe-Bac-HlpA/CD47nb-BLPs + CMF + AMF (G4) achieved significantly higher therapeutic efficacy than Fe-Bac-HlpA/CD47nb-BLPs + AMF (G3). The tumor burdens were totally eliminated in 83.3% of the tumor-bearing mice (Fig. 10C), and the average tumor weight of G4 on day 18 decreased by 88.2% compared with G3 (Fig. 10D). Therefore, the combination of CMF and AMF could greatly enhance the therapeutic efficacy of AMF-Bac, which was dramatically higher than Anti-CD47ab. Fe-Bac-HlpA/CD47nb-BLPs + CMF + AMF also exhibited high biosafety profile and had no overt hematologic toxicity, while Anti-CD47ab induced obvious changes of RBC, HGB, and HCT (Fig. 10F–H).

## Discussion

The in vivo manipulation of engineered bacteria can be vitally beneficial in the treatment of disease. From the first generation of natural bacteria-based cancer therapy, to the second generation of genetically engineered bacteria, and further to the third generation of customized nanomaterial-assisted bacteria, the functions of therapeutic bacteria are becoming increasingly sophisticated to afford promising therapeutic efficacy^8^. However, the lack of effective strategies for in vivo manipulation is one of the major obstacles to the clinical use of tumor-homing bacteria^1^. For the native oncolytic strains, *Clostridium* and *Salmonella*, that regress tumors by bacterial cytotoxicity, it is essential to cause these strains to specifically colonize and proliferate in tumors rather than normal tissues to avoid infection-associated toxicity^1,6^. For genetically engineered *Salmonella*, *Listeria,* and *Clostridium*, which can express antitumor cytokines or toxins in tumors, the spatiotemporal control of protein synthesis and secretion is essential to avoid side effects from toxic drugs^1,6^. Therefore, despite their different anticancer mechanisms, precise control systems are crucial for bacteria to be effective therapeutic systems.

Existing approaches for in vivo manipulation of engineered bacteria have been extensively explored and are still under development^20^. Inducible promoters are one of the common strategies to control bacterial gene expression. Hypoxia- and low pH-inducible promoters (e.g., the fumarate and nitrate reduction [FNR] system) have been introduced into bacteria, which can then respond to the hypoxic or acidic microenvironment of solid tumors to achieve spatial control of bacterial activity; however, it is still impossible to achieve temporal control with these systems^1,6^. On the other hand, exogenously applied transcriptional inducers (L-arabinose, acetylsalicylic acid, or tetracyclines) can tightly regulate the corresponding inducible promoters for controlling bacterial colonization or gene expression to achieve temporal control of bacterial activity, while unable to achieve spatial control^1,6^. Radiation-inducible promoters (e.g., RecA) can simultaneously achieve temporal and spatial control, but lead to radiation damage of normal tissues^1,6^. Blue light-inducible promoters (e.g., pDawn) can also affect temporal and spatial control, but the tissue penetration of blue light is shorter than one millimeter^20^. The modification of up-conversion nanomaterials on bacteria can convert the near-infrared light into blue light to control bacteria, but only expands the light penetration depth to several millimeters with limited clinical application^20^. The synchronized lysis circuit exemplifies a methodology for leveraging the tools of synthetic biology to control bacterial colonization. The genetic circuits enable bacteria to sense bacterial population density and release genetically encoded cargo at a threshold population density, effectively limiting bacterial overgrowth and continuously releasing drug cargo^19,42,48^. Focused ultrasound, a form of energy that can be applied noninvasively to specific anatomical sites such as solid tumors, is also an ideal tool for manipulating bacterial gene expression in vivo. The ultrasound-activated therapeutic microbes can successfully turn on in situ and induce a marked suppression of tumor growth^46^.

AMF is also an ideal tool for bacteria manipulation, but a specific method to accomplish this has been lacking. AMF has superior tissue penetration compared with light, and is virtually harmless compared with radiation^49^. AMF also has the advantages of achieving non-invasive, real-time, spatiotemporal and exogenous manipulation, in contrast to hypoxia-/pH-inducible promoters and synchronized lysis circuits. However, the AMF manipulation of cells cannot be achieved though molecular biological techniques due to the lack of AMF-inducible promoters. Magnetic hyperthermia, which is generated from magnetic materials in alternating magnetic fields, has been traditionally applied for hyperthermia therapy of cancer and controlling drug release by thermo-responsive matrices^49^. Herein, we combined magnetic materials and heat-inducible promoters in the AMF-Bac to enable the AMF manipulation of gene expression, describing a facile method for realizing AMF manipulation.

In addition to its application in controlling bacteria, this method is theoretically suitable for AMF manipulation of eukaryotic systems due to the availability of heat-inducible promoters in eukaryotic cells (e.g., the promoter of heat-shock protein HSP70)^50^. The AMF-manipulation of eukaryotic systems has broad application prospects, such as realizing the AMF-inducibility of the chimeric antigen receptor T cells (CAR-T cells) and controlling the activity of CAR-T cells within tumors. AMF manipulation of the activation of engineered T cells may facilitate the design of safer cell therapies with improved on-target off-tumor activity and mitigated side effects. Compared with light-controllable CAR-T cells^51^, AMF manipulation possesses better applicability due to the deep tissue penetration of AMF. Another promising use of AMF manipulation is in the field of optogenetics and magnetogenetics. Optogenetics achieves the light control of neurons via transfecting cells with light-gated ion channels, which has advanced the entire field of neurobiology^52^. However, the current methods around optogenetics are invasive, required fiber optics implantation into the brain. Magnetogenetics can achieve the non-invasive modulation of neurons using magnetic fields, but the method of fusing the iron-binding protein, ferritin, to transient receptor potential (TRP) channels in the neuronal membrane has been called into question for being contrary to physics principles^53^. The method we describe here provides a possible mechanism to achieve AMF manipulation of neurons.

Certain upgrades to the current construction may improve the performance of AMF-Bac. As a model of proof-of-concept, the attenuated *E. coli BL21* strain was adopted as the bacteria chassis for AMF-Bac. The in vivo safety of probiotics (e.g., *E. coli Nissle 1917*) and attenuated strains (e.g., attenuated *Salmonella*) have been confirmed by multiple clinical trials^2^. Changing the design to exploit these genera of bacteria that exhibit high biosafety may make AMF-Bac more suitable for clinical application. The payload of AMF-Bac (CD47nb) may also be replaced by other immunotherapeutic drugs to achieve different purposes of treatment, such as immune checkpoint inhibitors, such as PD-L1 and cytotoxic T-lymphocyte associated protein 4 (CTLA-4). AMF-Bac could achieve the tumor-targeted delivery of these drugs with enhanced therapeutic efficacy and reduced toxicity. The immune responses induced by bacterial lysates may possibly cooperate with these immune checkpoint inhibitors and further enhance their efficacy, since the bacterial lysates can increase immune cell infiltration, convert “cold tumors” into “hot tumors” and activate the type I IFN signaling pathway in tumor-infiltrating DCs, as demonstrated in our results.

In conclusion, we fabricated a prototype bacteria-based micro-biorobot, AMF-Bac, comprising five modules, including active navigation, signal decoding, signal feedback, signal process and signal output. After targeting orthotopic colon tumors, the Fe_3_O_4_ nanoparticles on the modified bacteria equip AMF-Bac to receive and convert magnetic signals into heat signals, which initiate the expression of bacteria lysis proteins, leading to bacteria lysis and the release of the antitumor protein CD47nb, that was pre-expressed and stored within the AMF-Bac. The AMF-Bac also enabled CMF-controlled motion for enhanced tumor targeting and significantly increased therapeutic efficacy. These results not only demonstrate a method for non-invasive, real-time and spatiotemporal AMF manipulation of gene expression of tumor-homing bacteria, but also introduce the concept of modular design into the construction of bacterial systems.

## Methods

### Animals

Our research complies with all relevant ethical regulations. The study protocol was approved by local ethics committees. All animal experiments were approved by the Institutional Animal Care and Use Committee of the National Center for Nanoscience and Technology. The animal study complied with relevant ethical regulations for animal testing and research. Female, 6–7-week-old BALB/c mice were obtained from Vital River Laboratory Animal Technology Co. Ltd (China). Mice were housed in a room with a temperature of 20–22 °C and a humidity of 30–70%. Feed and water were available ad libitum. Artificial light was provided in a 12 h light/12 h dark cycle. The maximal tumor size permitted by the ethics committee is 2000 mm^3^, and the maximal tumor size was not exceeded in this study.

### Materials

APC-anti-mouse CD3 (catalog No. 100235, clone name: 17A2, 1:100), PE-anti-mouse CD4 (catalog No. 100408, clone name: GK1.5, 1:100), FITC-anti-mouse CD8α (catalog No. 100705, clone name: 53-6.7, 1:100), PE/Cy7-anti-mouse IFN-γ (catalog No. 505826, clone name: XMG1.2, 1:100), PE-Cy7-anti-mouse CD45 (catalog No. 103114, clone name: 30-F11, 1: 100), FITC-anti-mouse CD49b (catalog No. 103504, clone name: HMα2, 1: 100), PE-anti-mouse CD11b (catalog No. 101208, clone name: M1/70, 1: 100), APC-anti-mouse GR1 (catalog No. 108412, clone name: RB6-8C5, 1: 100), FITC-anti-mouse Ly-6G (catalog No. 127606, clone name: 1A8, 1: 100), APC-Cy7-anti-mouse F4/80 (catalog No. 123118, clone name: BM8, 1: 100), PE-anti-mouse CD80 (catalog No. 104708, clone name: 16-10A1, 1: 100), PE/Cy7-anti-mouse CD86 (catalog No. 105014, clone name: GL-1, 1: 100), APC-anti-mouse CD19 (catalog No. 152409, clone name: 1D3/CD19, 1: 100), FITC-anti-mouse CD172a (SIRPα) antibody (catalog No. 144005, clone name: P84, 1: 100), APC-anti-mouse/human CD11b (catalog No. 101211, clone name: M1/70, 1: 100) were purchased from BioLegend (USA). APC-anti-mouse CD206 (catalog No. E-AB-F1135UE, clone name: C068C2, 1: 100) was purchased from Elabscience (China). The anti-mouse CD47 antibody (catalog No. BE0270, clone name: MIAP301, 1: 1000 for in vitro use, 20 mg/kg for in vivo use), anti-mouse CD8 antibody (catalog No. BE0061, clone name: 2.43, 15 mg/kg for in vivo use), anti-mouse IFNAR1 antibody (catalog No. BE0241, clone name: MAR1-5A3, 15 mg/kg for in vivo use), rat anti-mouse CSF1 neutralizing antibody (catalog No. BE0204, Clone: 5A1, 100 μg/mouse for in vivo use), rat IgG1 isotype control (catalog No. BE0088, Clone: HRPN, 100 μg/mouse for in vivo use), and anti-FcR antibody (catalog No. BE0307, clone name: 2.4G2, 1.0 µg per 1000000 cells in 100 µL volume) were purchased from BioXcell (USA). Anti-E. coli O + E. coli K antibody (Catalog No. ab31499, 1:100), Alexa Fluor® 647-conjuaged anti-6X His-tag® antibody (Catalog No. ab237337, clone name: EPR20547, 1: 100), anti-syndecan-1 antibody (Catalog No. ab128936, clone: EPR6454, 1: 1000) were purchased from Abcam (UK). The His-tag Protein ELISA kit (catalog No. ab285248) and mouse IFN-α1 ELISA kit (catalog No. ab252352) were purchased from Abcam (UK). Mouse IL-6 ELISA kit (catalog No. EMC004.48) was purchased from NeoBioscience Technology Co., Ltd. (China). Mouse TNF-α ELISA kit (catalog No. SEKM-0034-96) was purchased from Solarbio (China). Mouse IFN-γ ELISA Kit (catalog No. CZM10-96) was purchased from Beijing CHENG ZHI KE WEI Biotechnology Co., Ltd. (China). Recombinant mouse CD47 protein (catalog No. 57231-MNAH) was purchased from Sino Biological Inc. (China). Recombinant human CD47 protein (Catalog No. CG18) was purchased from Novoprotein (China). Alexa Fluor 555-labeled phalloidin were purchased from Absin (China). BHQ-3 carboxylic acid was purchased from LGC Biosearch Technologies (USA). 1,2-dipalmitoyl-rac-glycero-3-phosphocholine (DPPC), 1,2-dioctadecanoyl-sn-glycero-3-phophocholine (DSPC), DSPE-PEG2000-DBCO and DSPE-PEG2000-Cy5.5 were purchased from Xi’an Ruixi Biological Technology Co., Ltd (China). Tetra-acetylated N-azidoacetylgalactosamine (Ac_4_GalNAz) was purchased from Jena Bioscience GmbH (Germany). Enteric capsules and intragastric administration devices for mouse were purchased from Yuyan Instruments (China). Matrigel (catalog No. 354234) was purchased from Corning (USA). Deionized water was acquired with a Millipore Milli-Q Gradient System (USA).

### Plasmid construction and bacteria engineering

The pACYCDuet-1 plasmid enables co-expression of two target genes. The genes encoding CD47nb and ClyA-HlpA (or INP-HlpA) were cloned into pACYCDuet-1 for co-expression of CD47nb and ClyA-HlpA (or INP-HlpA), and referred to as pACYC-ClyA-HlpA-CD47nb or pACYC-InN-HlpA-CD47nb. The genes encoding CD47nb and EGFP were also cloned into pACYCDuet-1 for co-expression of CD47 and EGFP, and referred to as pACYC-ClyA-HlpA-EGFP. The plasmid pBV220 enables the heat-induced expression of target genes at 42 °C. The gene encoding bacteria lysis proteins (protein E of phiX174 phage, Gene ID: 2546400) was cloned into pBV220, and referred to as pBV220-BLPs. The attenuated *Escherichia coli* (ClearColi BL21 (DE3), purchased from ZOMANBIO Corp., China) with deficiency in LPS was simultaneously transformed with modified pACYCDuet-1 and pBV220 by electroporation, and the desired colonies were selected using kanamycin (50 μg/mL) and ampicillin (50 μg/mL). For the co-expression of CD47nb and HlpA, and the introduction of -N_3_ moieties onto the bacterial surface via metabolic oligosaccharide engineering, transformed bacteria were grown to mid-log phase in LB medium containing kanamycin, ampicillin, and 50 μM Ac_4_GalNAz (Jena Bioscience GmbH, Germany) at 37 °C with shaking (180 rpm), and then induced with 1 mM isopropyl β-d-1-thiogalactopyranoside (IPTG) overnight at 30 °C. CD47nb was purified by Ni-NTA and Superdex 75 column chromatography^40^. The CD47nb bound immobilized mouse CD47 with a *K*_D_ of ~12 pM characterized by surface plasmon resonance^40^. The amino-acid sequence of the CD47nb:

QVQLVESGGGLVEPGGSLRLSCAASGIIFKINDMGWYRQAPGKRREWVAASTGGDEAIYRDSVKDRFTISRDAKNSVFLQMNSLKPEDTAVYYCTAVISTDRDGTEWRRYWGQGTQVTVSSGG

### Assembly of bacteria with Fe_3_O_4_ NPs

Oleic acid-stabilized Fe_3_O_4_ NPs were synthesized by the hydrothermal method^54^. DBCO-modified Fe_3_O_4_ NPs were fabricated by the filming-rehydration method. Briefly, a 0.5 mg optimized lipid formulation composed of C18-ELP3: DPPC: cholesterol: DSPE-PEG2000-DBCO: BHQ3: Cy5.5 (0.61: 55: 15: 2: 1.5: 0.5, molar ratio) and 0.5 mg oleic acid-stabilized Fe_3_O_4_ NPs was dissolved in chloroform, and dried into a thin film using a rotary evaporator, followed by desiccation overnight under vacuum to remove any traces of chloroform. The film was hydrated in PBS (0.01 M, pH = 7.4) and treated with five repeated freeze-thaw cycles. Uniform hydrophilic Fe_3_O_4_ NPs were obtained by extrusion through a polycarbonate filter with a pore size of 50 nm (Whatman, USA). Centrifugation at 10,000 × *g* for 10 min was performed to remove free lipids and free Cy5.5 that were not encapsulated into the Fe_3_O_4_ NPs. 0.2 mg purified Fe_3_O_4_ NPs were incubated with 5 × 10^8^ bacteria in PBS at 4 °C overnight to allow the conjugation of Fe_3_O_4_ NPs with bacteria through the click chemistry between -DBCO and -N_3_ moieties. The amount of Fe_3_O_4_ NPs modified on bacteria was measured using Fe concentration (Ferrozine colorimetry) assay kit (catalog NO. G1212F, Geruisi-bio, China) after acidolysis.

### Cell culture

CT-26, MC-38, 4T1, HCT-116, and MCF-7 cell lines were originally obtained from the American Type Culture Collection (ATCC). All the cell lines were not commonly misidentified lines. The absence of mycoplasma contamination was confirmed using Quick Cell Mycoplasma Assay kit (catalog NO. AC16L061, Life-iLab, China). CT-26, MC-38, and 4T1 cells were cultured in RPMI-1640 medium supplemented with 10% fetal bovine serum (FBS; Gibco, USA). HCT-116 cells were cultured in IMDM (Iscove’s Modified Dulbecco’s Medium) supplemented with 10% FBS. MCF-7 cells were cultured in MEM-EBSS (MEM Eagles with Earle’s Balanced Salts) supplemented with 10% FBS. The cells were cultured with 100 U mL^−1^ penicillin and 100 μg mL^−1^ streptomycin at 37 °C in a humidified atmosphere of 5 vol% CO_2_. Luciferase-expressing CT-26 (CT-26-luc) cells were obtained by stably transfecting CT-26 with the encoding gene of luciferase using FectinMore^TM^ Transfection Reagent (catalog NO. CM001) purchased from Chamot Biotechnology Co., Ltd. (China).

### Magnetothermal treatment in vitro and in vivo

The magnetothermal effect of AMF-Bac was measured by placing the solution in the center of an induction coil to receive an alternating magnetic field (310 kHz, 14.6 kA/m) generated by an induction heating system (MSI Automation, USA). The temperature of the solution was measured with a fiber optic thermometer. For in vivo treatment with AMF, female mice bearing CT-26 colonic orthotopic xenografts were placed in the center of the induction coil to receive the alternating magnetic field (310 kHz, 23.8 kA/m) for 80 min. The intratumoral temperature was measured by an inserted fiber optic thermometer. Whole-body infrared imaging of mice with exposed colonic tumors was performed using an infrared thermal camera (Fluke Ti27, USA) during AMF treatment.

### AMF treatment-induced bacterial lysis, the release, and affinity of CD47nb cargo

The lysis of engineered bacteria induced by AMF treatment was examined in vivo (cf. Fig. 4F, G). Mice bearing CT-26-luc colonic orthotopic xenografts were treated with Fe-Bac-BLPs or Fe-Bac-HlpA/EGFP-BLPs (1 × 10^8^ CFU) by colon-specific administration. After 24 h, the mice received AMF treatment (310 kHz and 23.8 kA/m) for 80 min. Next, the tumors were collected and ground, and the suspension was serially diluted. The number of live bacteria in the tumor was measured by the spread plate method.

The release of CD47nb from Fe-Bac-HlpA/CD47nb-BLPs after AMF treatment (310 kHz and 23.8 kA/m) for the indicated times (0–160 min) was examined by dot blotting (cf. Fig. 4J). The CD47 protein was adsorbed onto a nitrocellulose membrane, followed by the addition of bacterial lysates. The CD47nb binding to the CD47 protein was detected using a rabbit anti-Myc tag antibody (catalog no. bs-0842R, 1: 1000; Bioss USA).

The affinity of the released CD47nb to the CD47 protein was verified by dot blotting (cf. Fig. 4K). The CD47 protein was adsorbed onto a nitrocellulose membrane, followed by the addition of bacteria lysates of Fe-Bac-HlpA/CD47nb-BLPs after AMF treatment (310 kHz and 23.8 kA/m) for 80 min. The CD47nb binding to the CD47 protein was detected using an anti-Myc tag antibody. Excess anti-CD47 antibodies were used to competitively block the binding of CD47nb to CD47 protein. IgG was a negative control (for the anti-CD47 antibodies).

The affinity of released CD47nb to CD47 in CT-26 cells was examined by competition binding assay using the anti-CD47 antibody (cf. Fig. 4L). Bacterial lysate of Fe-Bac-HlpA/EGFP-BLPs or Fe-Bac-HlpA/CD47nb-BLPs was obtained after AMF treatment (310 kHz and 23.8 kA/m) for 80 min. CT-26 cells were co-incubated with a Cy5.5-labeled anti-CD47 antibody (20 ng/mL) and a series of three-fold dilutions of the obtained bacterial lysate (8.47 × 10^−5 ^mg/mL–5.00 × 10^0 ^mg/mL). After a 2 h incubation at 4 °C, the fluorescence intensity of unbound Cy5.5-labeled anti-CD47 antibody in the supernatant was measured, and the binding rates of anti-CD47 antibody to the CT-26 cells were calculated.

### Tumor growth and treatments

For the establishment of CT-26 colonic orthotopic xenografts, the colons of female BALB/c mice (6-7 weeks old) were inoculated with CT-26 cells (1 × 10^5^ cells/mouse for establishing small tumor models and 2 × 10^6^ cells/mouse for establishing large tumor models) dispersed in Matrigel. Mice were treated with the indicated engineered bacteria (1 × 10^8^ CFU) by colon-specific administration at 24 h prior to AMF treatment. Tumor growth was monitored by bioluminescence imaging of luciferase in CT-26-luc cells. An IVIS® Spectrum In vivo Imaging System (PerkinElmer, USA) was used to perform the bioluminescence imaging, which was initiated 15 min before i.p. injection with 150 mg/kg D-luciferin potassium salt. The luminescence intensities of the regions-of-interest (ROI) of each mouse were quantified using Living Image software (version number, 4.3.1.16427; PerkinElmer, USA). For the establishment of subcutaneous xenografts, female BALB/c mice were subcutaneously inoculated with CT-26 cells or 4T1 cells (1 × 10^6^ cells/mouse) in right or left hind limb. Tumor volume was measured and calculated as *V* = (L × W^2^)/2, where *L* and *W* are tumor length and width, respectively.

### Tumor digestion, flow cytometric sorting, and analysis

After in vivo therapy on female mice, tumor tissues were excised and digested with 1 mg/ml collagenase IV (ThermoFisher, USA) and 100 mg/ml DNase I (ThermoFisher, USA). Digestion was performed at 37 °C with 5% CO_2_ for 30 min. A 70-μm cell filter was used to prepare single-cell suspensions, and cells were washed with PBS containing 2% FBS. For flow cytometric sorting and analysis, the digested cells were blocked with anti-FcR antibody (catalog No. BE0307, clone name: 2.4G2, BioXcell) and then stained with fluorescence-labeled antibodies against protein markers of different immune cells for 30 min. The dilution of antibodies was according to the manufacturer’s instructions. After washing with 1640 medium containing 2% FBS, the cells were analyzed on a Novocyte^TM^ Flow Cytometer (ACEA) or sorted on a FACSAria II Cell Sorter (BD).

### Measurement of IFN-γ-secreting CD8^+^ T cells of splenocytes

On day 9 of the in vivo therapy protocol, splenocytes were isolated from mouse spleens for intracellular IFN-γ flow cytometry analysis and ELISPOT assay. For flow cytometry analysis, splenocytes were co-cultured with a CT-26-specific antigen peptide (AH1 [6-14], sequence: SPSYVYHQF) overnight. Splenocytes treated with ionomycin (Abmole, USA) were used as positive controls. 5 h before collection, monensin (TargetMol, USA) was added to the splenocytes. The cells were collected for labeling with anti-CD3 and anti-CD8 antibodies, followed by fixation and permeation in fixation and permeation buffer (BioLegend, USA). The cells were further stained with an anti-IFN-γ antibody before analysis on a flow cytometer. The ELISPOT assay was performed using a mouse IFN-γ pre-coated ELISPOT kit (Dakewe Biotech Co., Ltd., China). Briefly, splenocytes were seeded in a 96-well plate (10^5^ cells/well) pre-coated with an anti-mouse IFN-γ antibody. After incubation overnight with a CT-26 specific antigen peptide, the secreted and captured IFN-γ was quantified according to manufacturer’s instructions.

### Quantitative real-time RT-PCR (qRT-PCR)

Total RNA from in vivo DCs sorted from tumors in female mice was isolated using TriZol reagent (catalog No. R1000, LABLEAD Inc., China), and reverse-transcribed with a Hifair® II 1st Strand cDNA Synthesis Kit (catalog No. 11119; Yeasen, Shanghai, China). Real-time RT-PCR was performed using SYBR Green Real-time PCR Master Mix (catalog NO. KGA1339, KeyGEN, China). The data were normalized to the levels of β-actin, and the relative levels of *Ifna* and *Ifnb* were calculated using the 2^−ΔΔCt^ method.

Primer sequences:

*Infa1* (Forward): 5’-TCCCCTGACCCAGGAAGATGCC-3’;

*Infa1* (Reverse): 5’-ATTGGCAGAGGAAGACAGGGCT-3’;

*Infb* (Forward): 5’-TGAACTCCACCAGCAGACA-3’;

*Infb* (Reverse): 5’-ACCACCATCCAGGCGTAG-3’;

β-Actin (Forward): 5’CGCGAGAAGATGACCCAGATC’;

β-Actin (Reverse): 5’-CATGAGGTAGTCAGTCAGGTCCC-3’.

### Transwell assay

Transwell assays were employed to analyze the propelling ability of AMF-Bac. A layer of Matrigel was spread on the bottom of the apical chamber of the transwell device. Briefly, the standard Matrigel was diluted in the coating buffer (0.01 M Tris, 0.7% NaCl, pH 8.0) at final concentration of 300 μg/mL, and 100 μL diluted Matrigel was added to the apical chamber of the 24-well transwell device (BioCoat, USA, catalog No. 354578), followed by the gelatinization at 37 °C for 2 h. Different solutions of 700 μL were added into the basolateral chamber including PBS, RPMI-1640 medium, FBS, and tumor interstitial perfusing fluid (TIPF) dissolved in PBS. 200 μL of motile, static, or cryo-treated Fe-Bac-HlpA/CD47nb-BLPs dispersed in PBS at density of 1 × 10^8^ CFU mL^−1^ was added into the apical chamber. The assembled transwell devices were incubated at 37 °C for 12 h, and the solution of basolateral chamber was sampled for measuring the number of bacteria. The TIPF was obtained from CT-26 xenograft, which was excised from tumor-bearing mice, dispersed in PBS, homogenized into single cells by tissue homogenizer, and centrifuged to remove cells and tissue debris. The supernatant after centrifugation was obtained as TIPF, and sterilized with 0.22 μm filters for further use. Static Fe-Bac-HlpA/CD47nb-BLPs was prepared by removing the flagella from the bacteria Bac-HlpA/CD47nb-BLPs using 0.5 M acetic acid, followed by the same procedure for the conjugation of Fe_3_O_4_ nanoparticles^40,55^. Cryo-treated Fe-Bac-HlpA/CD47nb-BLPs with the maintained structure were prepared by a cryogenic treatment in which the hybrid bacteria were immersed in liquid nitrogen for one minute^56^. The bacterial number was measured by spread plate method. Briefly, 50 μL of the solution in basolateral chambers was sampled, serially diluted, and seeded onto a serial of agar plates with 25 μg/mL chloramphenicol. The total number of bacterial colonies on plates was counted to calculate the number of CFU of the solution in basolateral chamber.

To analyzing active navigation ability of AMF-Bac in long distance, a layer of Matrigel was spread on the bottom of the apical chamber of the transwell device. Sterile confocal glass slides of 5 mm × 5 mm coated with polylysine (Sigma-Aldrich, USA) were placed on the bottom of the basolateral chamber. 5 × 10^4^ CT-26 cells dispersed in 700 μL of RPMI-1640 medium were added into the basolateral chamber, followed by the incubation at 37 °C overnight for allowing tumor cells to adhere slides. 200 μL of Fe-Bac-EGFP-BLPs or Fe-Bac-HlpA/CD47nb-BLPs dispersed in PBS solution at density of 1 × 10^8^ CFU mL^−1^ was added into the apical chamber. The assembled transwell devices were incubated at 37 °C for 12 h. Grass slides were collected for visualization of CLSM.

### Statistical analysis

Results are expressed as the mean ± standard deviation (SD). The sample size for analysis was as annotated in figure legends. At least three independent experiments were performed for each in vitro study (Figs. 7B, 9B, Supplementary Fig. 9, 11, 12C, 19–21). At least five mice were included for in vivo antitumor experiments (Fig. 8A–I, 10A–H, Supplementary Fig. 13A–D, 17A–E, 18A–H). Statistical analysis was carried out using SPSS version 19.0. The differences among two groups were assessed by two-tailed and unpaired *t* test. Two-sided log-rank tests were used to analyze Kaplan–Meier curves. GraphPad Prism 8.3.0.538, NovoExpress 1.4.1.1901, BD Accuri C6 1.0.264.21, FlowJo 10.0.0.0, and ImageJ 1.8.0. were used to analyze the acquired data. *P < 0.05, **P < 0.01, and ***P < 0.001 were considered significant. The details of statistical analysis for figures and Supplementary figures are included in the source data files.

### Reporting summary

Further information on research design is available in the Nature Portfolio Reporting Summary linked to this article.

## Supplementary information

Supplementary Information

Reporting Summary

## Data Availability

The authors declare that all the data supporting the results of this study are available within the Article, Supplementary Information, or Source Data file. The gating strategy for flow cytometry experiments can be found in Supplementary Figs. 48–53. Source data are provided in this paper.

## Source data

Source Data
