# Author Correction: Modular-designed engineered bacteria for precision tumor immunotherapy via spatiotemporal manipulation by magnetic field

**DOI:** 10.1038/s41467-023-39906-3

**Published:** 2023-07-10

**Authors:** Xiaotu Ma, Xiaolong Liang, Yao Li, Qingqing Feng, Keman Cheng, Nana Ma, Fei Zhu, Xinjing Guo, Yale Yue, Guangna Liu, Tianjiao Zhang, Jie Liang, Lei Ren, Xiao Zhao, Guangjun Nie

**Affiliations:** 1 CAS Key Laboratory for Biomedical Effects of Nanomaterials and Nanosafety & CAS Center for Excellence in Nanoscience, National Center for Nanoscience and Technology, Beijing, 100190 China; 2 Department of Ultrasound, Peking University Third Hospital, Beijing, 100191 China; 3 IGDB-NCNST Joint Research Center, Institute of Genetics and Developmental Biology, Chinese Academy of Sciences, Beijing, 100101 China; 4 The Higher Educational Key Laboratory of Biomedical Engineering of Fujian Province, Research Center of Biomedical Engineering of Xiamen, Department of Biomaterials, College of Materials, Xiamen University, Xiamen, Fujian 361005 China; 5 Center of Materials Science and Optoelectronics Engineering, University of Chinese Academy of Sciences, Beijing, 100049 China; 6 The GBA National Institute for Nanotechnology Innovation, Guangdong, 510700 China

**Keywords:** Cancer immunotherapy, Bacterial synthetic biology, Nanotechnology in cancer

Correction to: *Nature Communications* 10.1038/s41467-023-37225-1, published online 23 March 2023

The original version of this Article contained an error in Figure 3G. During the assembly of the figure, the mouse images (lower and upper panel) for G5 were also inadvertently assigned to G2. The correct images for G2 have now been provided and Figure 3 has been replaced in both the PDF and HTML versions of the Article. Please note that the correct images (raw data) for G2 were already available in the original Source Data file.

