# Supplementary material for: Modular-designed engineered bacteria for precision tumor immunotherapy via spatiotemporal manipulation by magnetic field
Source: Nat Commun. 2023 Mar 23;14:1606. doi: 10.1038/s41467-023-37225-1 (PMC10036336; doi:10.1038/s41467-023-37225-1)
Supplement: Supplementary file 1 — Supplementary Information [file 41467_2023_37225_MOESM1_ESM.docx]

**Supplementary information**

**Modular-designed engineered bacteria** **for precision tumor immunotherapy via** **spatiotemporal manipulation by magnetic field**

Xiaotu Ma^1,2,3#^, Xiaolong Liang^2#^, Yao Li^1,4#^, Qingqing Feng^1^, Keman Cheng^1^, Nana Ma^1^, Fei Zhu^1^, Xinjing Guo^1^, Yale Yue^1^, Guangna Liu^1^, Tianjiao Zhang^1^, Jie Liang^1^, Lei Ren^4^, Xiao Zhao^1,3,5*^, Guangjun Nie^1,5,6*^

^1^ CAS Key Laboratory for Biomedical Effects of Nanomaterials and Nanosafety & CAS Center for Excellence in Nanoscience, National Center for Nanoscience and Technology, Beijing 100190, China

^2^ Department of Ultrasound, Peking University Third Hospital, Beijing 100191, China

^3^ IGDB-NCNST Joint Research Center, Institute of Genetics and Developmental Biology, Chinese Academy of Sciences, Beijing, 100101, China

^4^ The Higher Educational Key Laboratory of Biomedical Engineering of Fujian Province, Research Center of Biomedical Engineering of Xiamen, Department of Biomaterials, College of Materials, Xiamen University, Xiamen, Fujian 361005, China

^5^ Center of Materials Science and Optoelectronics Engineering, University of Chinese Academy of Sciences, Beijing 100049, China

^6^ The GBA National Institute for Nanotechnology Innovation, Guangdong 510700, China

^#^ These authors contributed equally.

^*^Corresponding authors.

Guangjun Nie: niegj@nanoctr.cn

Xiao Zhao: zhaox@nanoctr.cn


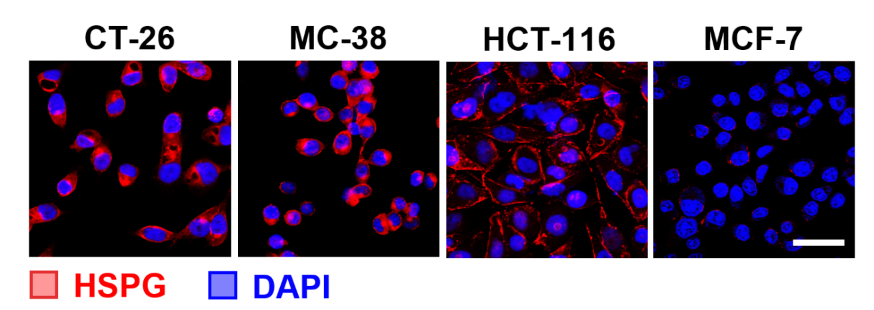


**Supplementary Figure 1.** HSPG expression on the plasma membrane of the indicated cell lines, as observed by CLSM. HSPG was stained with anti-syndecan-1 antibody (red), while cell nuclei were stained with DAPI (blue). These experiments were repeated three times independently with similar results. Scale bar, 80 μm.


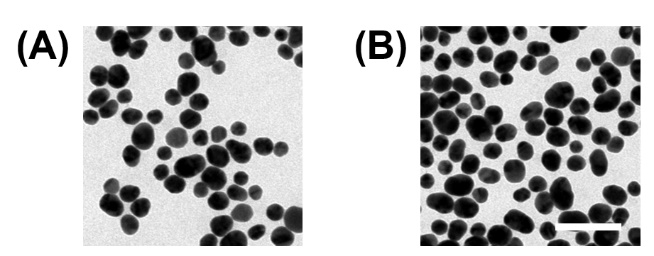


**Supplementary Figure 2.** TEM images of Fe_3_O_4_-COOH (A) and Fe_3_O_4_-DBCO (B). The two types of nanocomposites have a uniform size, with a solid diameter of ~30 nm. Each experiment (A-B) was repeated three times independently with similar results. Scale bar, 100 nm. Source data are provided as a Source Data file.


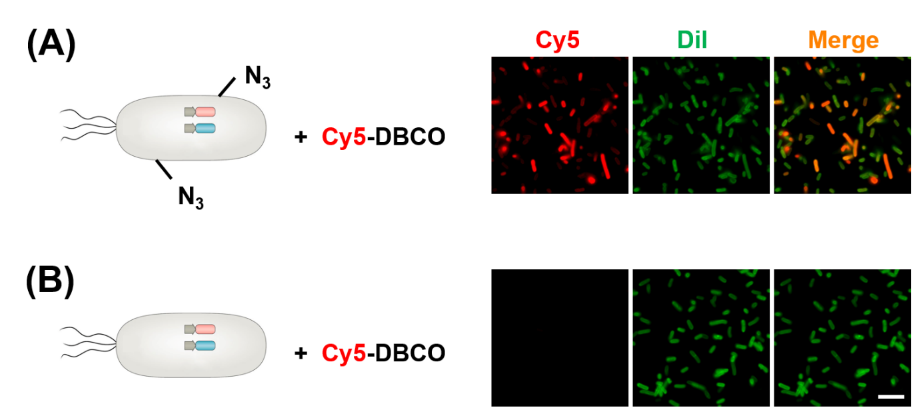


**Supplementary Figure 3.** Introduction of -N_3_ moieties onto the bacteria surface via metabolic oligosaccharide engineering. The bacteria were cultured with a non-natural form of galactosamine (Ac_4_GalNAc) at 37°C with shaking (180 rpm) to introduce -N_3_ moieties onto the bacterial surface. To examine the existence of -N_3_ moieties, Cy5-DBCO then reacts with the N_3_-modified bacteria through the click chemistry reaction between -N_3_ and -DBCO. The successful introduction of -N_3_ moieties is demonstrated by the Cy5 fluorescence (red) of bacteria observed under CLSM. **(A)** N_3_-modified bacteria. **(B)** Bacteria without -N_3_. Bacterial membranes were tracked by DiI staining (green). Each experiment (A-B) was repeated three times independently with similar results. Scale bar, 10 μm.


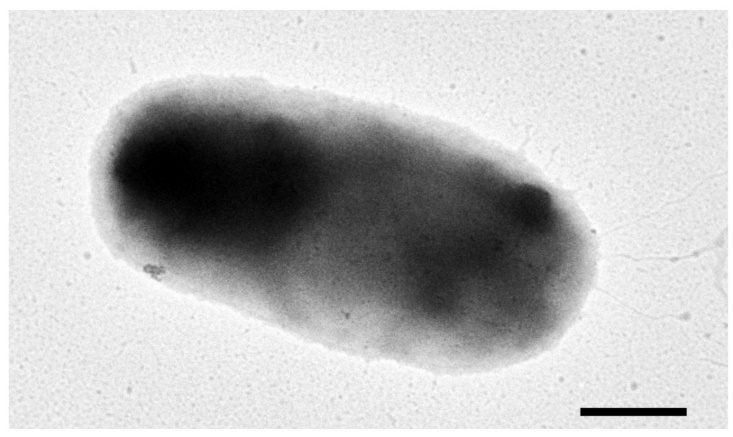


**Supplementary Figure 4.** TEM image showing the decoration efficiency of DBCO-Fe_3_O_4_ nanoparticles on non-N_3_ bacteria. 0.2 mg DBCO-Fe_3_O_4_ nanoparticles were incubated with 5 × 10^8^ Bac-HlpA/EGFP in PBS at 4°C overnight. No DBCO-Fe_3_O_4_ was found on the Bac-HlpA/EGFP without -N_3_ moieties. This experiment was repeated three times independently with similar results. Scale bar, 500 nm. Source data are provided as a Source Data file.


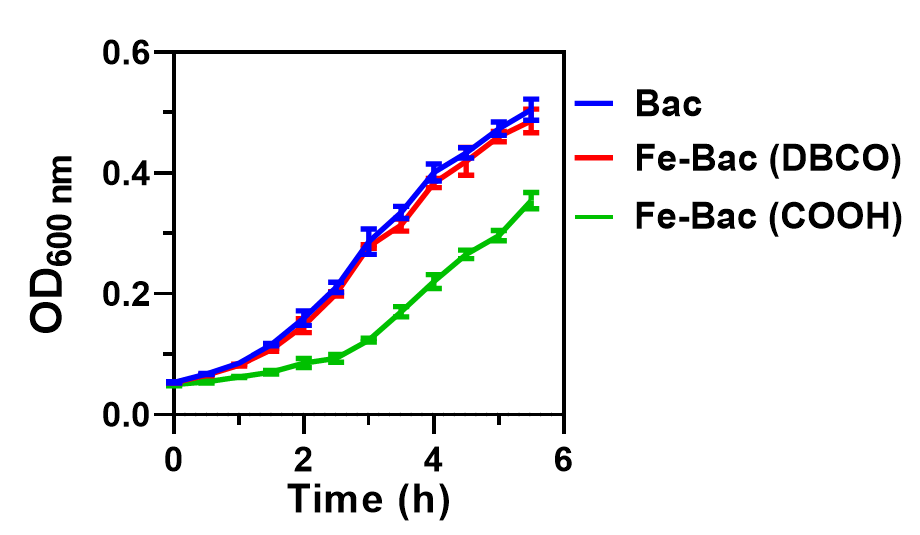


**Supplementary Figure 5.** Growth curves of bacteria (Bac), Fe_3_O_4_-DBCO-modified bacteria (Fe-Bac (DBCO)) and Fe_3_O_4_-COOH-modified bacteria (Fe-Bac (COOH)). The data are shown as the mean ± SD (n = 3 independent experiments). Source data are provided as a Source Data file.


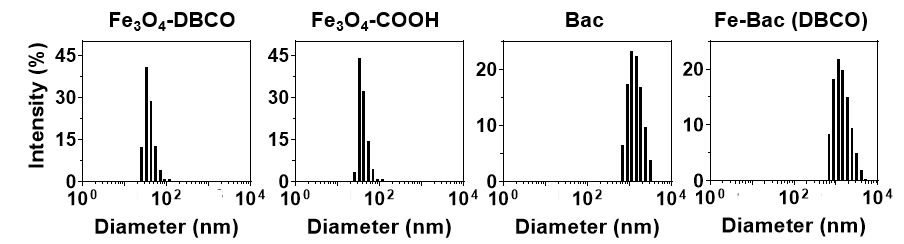


**Supplementary Figure 6.** Hydrodynamic diameter distributions of Fe_3_O_4_-DBCO, Fe_3_O_4_-COOH, Bac and Fe-Bac (DBCO), as detected using dynamic light scattering (DLS). Each experiment was repeated three times independently with similar results. Source data are provided as a Source Data file.


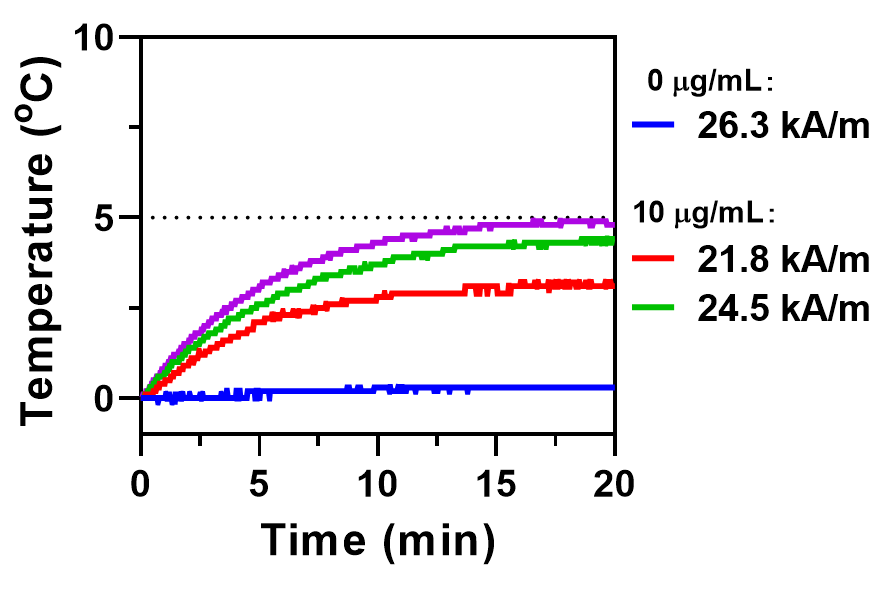


**Supplementary Figure 7.** Temperature elevation of Fe-Bac-HlpA/EGFP suspensions (10 μg/mL Fe_3_O_4_) upon AMF treatment of the indicated intensities (with a fixed frequency of 310 kHz and a range of magnetic field intensity from 21.8 to 26.3 kA/m). The solution with 0 mg/mL engineered bacteria was used as blank control. Each experiment was repeated three times independently with similar results. Source data are provided as a Source Data file.


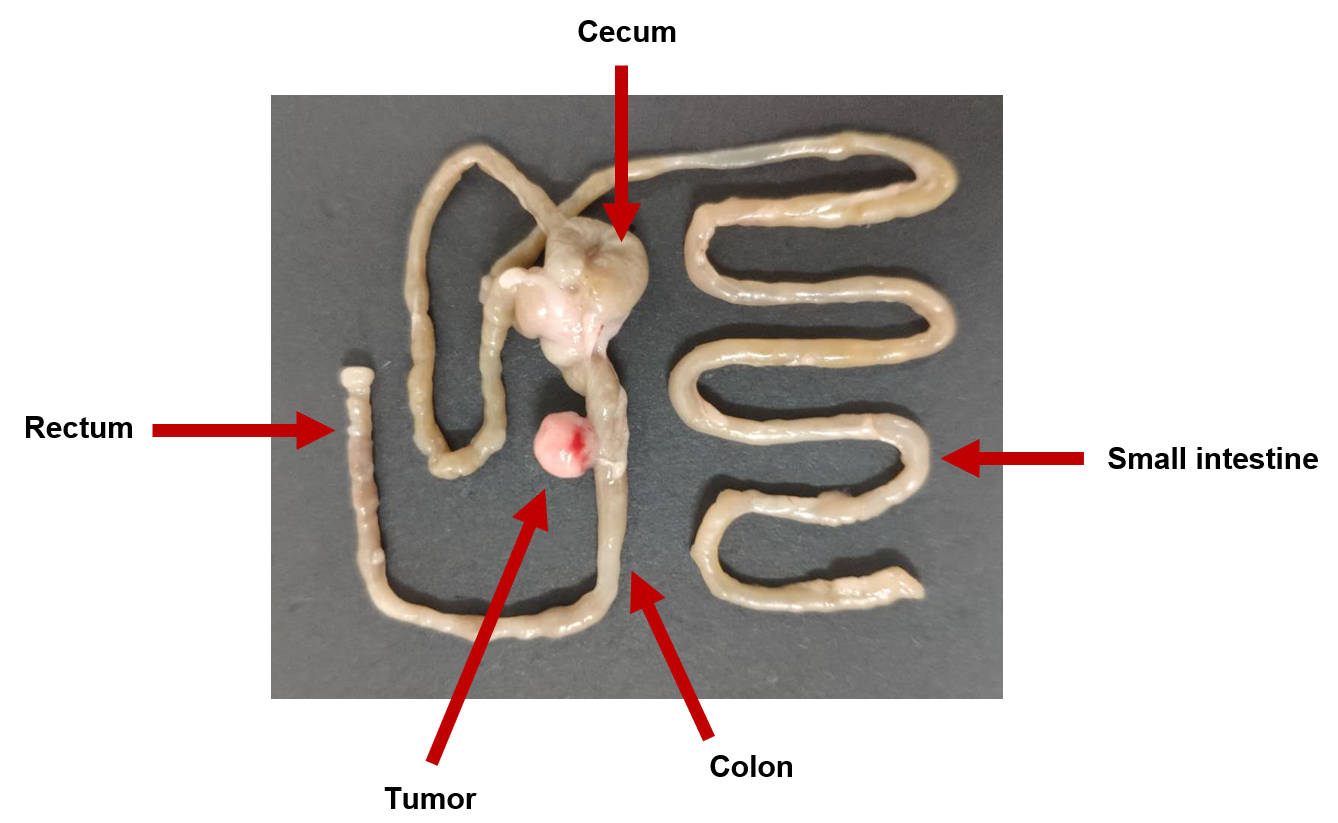


**Supplementary Figure 8.** Photograph of a representative CT-26 colonic orthotopic xenograft. The colon of BALB/c mice (6–7 weeks old) were inoculated with CT-26 cells (1 🞨 10^5^ cells/mouse) dispersed in Matrigel. The tumor successfully grew in the colon and not in other parts of the intestine.


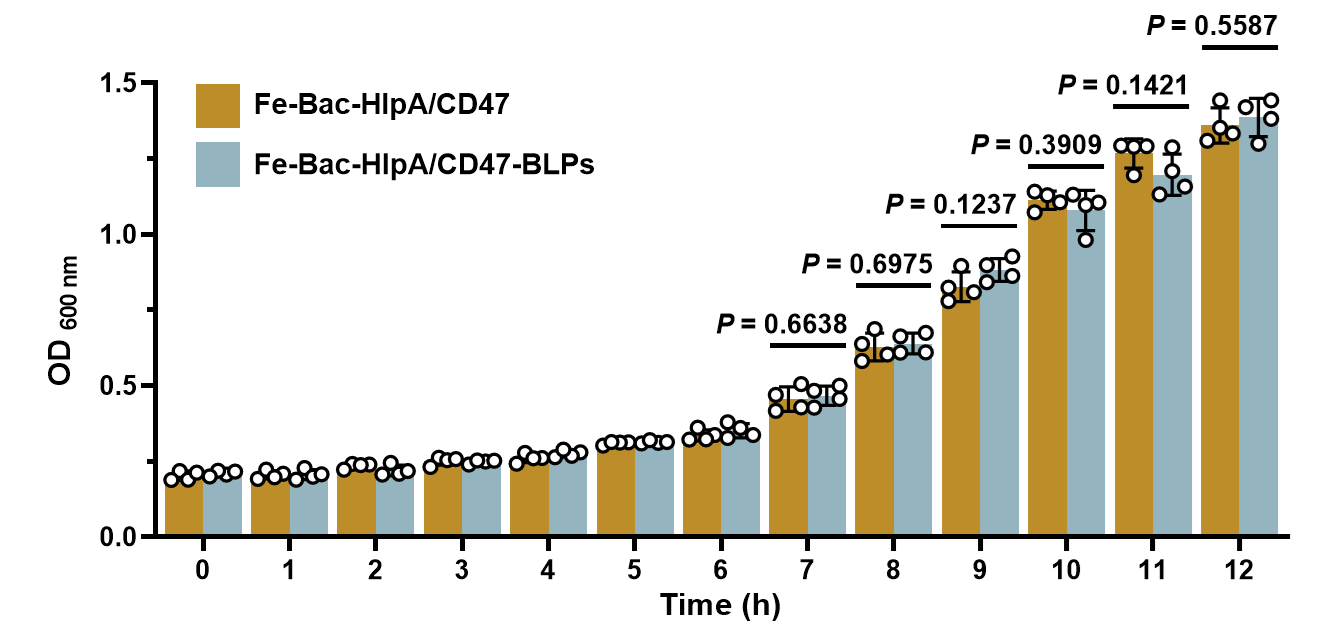


**Supplementary Figure 9**. The growth of Bac-HlpA/EGFP-BLPs and Bac-HlpA/EGFP at 30°C as monitored by OD_600_. The data are shown as the mean ± SD (n = 4 independent experiments). Statistical analysis was performed by a two-tailed unpaired *t* test. ^*^, *P* < 0.05; ^**^, *P* < 0.01; ^***^, *P* < 0.001. Source data are provided as a Source Data file.

**
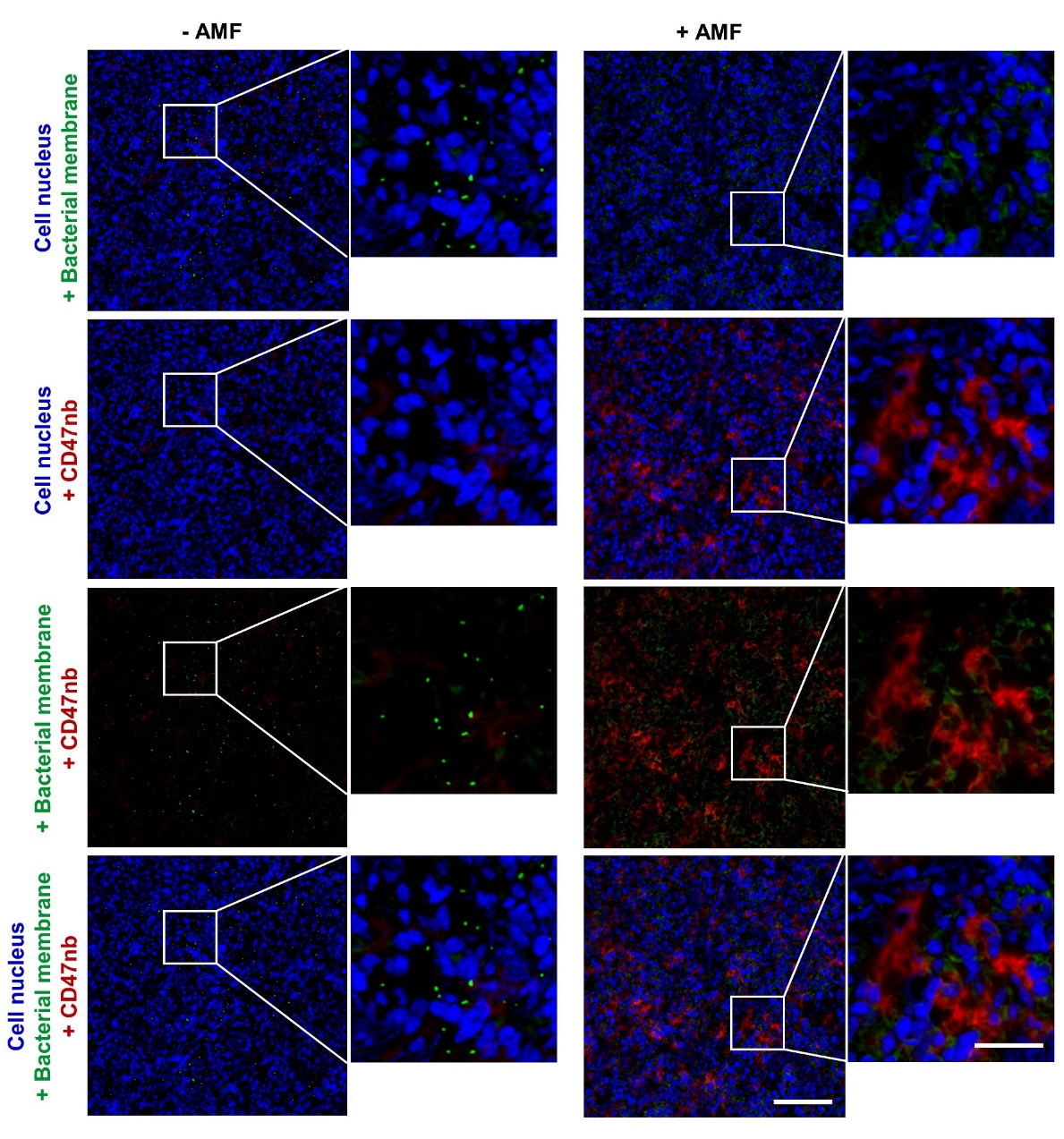
**

**Supplementary Figure 10**. The immunofluorescence images of tumor tissue sections exhibited the intratumoral release of CD47nb in the *in vivo* settings. Mice bearing CT-26 colonic orthotopic xenografts were administrated with Fe-Bac-HlpA/CD47-BLPs of 1 🞨 10^8^ CFUs, followed by AMF treatment (310 kHz and 23.8 kA/m) for 80 min at 24 h later. Tumors were collected at 4 h after AMF treatment, embedded into Opti-mum cutting temperature compound, and cut into 5 μm frozen sections. Bacterial membrane of Fe-Bac-HlpA/CD47-BLPs was stained with FITC-conjugated anti-*E. coli* O + K antibodies, which reacts with O and K antigenic serotypes of *E. coli* membrane. CD47nb was stained with Alexa Fluor^®^ 647-conjuaged anti-6X His tag^®^ antibody. Cell nucleus were stained with DAPI. Scale bar of left views, 100 μm; Scale bar of enlarged views, 30 μm. Each experiment was repeated six times independently.


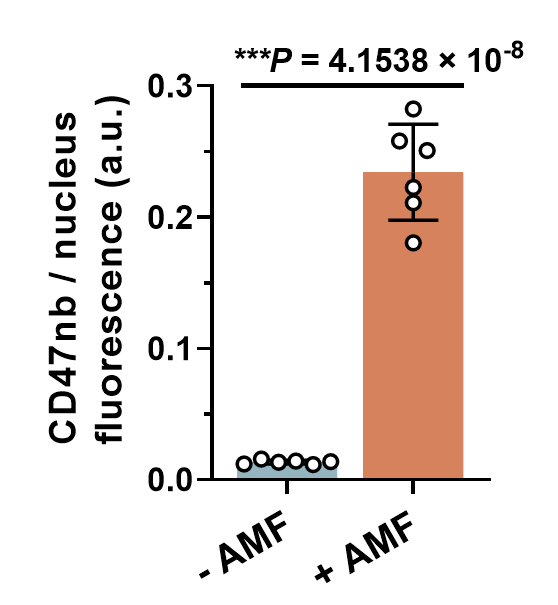


**Supplementary Figure 11**. Semi-quantification of the relative fluorescence intensity of CD47nb stained with Alexa Fluor^®^ 647-conjugated anti-6X His tag^®^ antibody in Supplementary Fig. 10, exhibiting the intratumoral release of CD47nb in the *in vivo* settings. The integrated optical intensity (IntDen) of Alexa Fluor^®^ 647 and DAPI was semi-quantified using the software ImageJ, and the IntDen ration of Alexa Fluor^®^ 647 to DAPI was calculated to compare the relative fluorescence intensity of CD47nb among two groups. The data are shown as the mean ± SD (n = 6 independent experiments). Statistical analysis was performed by a two-tailed unpaired *t* test. ^*^, *P* < 0.05; ^**^, *P* < 0.01; ^***^, *P* < 0.001. Source data are provided as a Source Data file.


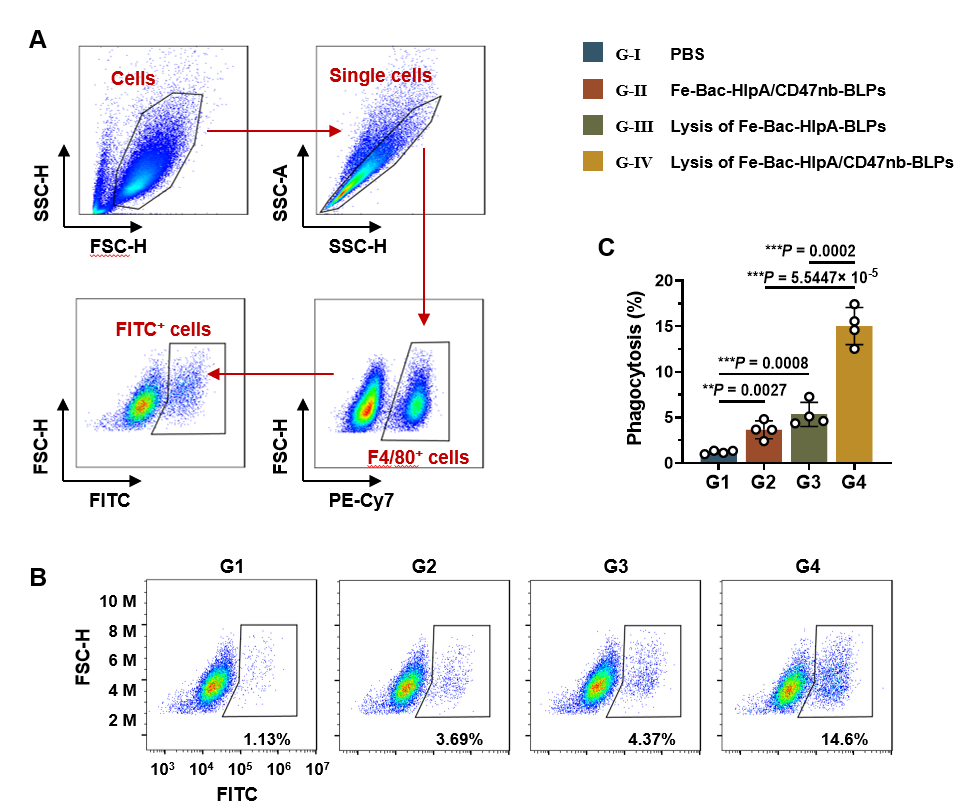


**Supplementary Figure 12**. Phagocytosis assay *in vitro* confirming that CD47nb of AMF-Bac blocked the interaction between CD47 on tumor cells and SIRPα on macrophages to induce phagocytosis. **(A)** Gating strategy of flow cytometry for phagocytosis assay *in vitro*. Green fluorescent protein (GFP)-transfected CT-26 cells (CT-26^GFP+^) were respectively incubated with PBS (G-I), Fe-Bac-HlpA/CD47nb-BLPs (G-II, 10^4^ CFU), lysis of Fe-Bac-HlpA-BLPs (G-III, 10^4^ CFU of Fe-Bac-HlpA-BLPs treated with AMF, 310 kHz and 23.8 kA/m, for 80 min), and lysis of Fe-Bac-HlpA/CD47nb-BLPs (G-IV, 10^4^ CFU of Fe-Bac-HlpA/CD47nb-BLPs treated with AMF) for 2 h at 4℃. Bone marrow-derived macrophages (BMDMs) were then co-cultured with the treated CT-26^GFP+^ cells at a ratio of 1:2 at 37°C for 2 h with the addition of 10,000 U/mL penicillin and streptomycin. The proportions of GFP^+^ cells in F4/80^+^ BMDMs were analyzed by flow cytometry to identify the BMDMs that engulfed CT-26^GFP+^ cells. **(B)** Representative dot plotting of flow cytometry showing the proportions of GFP^+^ cells in F4/80^+^ BMDMs. **(C)** Quantification results of panel B exhibiting the phagocytosis efficiency (the proportions of GFP^+^F4/80^+^ cells in F4/80^+^ BMDMs). The data (C) are shown as the mean ± SD (n = 4 independent experiments). Statistical analysis was performed by a two-tailed unpaired *t* test. ^*^, *P* < 0.05; ^**^, *P* < 0.01; ^***^, *P* < 0.001. Source data are provided as a Source Data file.


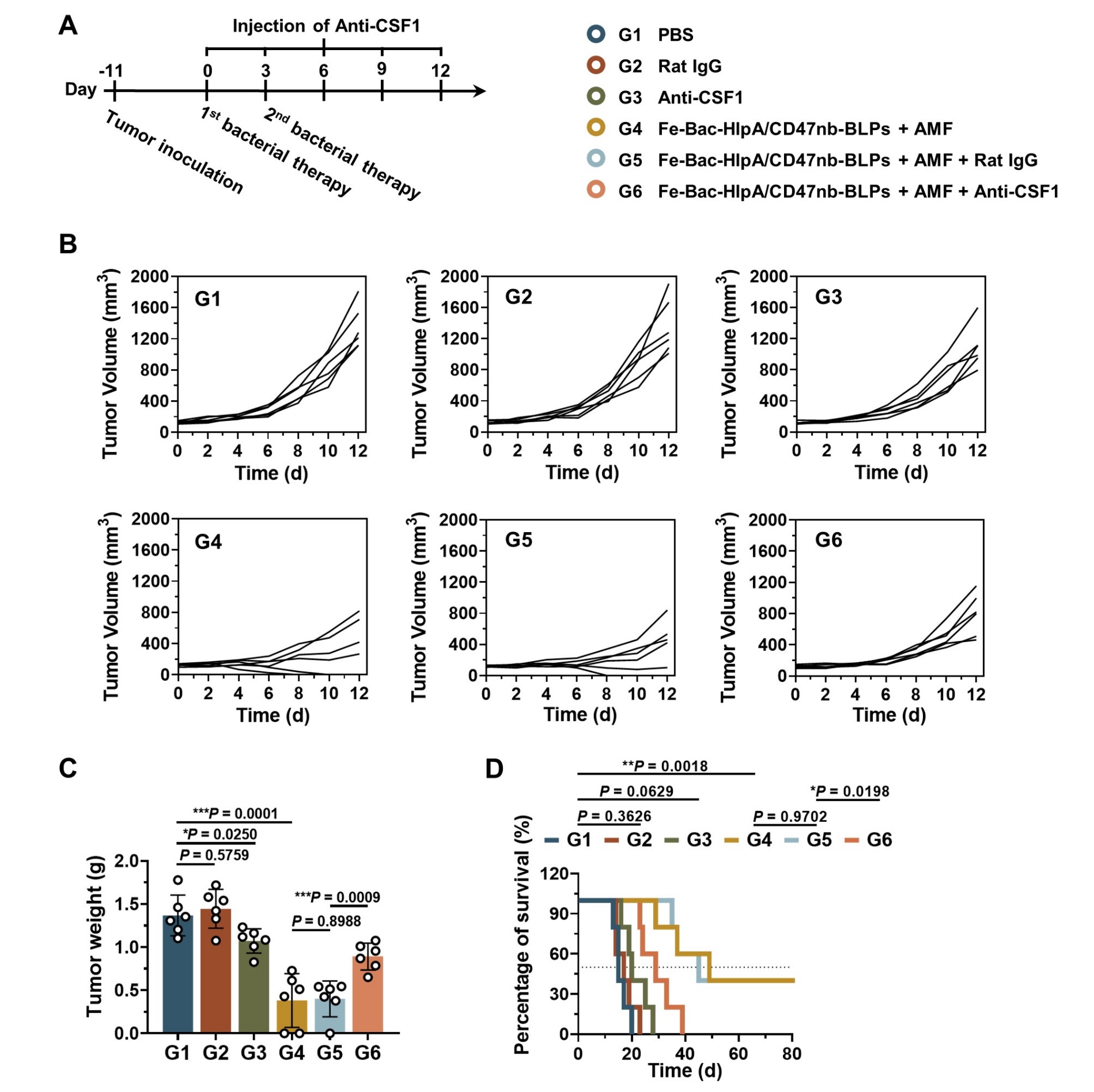


**Supplementary Figure 13**. *In vivo* antitumor therapy for investigating whether macrophage depletion could influence the therapeutic effects of AMF-Bac. **(A)** Scheme and grouping of *in vivo* therapy. BALB/c mice were subcutaneously inoculated with CT-26-luc cells (1 🞨 10^6^ cells/mouse) in the right hind limb on day -11 to allow the average tumor volume of ~120 mm^3^ at day 0. The mice were randomized into six groups with different treatments. For G2 and G5, mice were intratumorally injected with rat IgG1 isotype control (Rat IgG, 100 μg/mouse) at days 0, 3, 6, 9, and 12. For G3 and G6, mice were intratumorally injected with rat anti-mouse CSF1 neutralizing antibody (Anti-CSF1, 100 μg/mouse). For G4-G6, mice were intratumorally injected with Fe-Bac-HlpA/CD47nb-BLPs (1 🞨 10^3^ CFU) at days 0 and 3, followed by AMF treatment (310 kHz and 23.8 kA/m) for 80 min at 24 h after injection. **(B)** Change in tumor volume of the subcutaneous CT-26-luc (n = 6). **(C)** Tumor weight of the subcutaneous CT-26-luc xenografts measured on day 12 (n = 6 mice). **(D)** Survival curves of mice from the indicated groups for 80 days (n = 5 mice). The data (C) are shown as the mean ± SD. Statistical analysis was performed by a two-tailed unpaired *t* test. Survival significance was analyzed by the log-rank test. ^*^, *P* < 0.05; ^**^, *P* < 0.01; ^***^, *P* < 0.001. Source data are provided as a Source Data file.


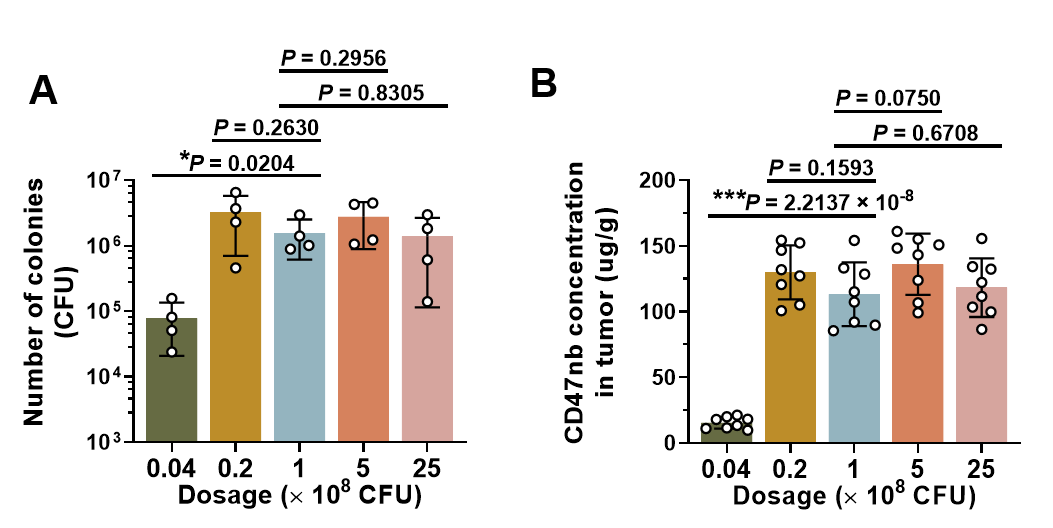


**Supplementary Figure 14**. **(A)** The intratumoral number of live bacteria after the administration of different dosage of Fe-Bac-HlpA/EGFP-BLPs. Mice bearing CT-26 colonic orthotopic xenografts were colon-specifically administrated with different dosage of Fe-Bac-HlpA/EGFP-BLPs (4 🞨 10^6^, 2 🞨10^7^, 1 🞨 10^8^, 5 🞨 10^8^, 2.5 🞨 10^9^ CFU/mouse). Tumors were collected and grounded at 24 h after administration. The suspension was serially diluted, and the number of live bacteria in the tumor was measured by the spread plate method (n = 4 tumors). **(B)** ELISA was performed to measure the intratumoral concentration of CD47nb. After the administration of different dosage of Fe-Bac-HlpA/EGFP-BLPs, AMF treatment (310 kHz and 23.8 kA/m) was given for 80 min at 24 h later. Tumors were collected after another 6 h, and the grinding suspension was added with concentrated extraction buffer (pH 7.4). The mixture was maintained with constant agitation for 2 h at 4°C, and centrifuged for 20 min at 10,000 g. The CD47nb in the supernatant was quantified with His-tag Protein ELISA kit (n = 8 tumors). The data are shown as the mean ± SD. Statistical analysis was performed by a two-tailed unpaired *t* test. ^*^, *P* < 0.05; ^**^, *P* < 0.01; ^***^, *P* < 0.001. Source data are provided as a Source Data file.


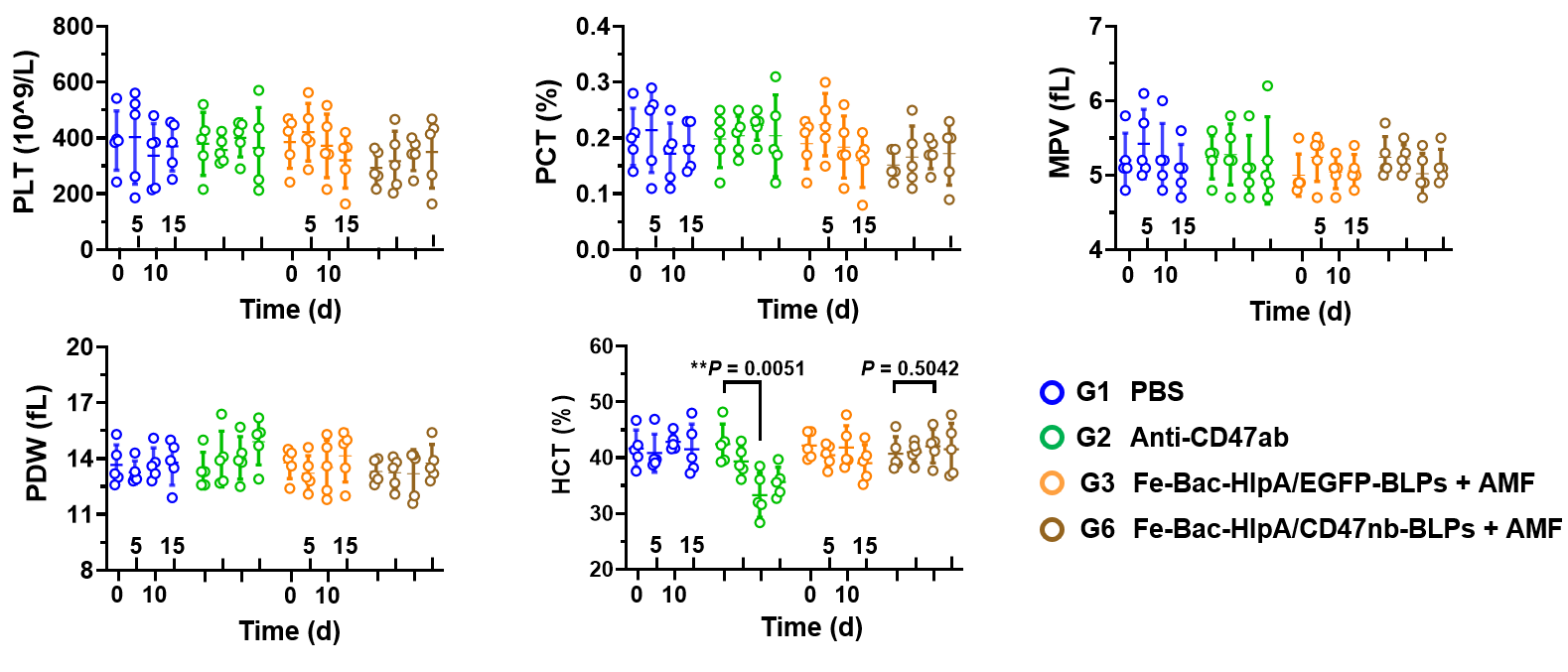


**Supplementary Figure 15.** Routine blood indices during therapy in the colonic orthotopic model (cf. Fig. 5A; n = 5 mice). PLT, platelet; PCT, plateletcrit; MPV, mean platelet volume; PDW platelet distributing width; HCT, hematocrit. The data are shown as the mean ± SD. Statistical analysis was performed by a two-tailed unpaired *t* test. ^*^, *P* < 0.05; ^**^, *P* < 0.01; ^***^, *P* < 0.001. Source data are provided as a Source Data file.


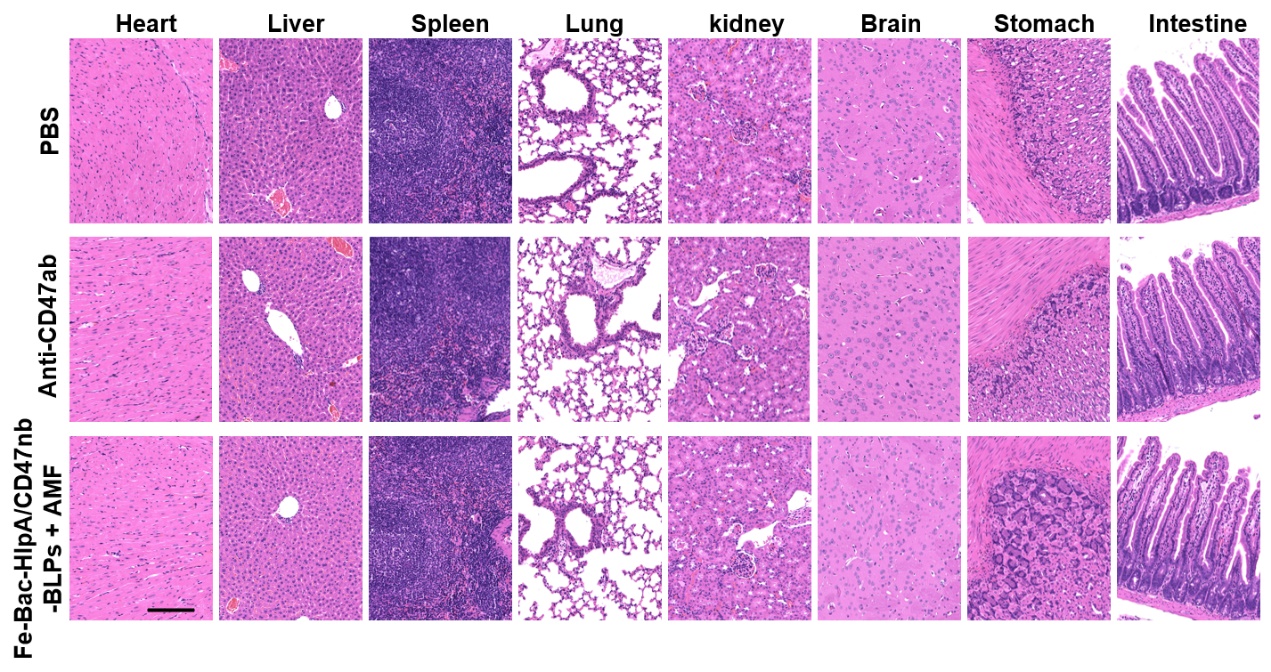


**Supplementary Figure 16.** Histopathological examination of H&E-stained sections of the major organs on day 15 in the colonic orthotopic model (cf. Fig. 5A). Scale bar, 150 μm. Each experiment was repeated three times independently with similar results. Source data are provided as a Source Data file.


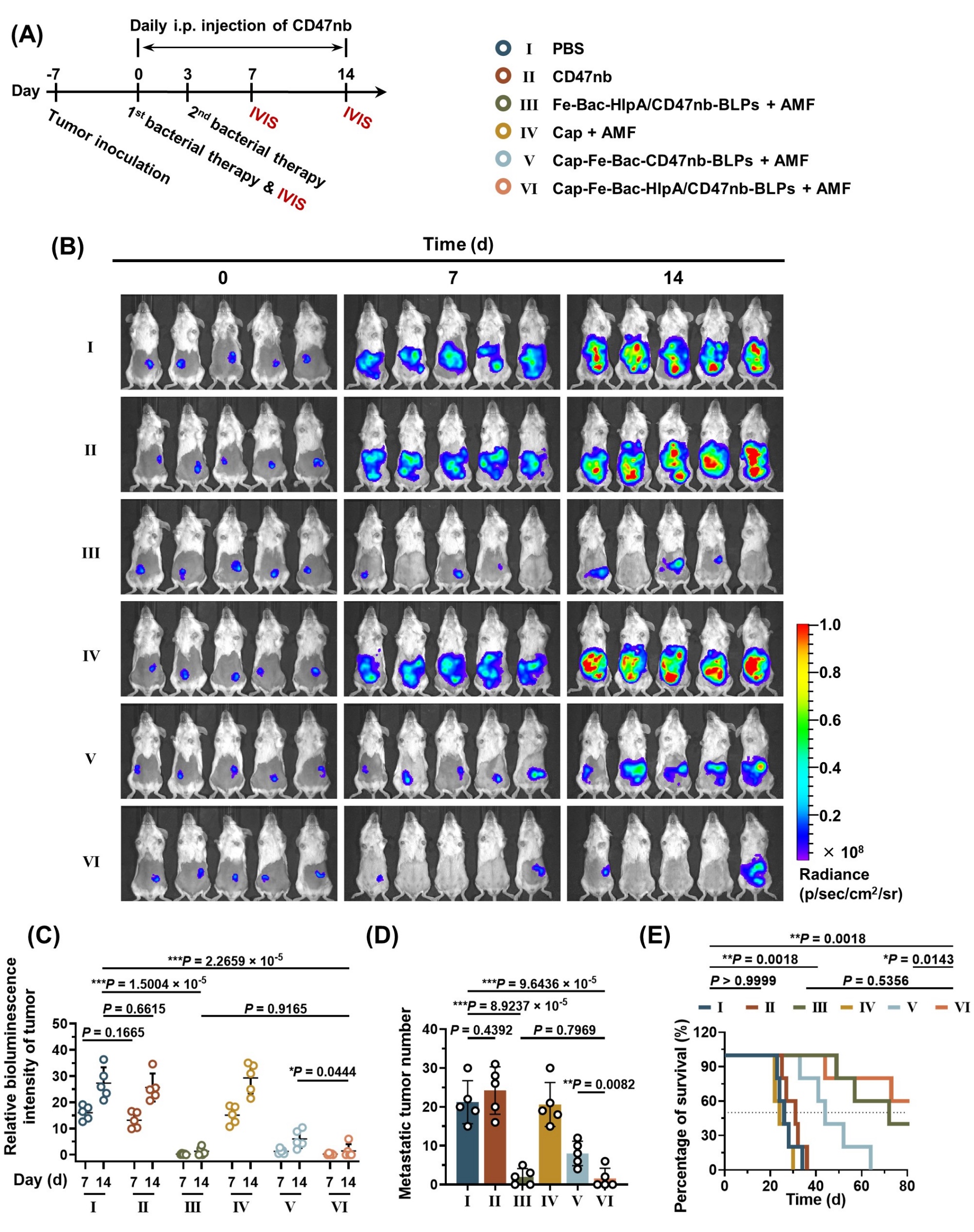


**Supplementary Figure 17.** Therapeutic effects of the engineered bacteria in the colonic orthotopic model. **(A)** Scheme and grouping for *in vivo* therapy (n = 5). The colons of BALB/c mice were inoculated with CT-26-luc cells (1 🞨 10^5^ cells/mouse) on day -7. For group III, the mice were treated with the indicated engineered bacteria (1 🞨 10^8^ CFU) by colon-specific administration on days 0 and 3. For group IV-VI, the mice were orally administrated with enteric capsule vesicles (Cap), or Cap-loaded engineered bacteria (Cap-Fe-Bac-HlpA/CD47nb-BLPs, 1 🞨 10^8^ CFU) on days 0 and 3. AMF treatment (310 kHz and 23.8 kA/m) was performed for 80 min at 24 h after colon-specific or oral administration. For group II, mice were administered with 20 mg/kg CD47nb daily by intraperitoneal injection for 14 consecutive days. **(B)** Bioluminescence imaging to monitor tumor growth performed on IVIS^®^ Spectrum *In vivo* Imaging System every 7 days (n = 5 mice). **(C)** Semi-quantitative results of the bioluminescence intensity of the tumor regions shown in panel B (n = 5 mice). The bioluminescence intensities of each mouse at day 7 and 14 were normalized to day 0. **(D)** The number of metastatic tumors in the abdomen were counted on day 15 (n = 5 mice). **(E)** Survival curves of mice from the indicated groups for 80 days (n = 5 mice). The data (C-E) are shown as the mean ± SD. Statistical analysis was performed by a two-tailed unpaired *t* test. Survival significance was analyzed by the log-rank test. ^*^, *P* < 0.05; ^**^, *P* < 0.01; ^***^, *P* < 0.001. Source data are provided as a Source Data file.

**
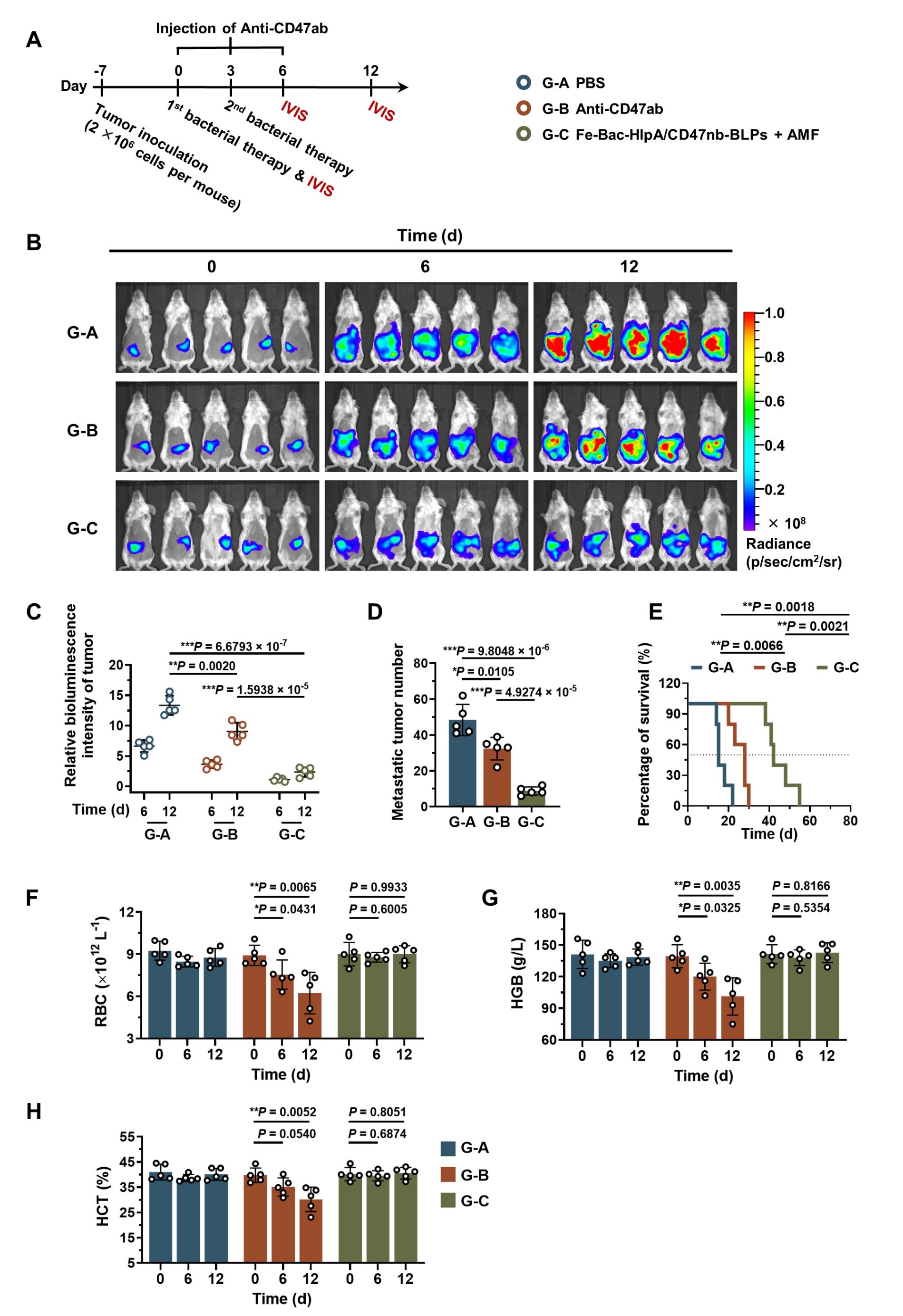
**

**Supplementary Figure 18**. The comparison of therapeutic effects between the engineered bacteria and anti-CD47 antibody performed on the colonic orthotopic model with large tumors. **(A)** Scheme and grouping of *in vivo* therapy (n = 5 mice). The colons of BALB/c mice were inoculated with CT-26-luc cells (2 🞩 10^6^ cells/mouse) on day -7. For group G-B, anti-CD47 antibody (Anti-CD47ab; 20 mg/kg) were *i.p.* injected on days 0, 3, and 6, respectively. For group G-C, mice were treated with the Fe-Bac-HlpA/CD47nb-BLPs (1 🞨 10^8^ CFU) by colon-specific administration on days 0 and 3, followed by AMF treatment (310 kHz and 23.8 kA/m) for 80 min at 24 h after colon administration. **(B)** Bioluminescence imaging to monitor tumor growth performed on IVIS^®^ Spectrum *In vivo* Imaging System every six days (n = 5 mice). **(C)** Semi-quantitative results of the bioluminescence intensity of the tumor regions shown in panel B (n = 5 mice). The bioluminescence intensities of each mouse at days 6 and 12 were normalized to day 0. **(D)** The number of metastatic tumors in the abdomen were counted on day 13 (n = 5 mice). **(E)** Survival curves of mice from the indicated groups for 80 days (n = 5). **(F-H)** The changes in red blood cell count (RBC; E), hemoglobin (HGB; F), and hematocrit (HCT; G) during therapy (n = 5 mice). The data (C-D, F-H) are shown as the mean ± SD. Statistical analysis was performed by a two-tailed unpaired *t* test. Survival significance was analyzed by the log-rank test. ^*^, *P* < 0.05; ^**^, *P* < 0.01; ^***^, *P* < 0.001. Source data are provided as a Source Data file.


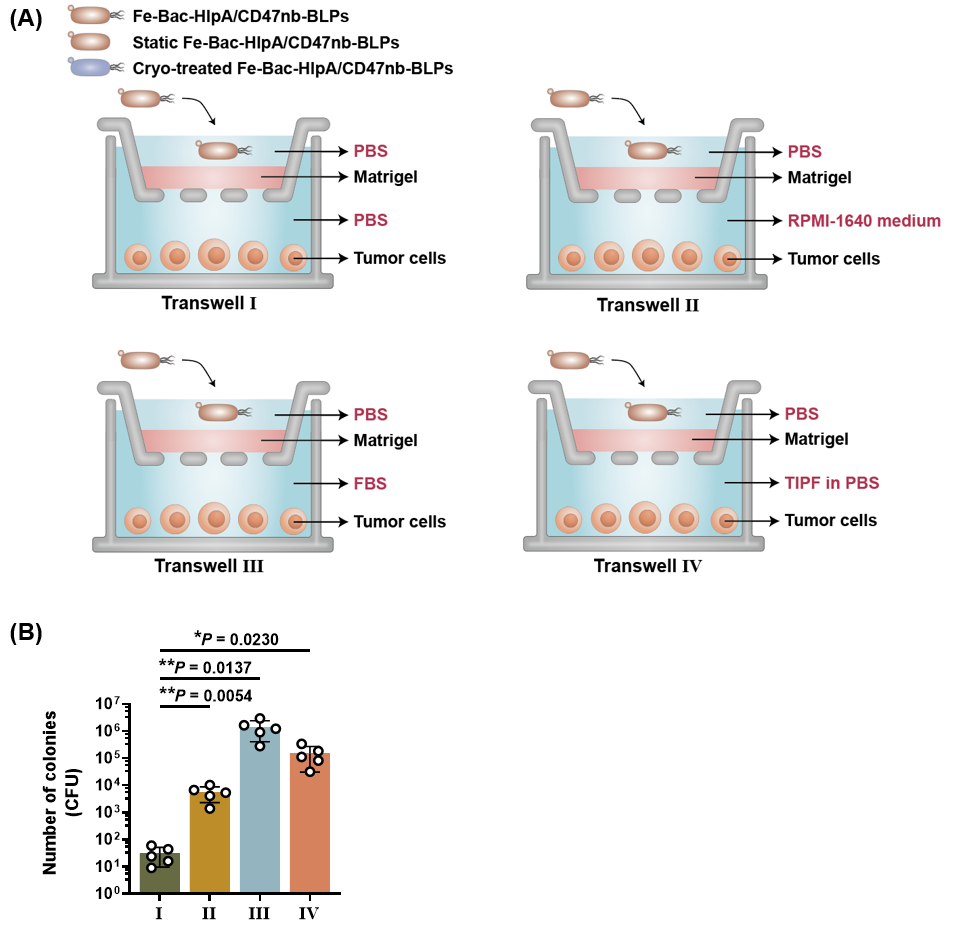


**Supplementary Figure 19.** Transwell assays for analyzing the propelling ability of AMF-Bac. **(A)** Illustration of the transwell assays. Different solutions were added into the basolateral chamber including PBS (Transwell I), RPMI-1640 medium (Transwell II), fetal calf serum (FBS, Transwell III), tumor interstitial perfusing fluid (TIPF) dissolved in PBS (Transwell IV). Motile, static, or cryo-treated Fe-Bac-HlpA/CD47nb-BLPs dispersed in PBS was added into the apical chamber. The assembled transwell devices was incubated at 37 ^o^C for 12 h, and the solution of basolateral chamber was sampled for measuring the number of bacteria. **(B)** The number of bacteria in the solution of basolateral chamber of panel A when motile Fe-Bac-HlpA/CD47nb-BLPs were added, which was measured by spread plate method (n = 5 chambers). No bacteria were detected in the basolateral chamber when static, or cryo-treated Fe-Bac-HlpA/CD47nb-BLPs were added. The bacterial number was measured by spread plate method. The data (B) are shown as the mean ± SD. Statistical analysis was performed by a two-tailed unpaired *t* test. ^*^, *P* < 0.05; ^**^, *P* < 0.01; ^***^, *P* < 0.001. Source data are provided as a Source Data file.


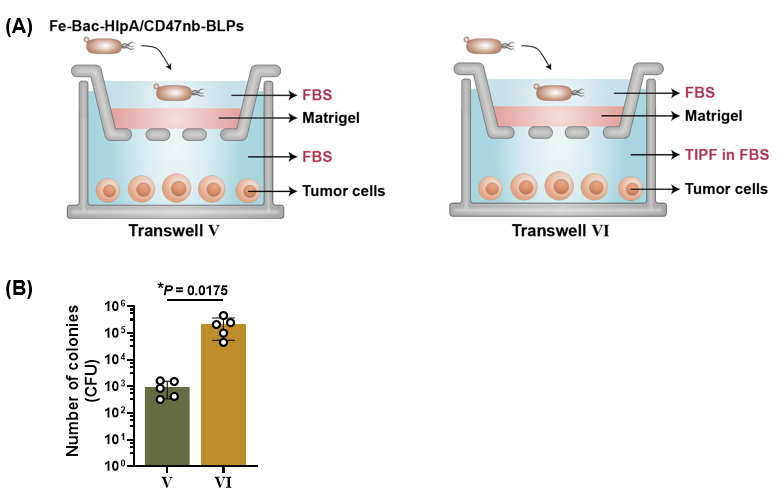


**Supplementary Figure 20. (A)** Illustration of transwell assays for analyzing the propelling ability of AMF-Bac. FBS was added to the apical chamber of these transwell devices, and different solutions was added into the basolateral chamber, including FBS (Transwell V) and TIPF dissolved in FBS (Transwell VI). **(B)** The number of bacteria in the solution of basolateral chamber of panel A measured by spread plate method (n = 5 chambers). The data (B) are shown as the mean ± SD. Statistical analysis was performed by a two-tailed unpaired *t* test. ^*^, *P* < 0.05; ^**^, *P* < 0.01; ^***^, *P* < 0.001. Source data are provided as a Source Data file.


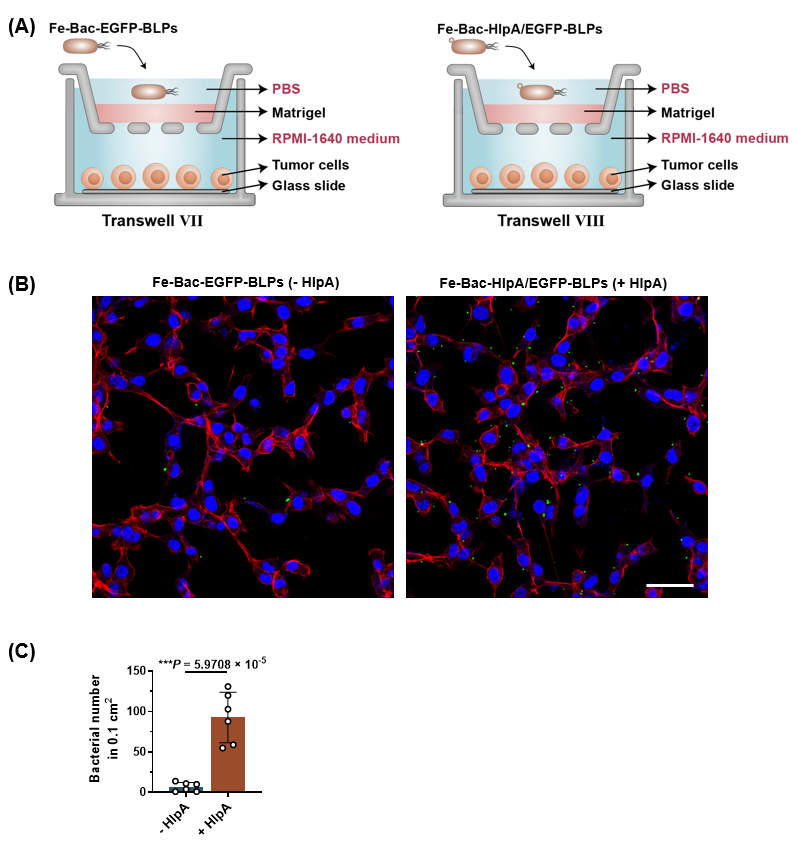


**Supplementary Figure 21.** Transwell assays for analyzing active navigation ability of AMF-Bac in long distance. **(A)** Illustration of the transwell assays. CT-26 cells dispersed in RPMI-1640 medium were added into the basolateral chamber, followed by the incubation at 37^o^C overnight. Fe-Bac-EGFP-BLPs (Transwell VII) or Fe-Bac-HlpA/CD47nb-BLPs (Transwell VIII) dispersed in PBS solution was added into the apical chamber. After the incubation for 12 h, grass slides on the basolateral chamber were collected for visualization of CLSM. **(B)** CLSM images of grass slides on the bottom of the basolateral chamber in panel A. The actin of tumor cells was stained with phalloidine, and cell nuclei were stained with DAPI. Bacteria were tracked by their expression of EGFP. Scale bar, 50 μm. Each experiment was repeated six times independently. **(C)** Quantification of the average number of bacteria in 0.1 cm^2^ field of CLSM view (n = 6 chambers). The data (C) are shown as the mean ± SD. Statistical analysis was performed by a two-tailed unpaired *t* test. ^*^, *P* < 0.05; ^**^, *P* < 0.01; ^***^, *P* < 0.001. Source data are provided as a Source Data file.


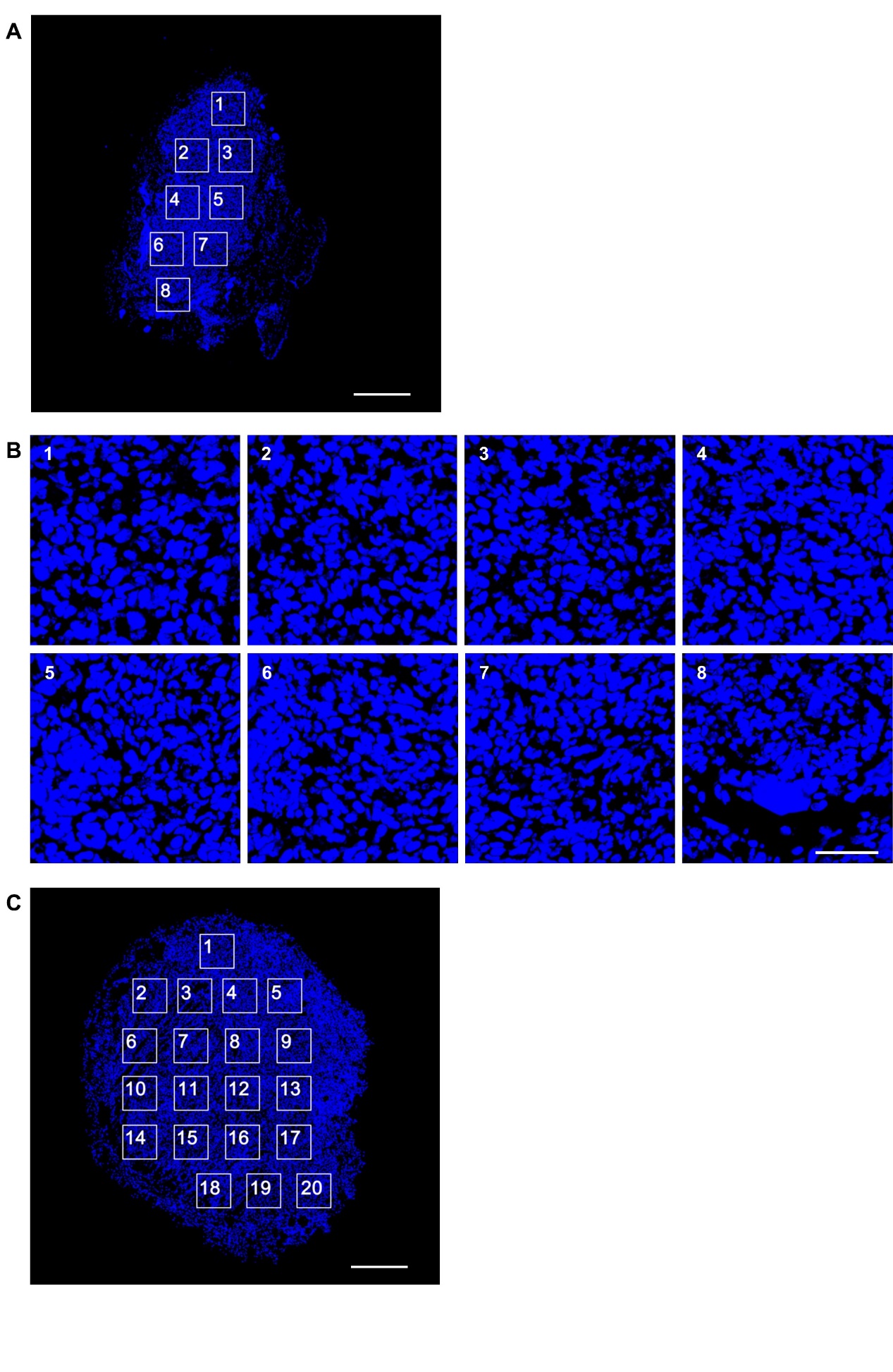


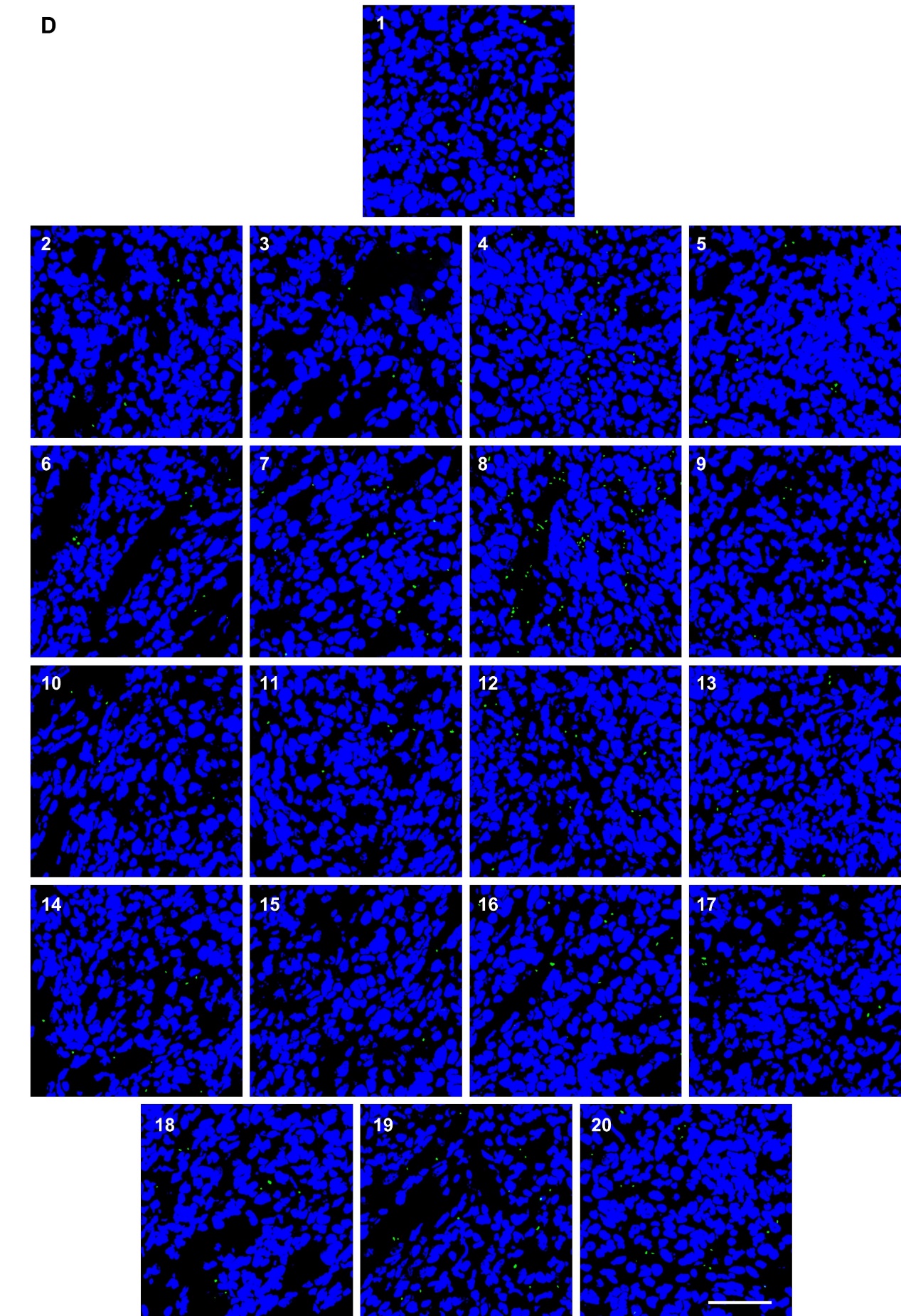


**Supplementary Figure 22.** CLSM images of tumor section for observation of the penetration ability and clearance rate of AMF-Bac. Mice bearing CT-26 colonic orthotopic xenografts were administrated with PBS (A-B) or Fe-Bac-HlpA/EGFP-BLPs of 1 🞨 10^8^ CFUs (C-D). 24 h after administration, tumors were collected, embedded into Opti-mum cutting temperature compound, and the frozen sections of tumors were stained with DAPI. Fe-Bac-HlpA/EGFP-BLPs in tumor sections was directly observed by CLSM using the fluorescence of bacterial EGFP. (A, C) Panorama images of tumor section. Scale bar of A and C, 300 μm; (B, D) Zoomed-in view of white boxes in A and C. Each experiment (A-D) was repeated three times independently with similar results. Scale bar of B and D, 50 μm.


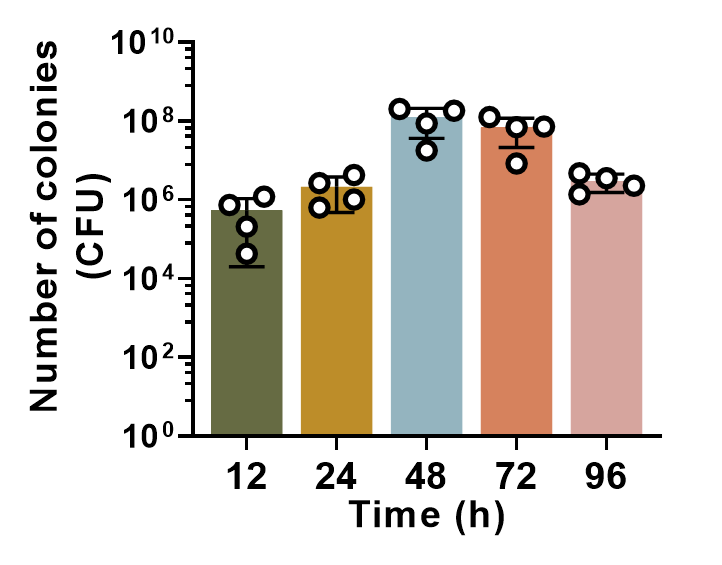


**Supplementary Figure 23.** The number of live bacteria in tumors measured at the different time after treating mice with AMF-Bac (n = 4 tumors). Mice bearing CT-26 colonic orthotopic xenografts were administrated with Fe-Bac-HlpA/EGFP-BLPs (1 🞨 10^8^ CFUs). At different time after administration (12-96 h), tumors were collected and grounded, and the suspension was serially diluted. The number of live bacteria in the tumor was measured by the spread plate method. The data are shown as the mean ± SD. Statistical analysis was performed by a two-tailed unpaired *t* test. ^*^, *P* < 0.05; ^**^, *P* < 0.01; ^***^, *P* < 0.001. Source data are provided as a Source Data file.


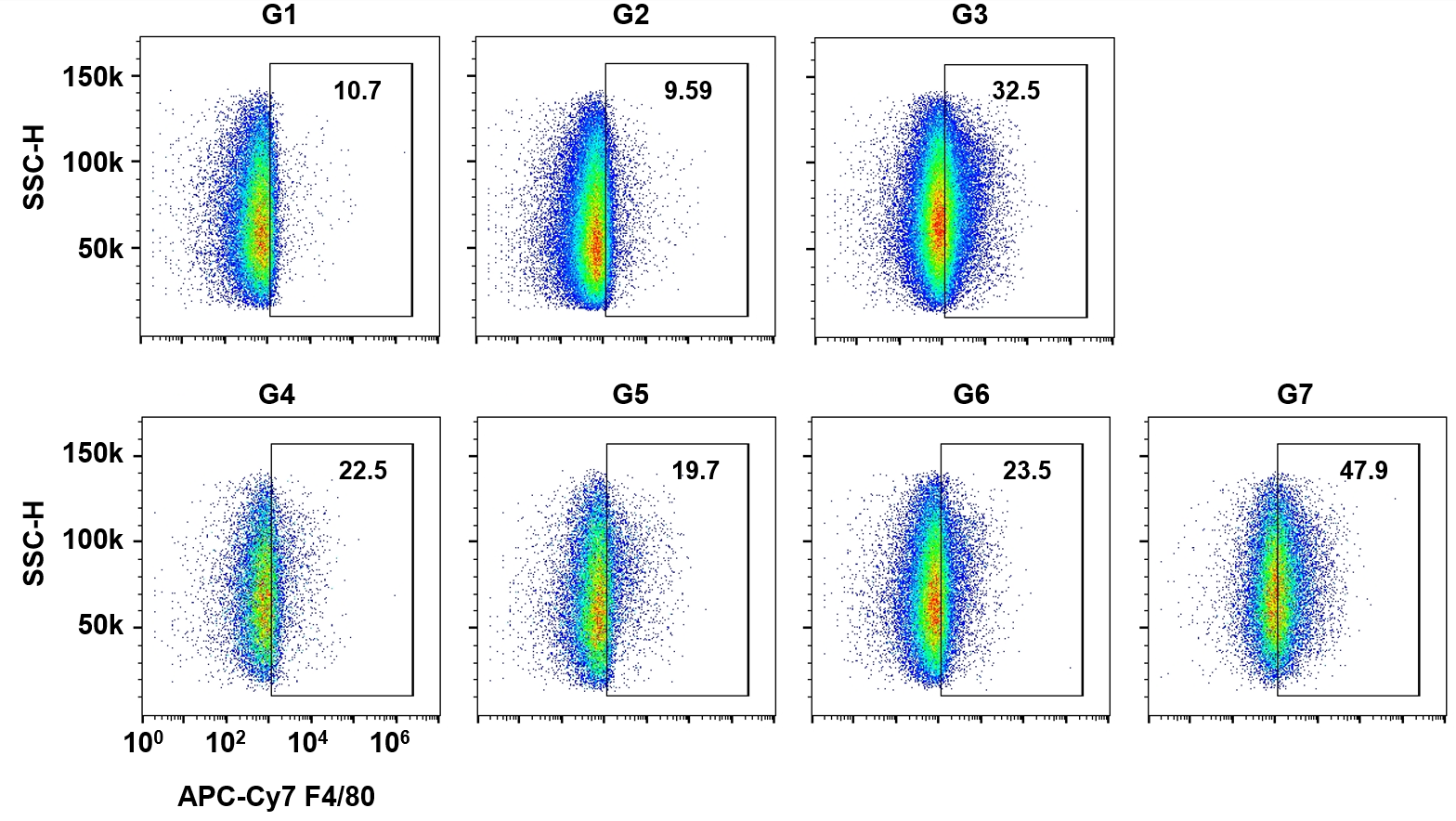


**Supplementary Figure 24.** Representative ﬂow cytometry dot plots showing the percentage of macrophages (F4/80^+^ cells) in tumors (cf. Fig. 6B).


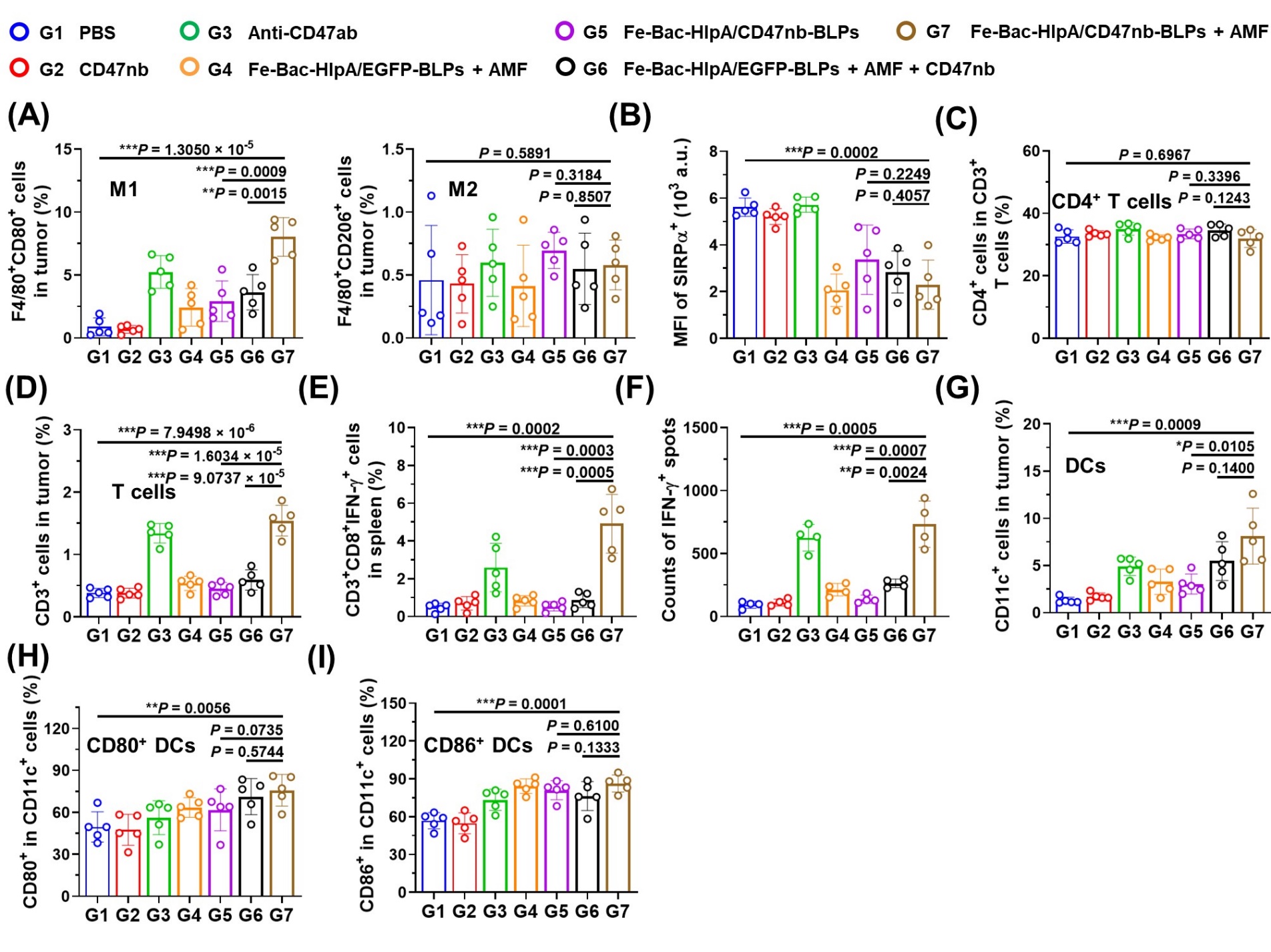


**Supplementary Figure 25.** **Immune responses induced by AMF-Bac.** **(A)** Flow cytometry analysis of the percentage of M1 (F4/80^+^CD80^+^ cells) and M2 macrophages (F4/80^+^CD206^+^ cells) in tumors (n = 5 mice). **(B)** Quantitative results of the levels of SIRPα in tumor-infiltrating macrophages, as analyzed using flow cytometry (cf. Fig. 6D, n = 5 mice). **(C-D)** Flow cytometry analysis of the percentage of CD4^+^ T cells (CD3^+^CD4^+^cells) in tumor-infiltrating CD3^+^ T cells (C) and the percentage of T cells (CD3^+^ cells) in tumors (D; n = 5 mice). **(E)** Flow cytometry analysis of the percentage of CD3^+^CD8^+^IFN-γ^+^ cells in splenocytes after re-stimulation with the CT26-specific antigen peptide (n = 5 mice). **(F)** Quantitative results of the data presented in Fig. 6J (n = 4 mice). **(G-I)** Flow cytometry analysis of the percentage of DCs (CD11c^+^ cells) in tumors (G) and the percentages of matured DCs (CD11c^+^CD80^+^ and CD11c^+^CD86^+^ cells) in tumor-infiltrating DCs (H-I; n = 5 mice). The data are shown as the mean ± SD. Statistical analysis was performed by a two-tailed unpaired *t* test. ^*^, *P* < 0.05; ^**^, *P* < 0.01; ^***^, *P* < 0.001. Source data are provided as a Source Data file.


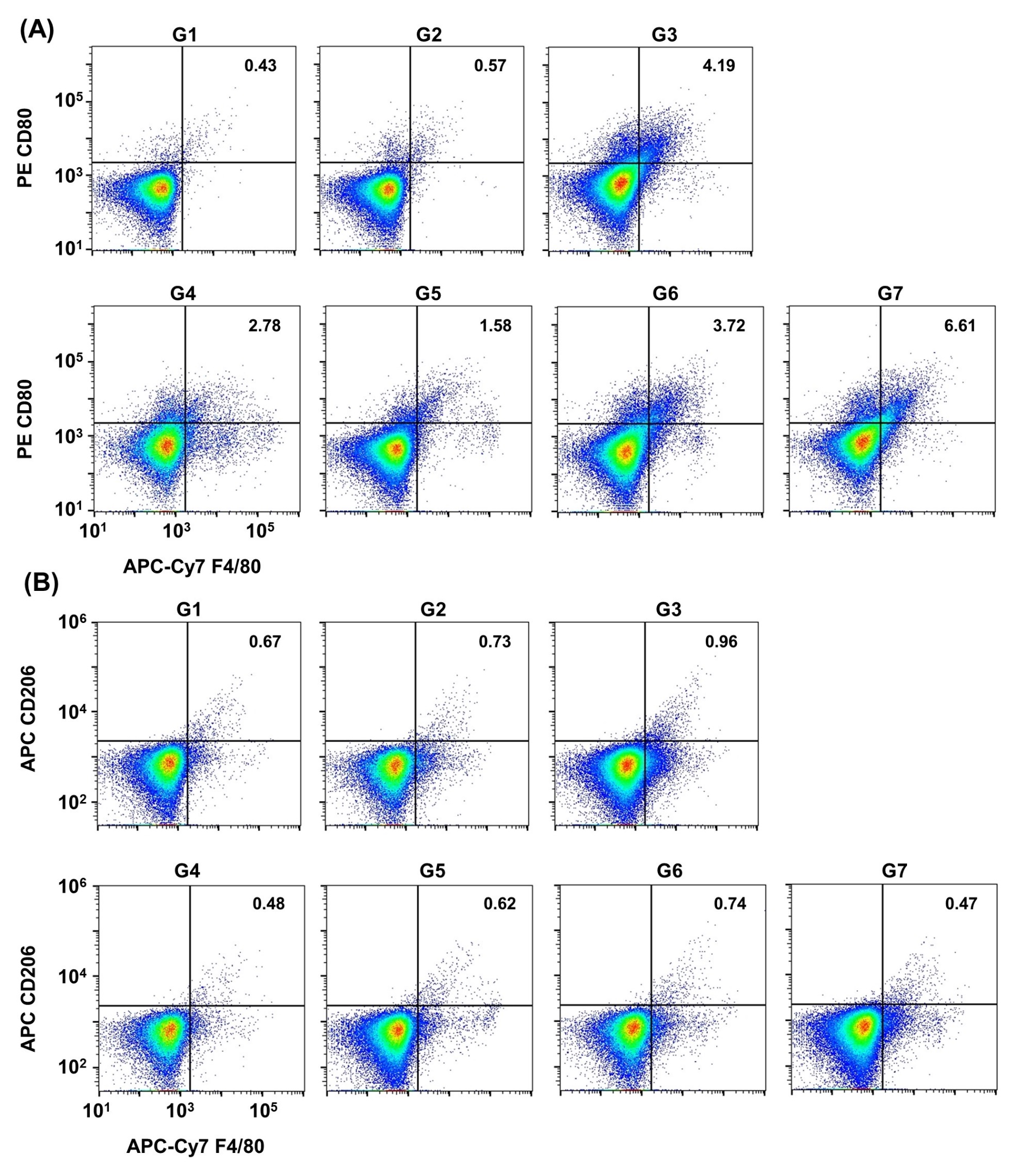


**Supplementary Figure 26.** Representative ﬂow cytometry dot plots showing the percentages of M1 (F4/80^+^CD80^+^ cells; A) and M2 macrophages (F4/80^+^CD206^+^ cells; B) in tumors (cf. Supplementary Figure 25A).


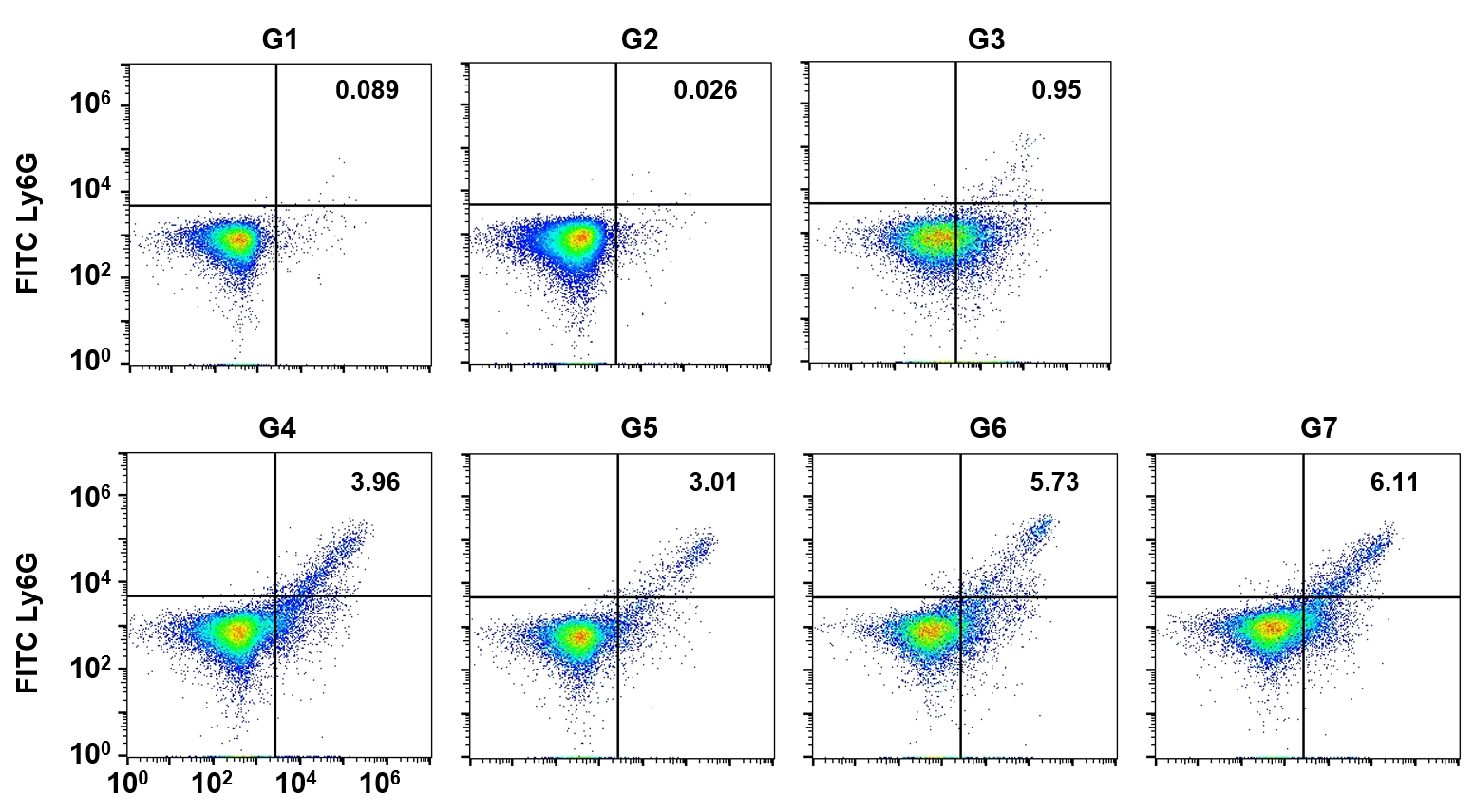


**Supplementary Figure 27.** Representative ﬂow cytometry dot plots showing the percentage of tumor-infiltrating neutrophils (CD11b^+^Ly6G^+^ cells) in tumors (cf. Fig. 6E).


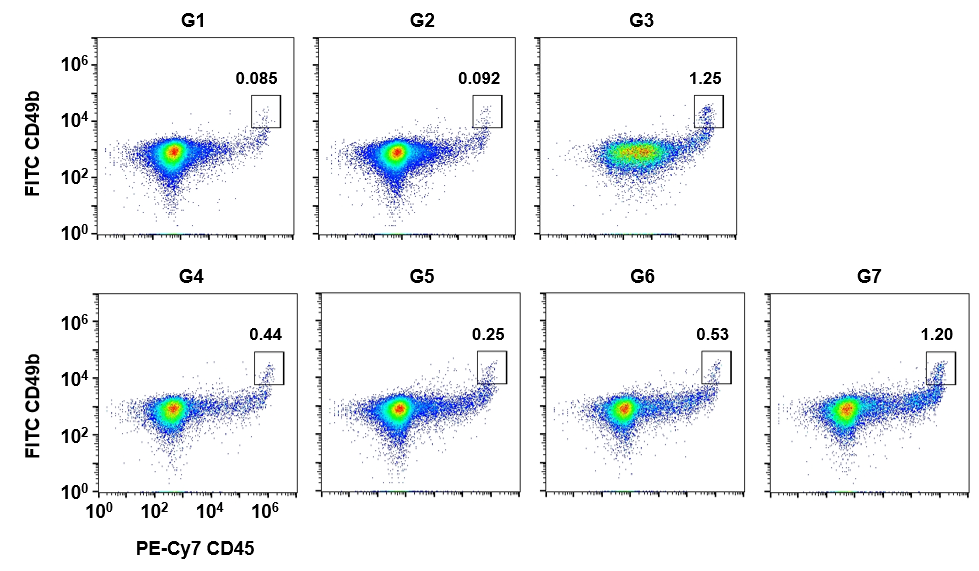


**Supplementary Figure 28.** Representative ﬂow cytometry dot plots showing the percentage of tumor-infiltrating NK cells (CD45^+^CD49b^+^ cells) in tumors (cf. Fig. 6F).


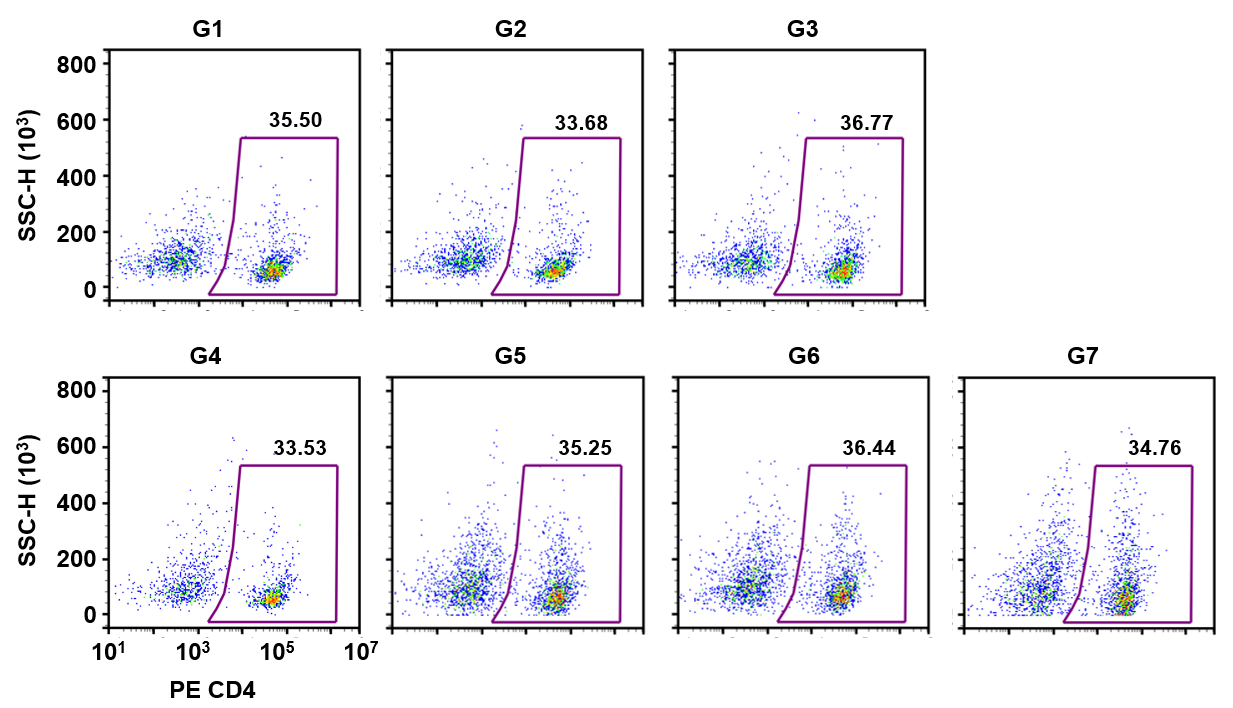


**Supplementary Figure 29.** Representative ﬂow cytometry dot plots showing the percentage of CD4^+^ T cells (CD3^+^CD4^+^cells) in tumor-infiltrating CD3^+^ T cells (cf. Supplementary Figure 25C).


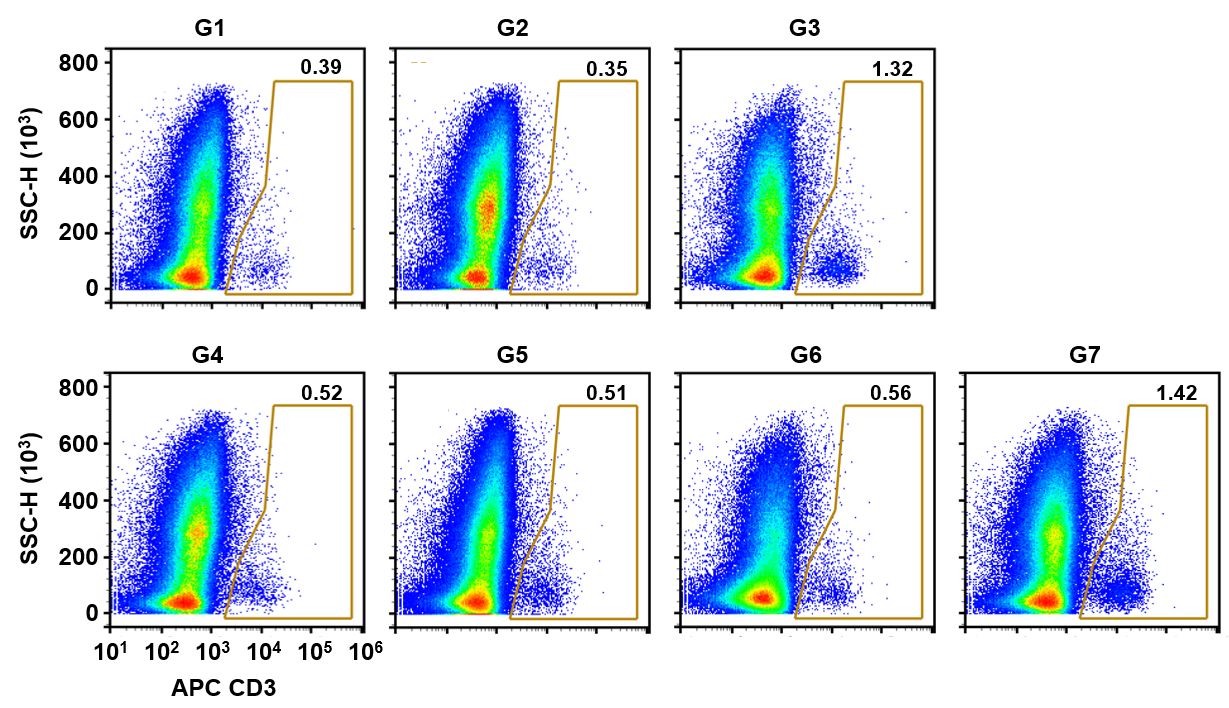


**Supplementary Figure 30.** Representative ﬂow cytometry dot plots showing the percentage of CD3^+^ T cells in tumors (cf. Supplementary Figure 25D).


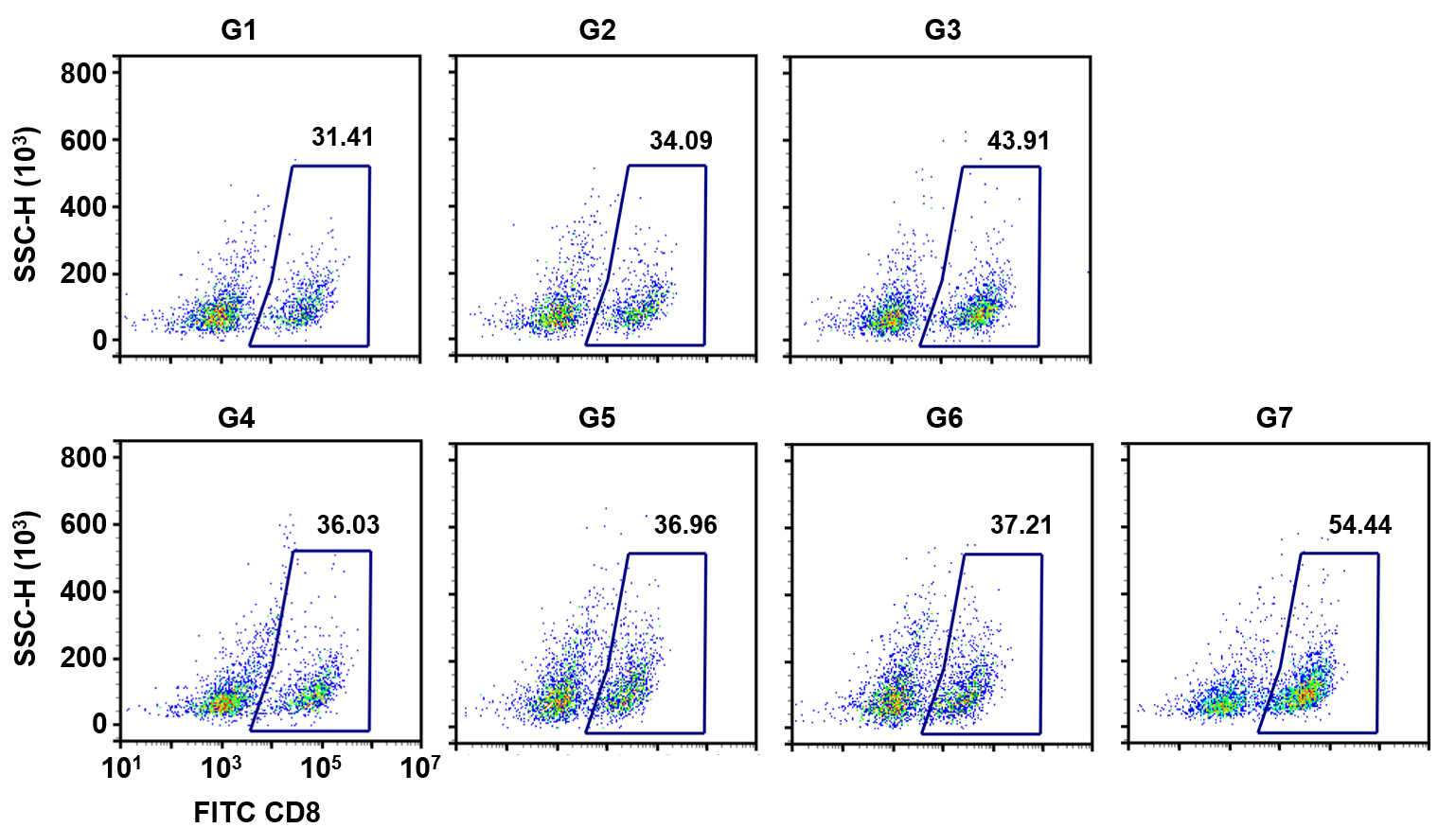


**Supplementary Figure 31.** Representative ﬂow cytometry dot plots showing the percentage of CD8^+^ T cells (CD3^+^CD8^+^cells) in tumor-infiltrating CD3^+^ T cells (cf. Fig. 6G).


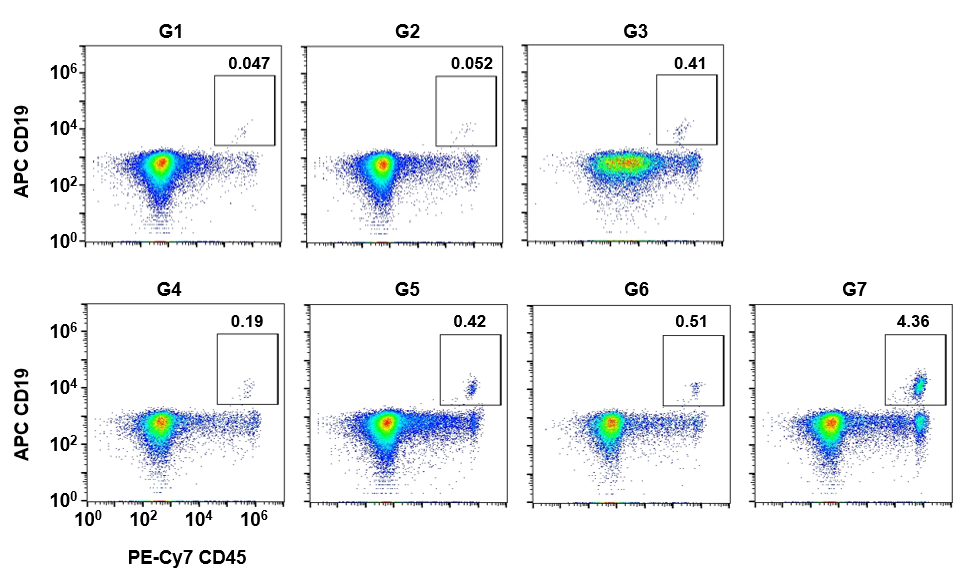


**Supplementary Figure 32.** Representative ﬂow cytometry dot plots showing the percentage of tumor-infiltrating B cells (CD45^+^CD19^+^ cells) in tumors (cf. Fig. 6I).


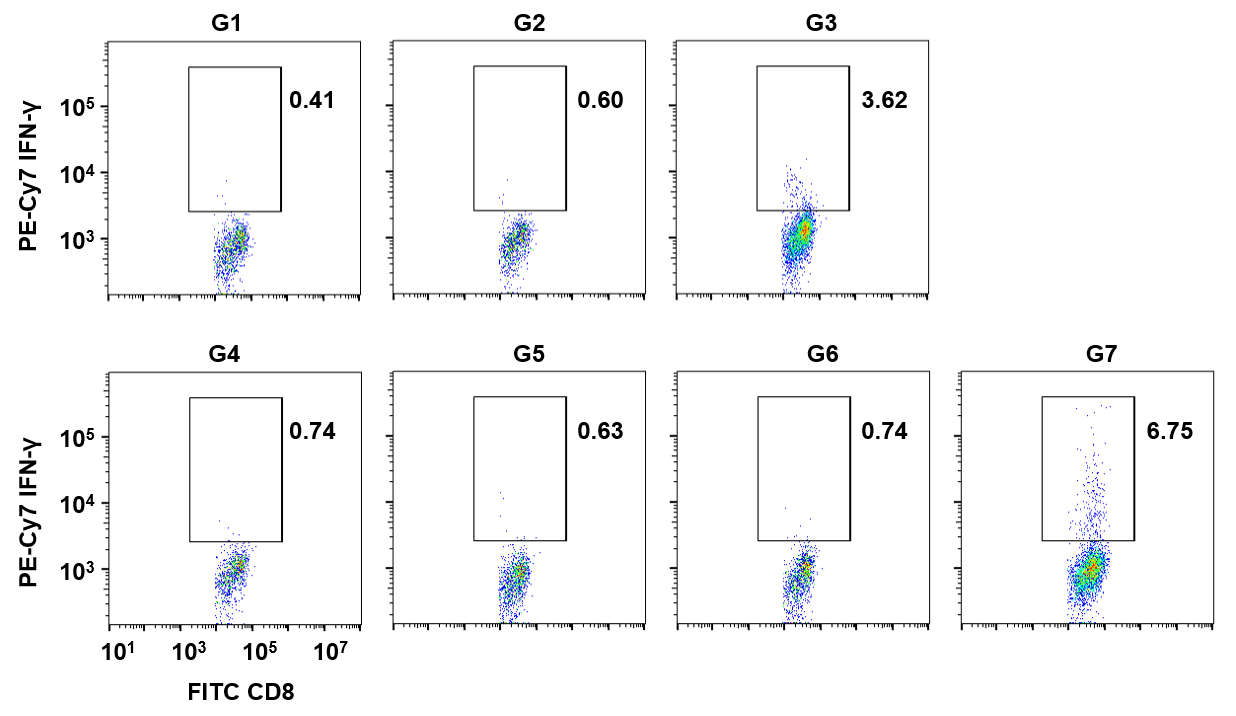


**Supplementary Figure 33.** Representative ﬂow cytometry dot plots showing the percentage of CD3^+^CD8^+^IFN-γ^+^ cells in splenocytes after re-stimulation with the CT26-specific antigen peptide (cf. Supplementary Figure 25E).


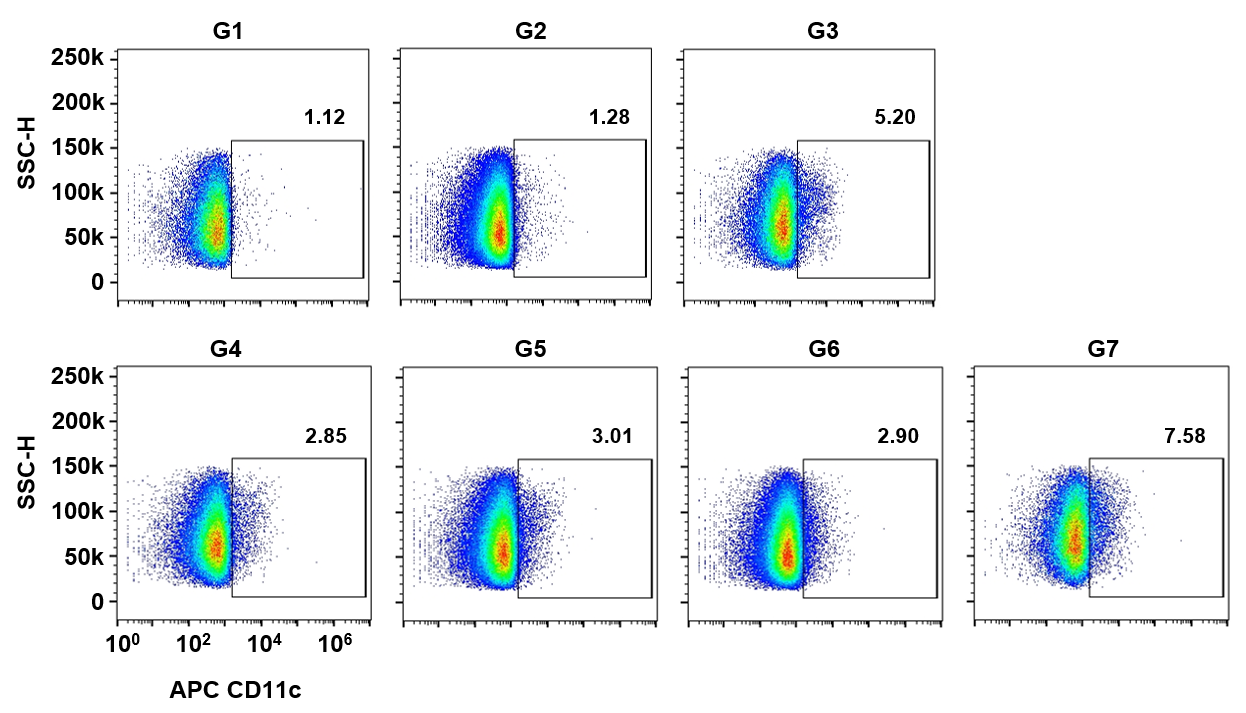


**Supplementary Figure 34.** Representative ﬂow cytometry dot plots showing the percentage of DCs (CD11c^+^ cells) in tumors (cf. Supplementary Figure 25G).


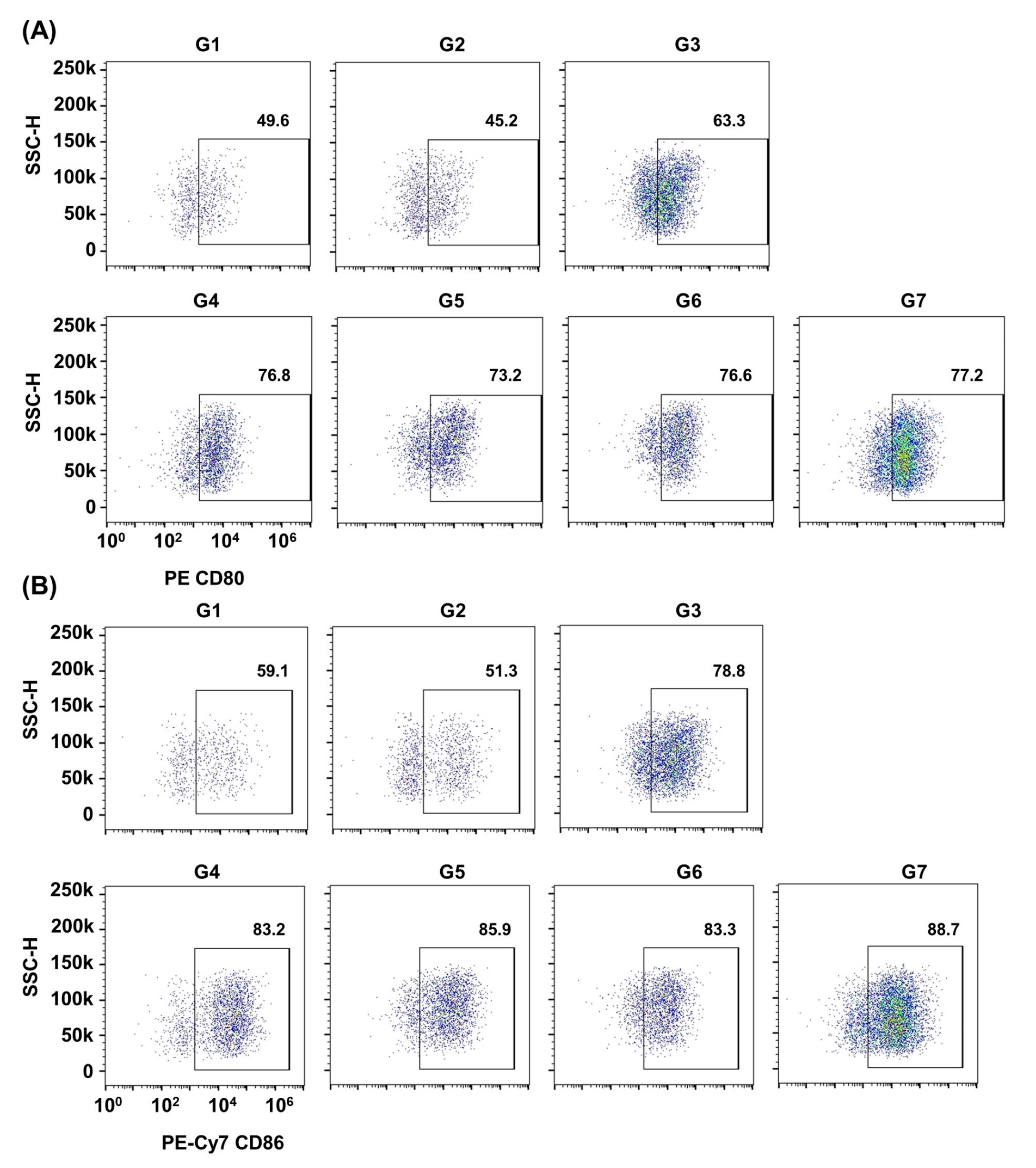


**Supplementary Figure 35.** (A-B) Representative ﬂow cytometry dot plots showing the percentages of the mature DCs (CD11c^+^CD80^+^ and CD11c^+^CD86^+^ cells) in tumor-infiltrating DCs (cf. Supplementary Figure 25H and S25I).


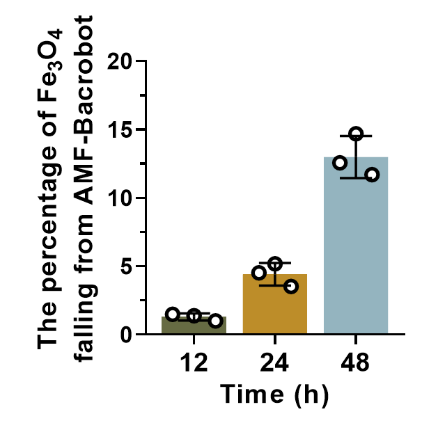


**Supplementary Figure 36**. The percentage of free Fe_3_O_4_ nanoparticles falling from the AMF-Bac after 12-48 h of proliferation. Fe-Bac-HlpA/EGFP-BLPs was cultured in LB medium at 37^o^C for 12, 24 and 48 h, followed by the centrifugation at 500 g for 10 min. The concentration of free Fe_3_O_4_ nanoparticles in supernatant was measured by ICP-AES (inductive coupled plasma-atomic emission spectrometer). The data are shown as the mean ± SD (n = 3 independent experiments). Source data are provided as a Source Data file.


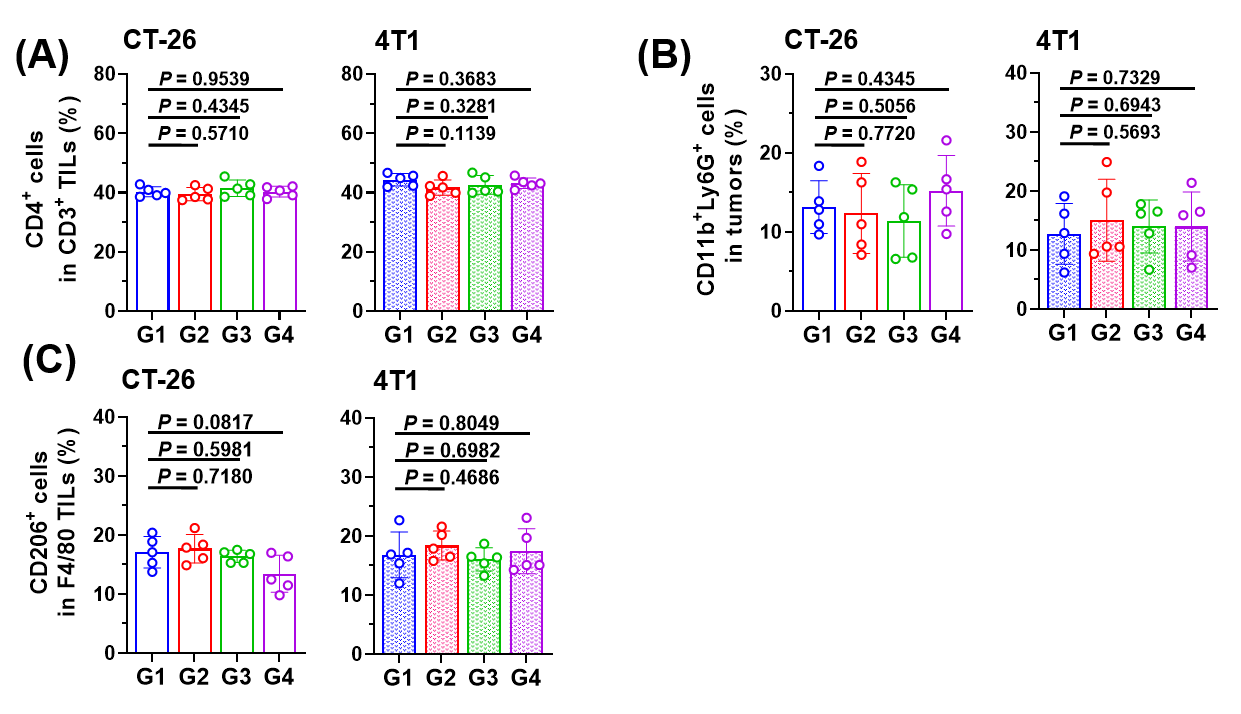


**Supplementary Figure 37.** Immune cell analysis to evaluate the abscopal effect (cf. Fig. 8A). **(A-C)** Flow cytometry analysis of the percentage of tumor-infiltrating CD4^+^ T cells in the total tumor-infiltrating CD3^+^ cells (A), the percentage of neutrophils (CD11b^+^Ly6G^+^ cells) in tumors (B) and the percentage of M2 macrophages (F4/80^+^CD206^+^ cells) in tumor-infiltrating macrophages (C). The data are shown as the mean ± SD (n = 5 mice). Statistical analysis was performed by a two-tailed unpaired *t* test. ^*^, *P* < 0.05; ^**^, *P* < 0.01; ^***^, *P* < 0.001. Source data are provided as a Source Data file.


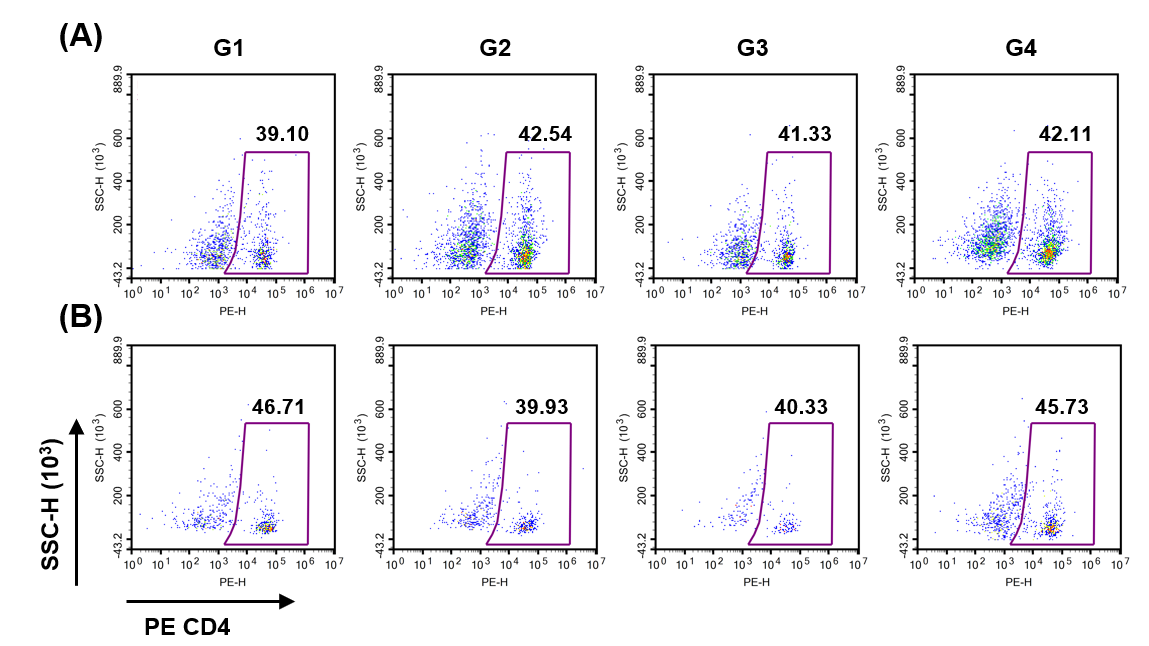


**Supplementary Figure 38.** Representative ﬂow cytometry dot plots showing the percentage of tumor-infiltrating CD4^+^ T cells (CD3^+^CD4^+^cells) in the total tumor-infiltrating CD3^+^ T cells (cf. Supplementary Figure 37A). **(A)** CT26 xenograft. **(B)** 4T1 xenograft.


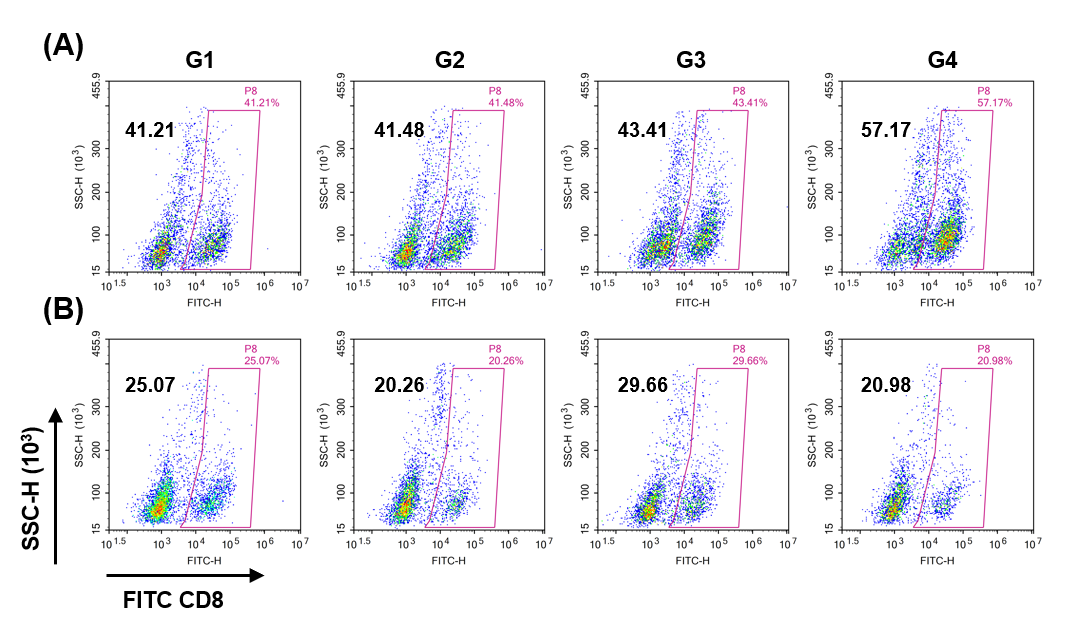


**Supplementary Figure 39.** Representative ﬂow cytometry dot plots showing the percentage of tumor-infiltrating CD8^+^ T cells (CD3^+^CD8^+^cells) in the total tumor-infiltrating CD3^+^ T cells (cf. Fig. 8D). **(A)** CT26 xenograft. **(B)** 4T1 xenograft.


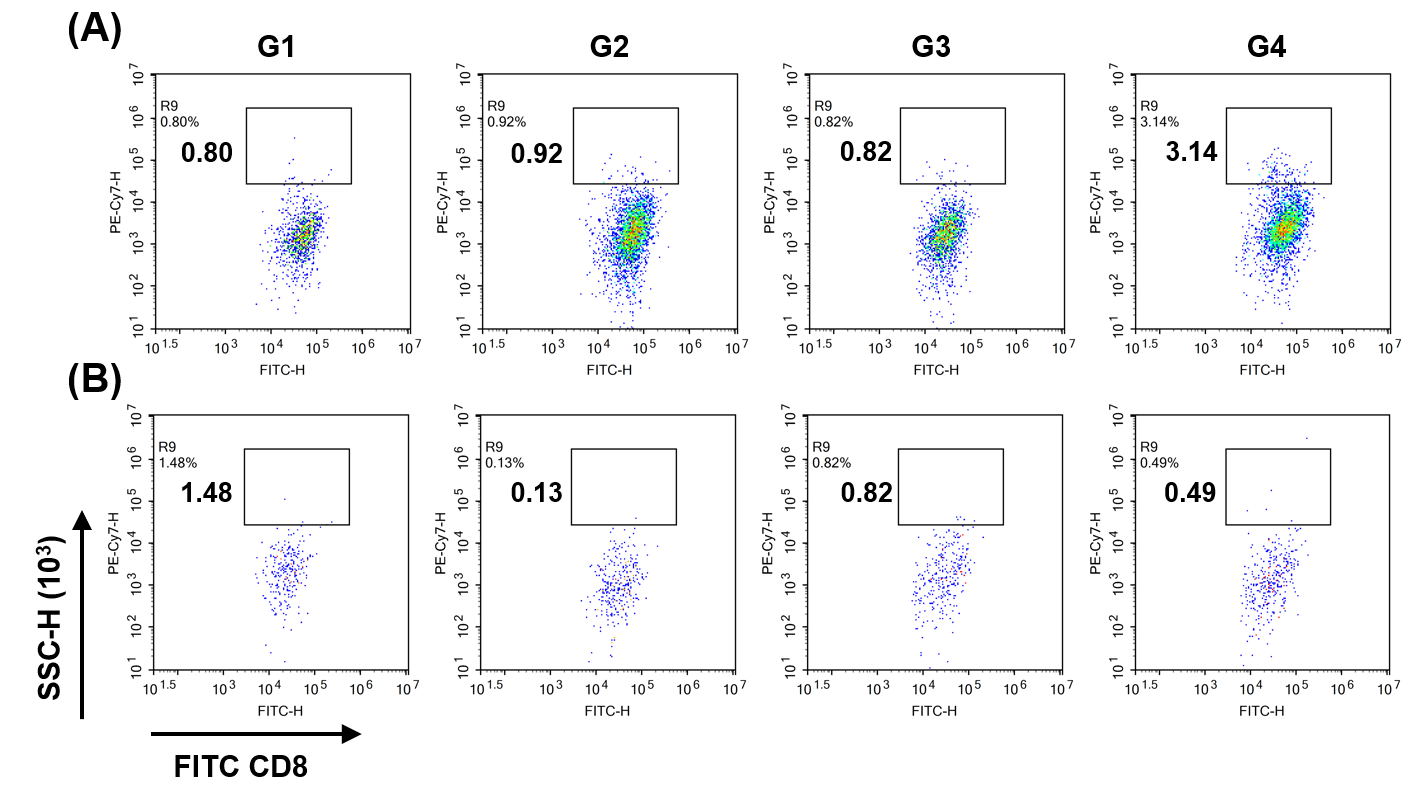


**Supplementary Figure 40.** Representative ﬂow cytometry dot plots showing the percentage of tumor-infiltrating effector CD8^+^ T cells (CD3^+^CD8^+^IFN-γ^+^ cells) in the total tumor-infiltrating CD3^+^CD8^+^ T cells (cf. Fig. 8E). **(A)** CT26 xenograft. **(B)** 4T1 xenograft.


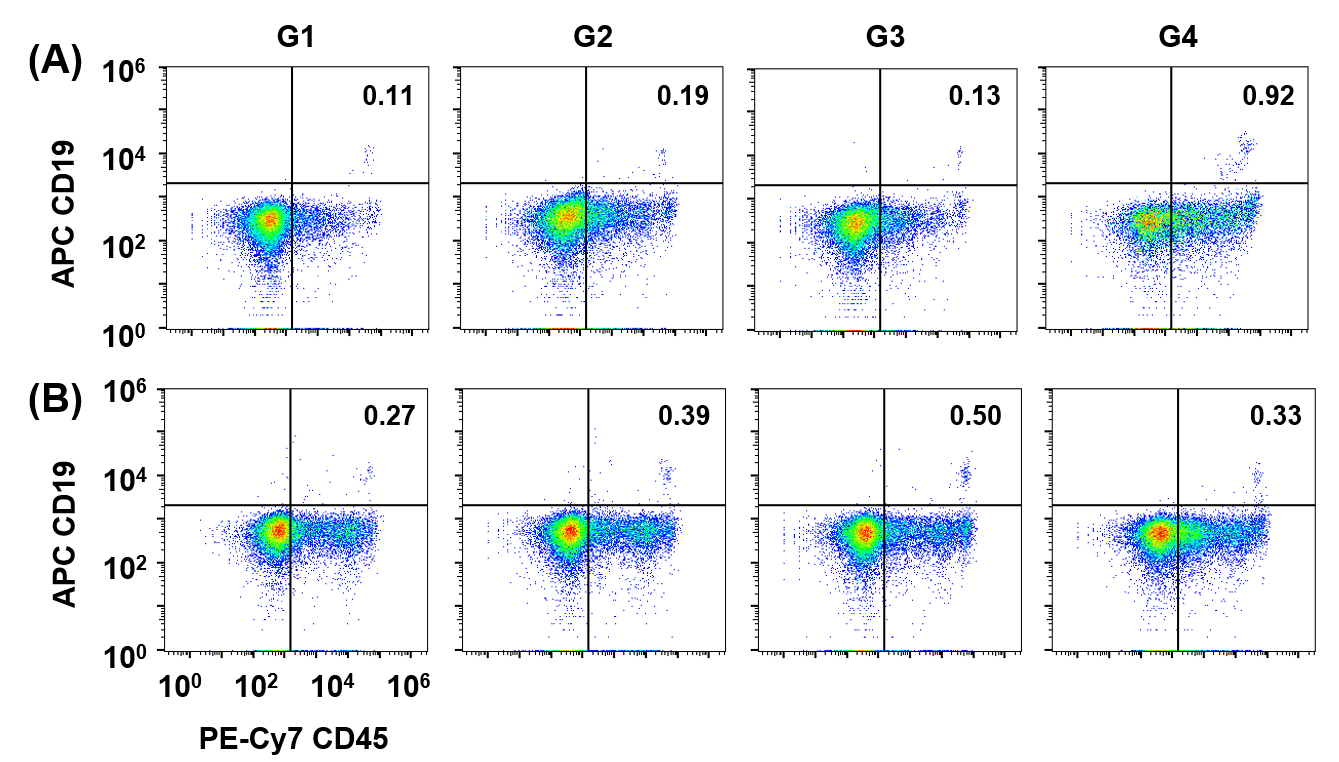


**Supplementary Figure 41.** Representative ﬂow cytometry dot plots showing the percentage of B cells (CD45^+^CD19^+^ cells) in tumors (cf. Fig. 8F). **(A)** CT26 xenograft. **(B)** 4T1 xenograft.


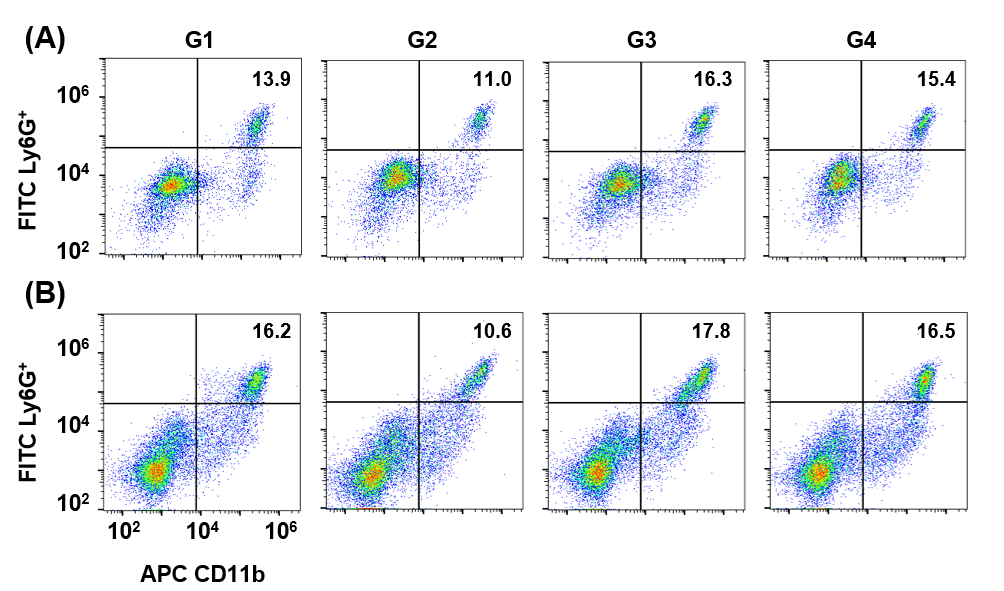


**Supplementary Figure 42.** Representative ﬂow cytometry dot plots showing the percentage of neutrophils (CD11b^+^Ly6G^+^ cells) in tumors (cf. Supplementary Figure 37B). **(A)** CT26 xenograft. **(B)** 4T1 xenograft.


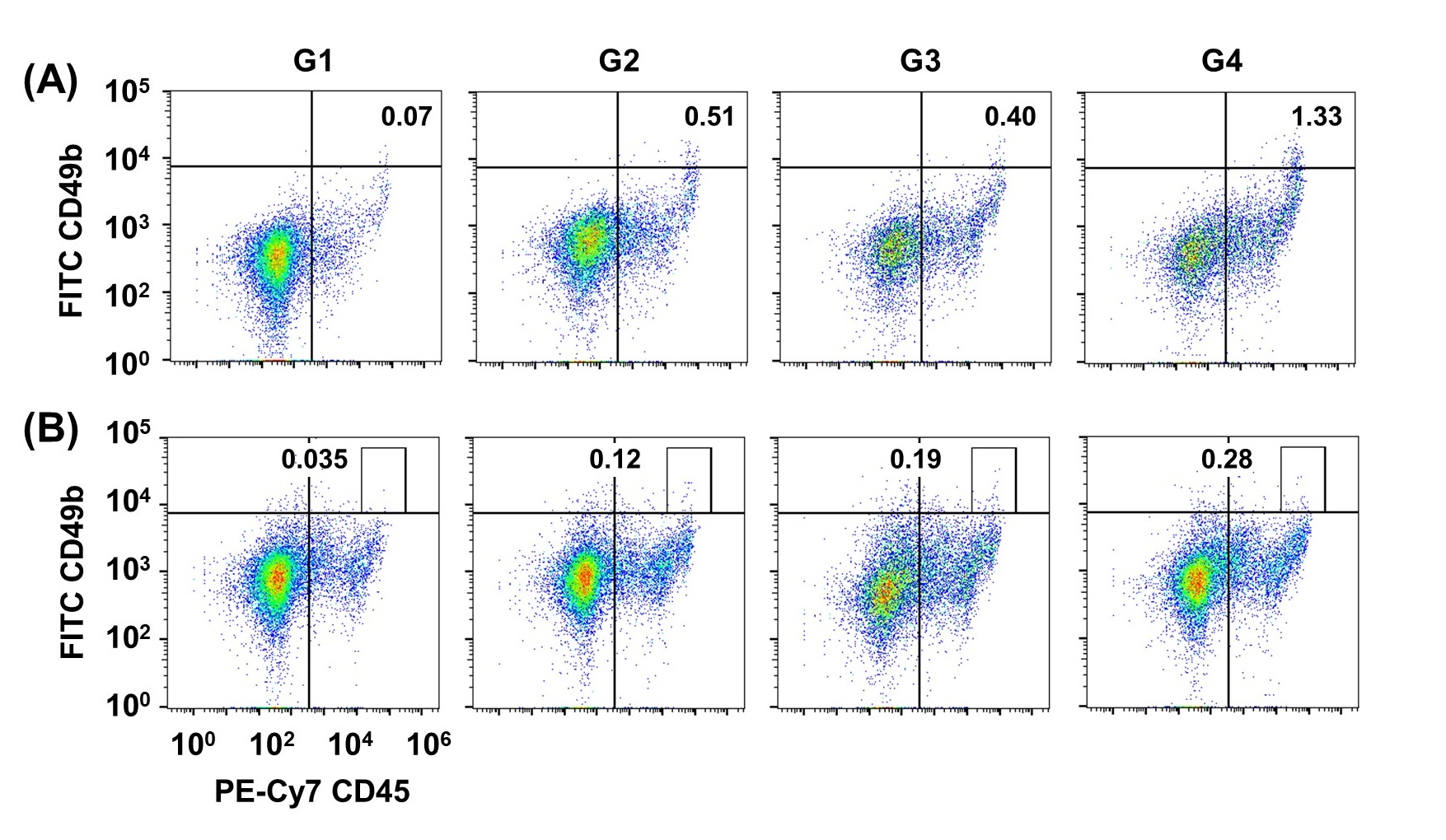


**Supplementary Figure 43.** Representative ﬂow cytometry dot plots showing the percentage of NK cells (CD45^+^CD49b^+^ cells) in tumors (cf. Fig. 8G). **(A)** CT26 xenograft. **(B)** 4T1 xenograft.


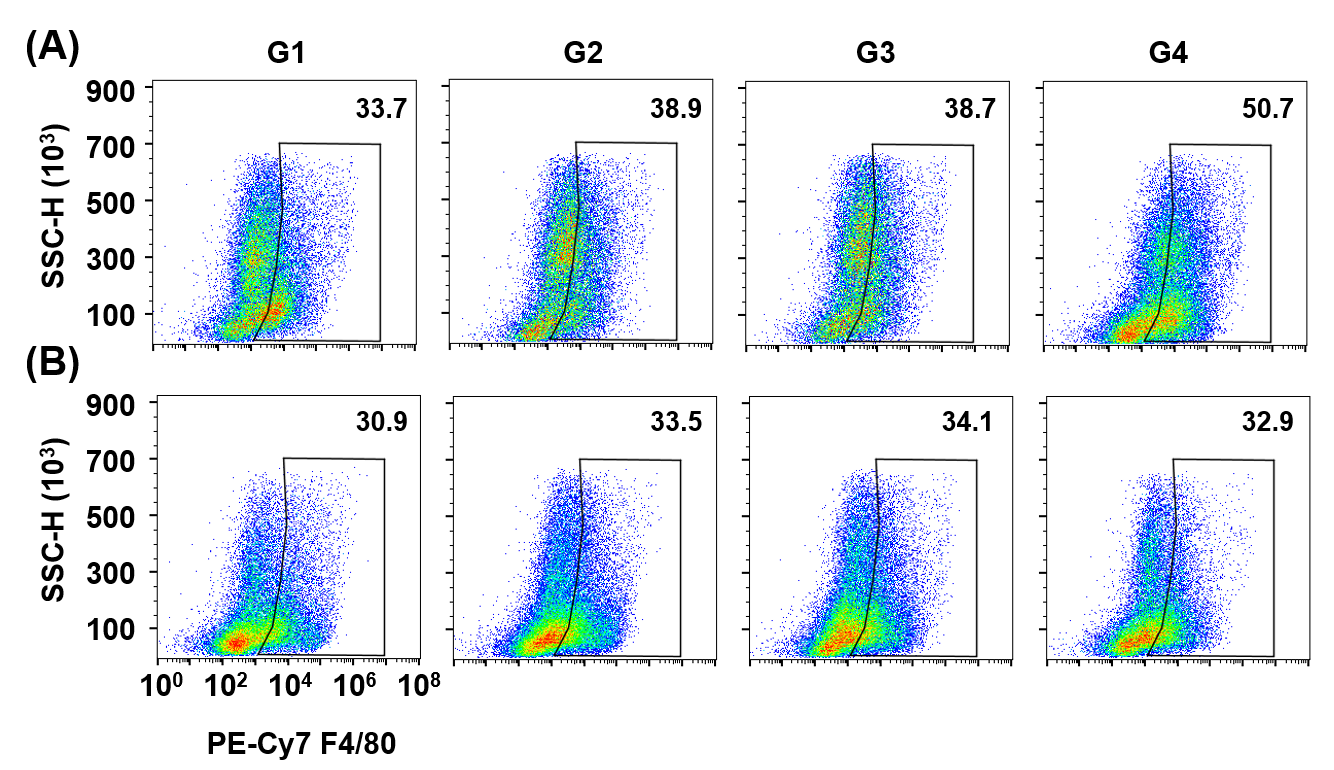


**Supplementary Figure 44.** Representative ﬂow cytometry dot plots showing the percentage of macrophages (F4/80^+^ cells) in tumors (cf. Fig. 8H). **(A)** CT26 xenograft. **(B)** 4T1 xenograft.


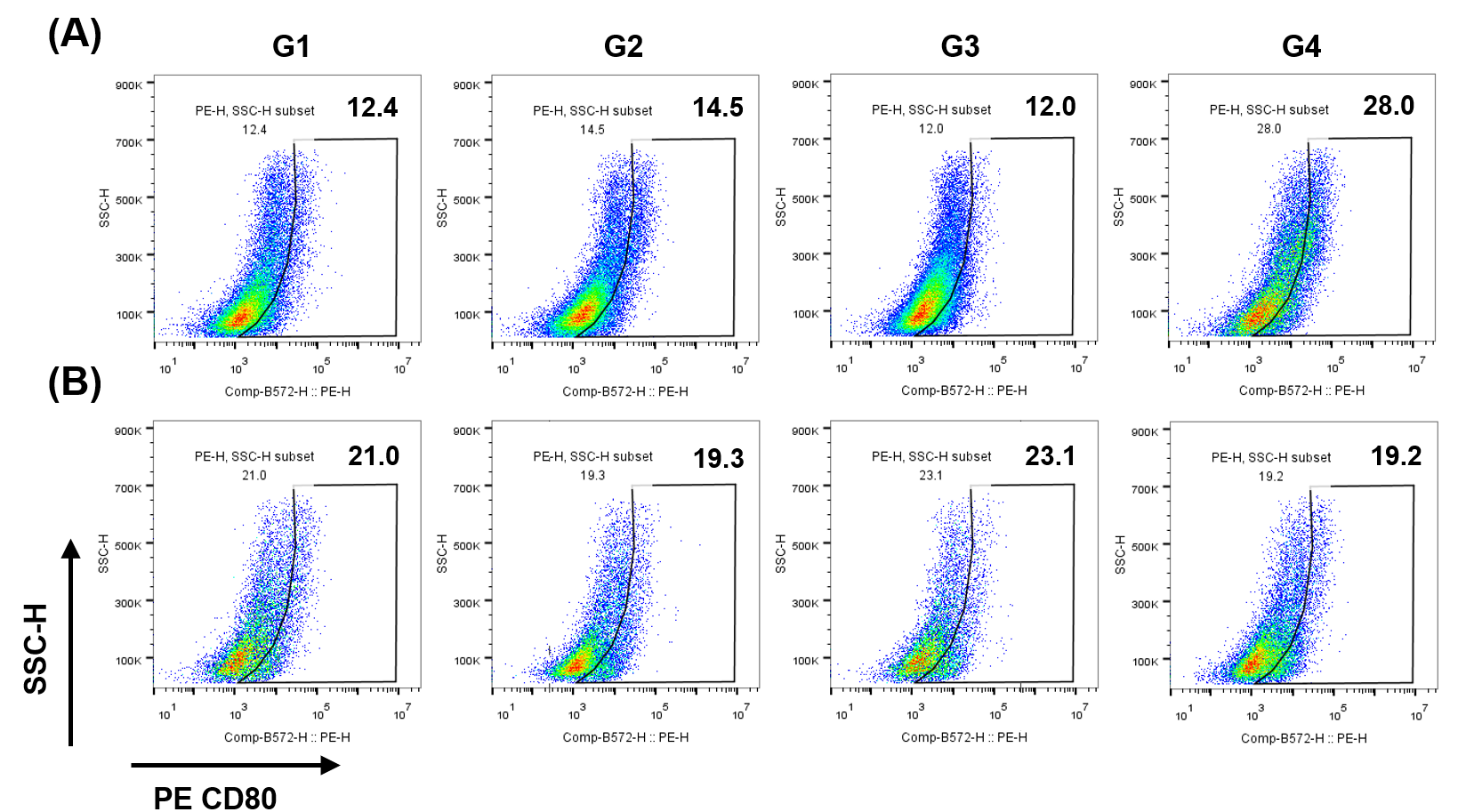


**Supplementary Figure 45.** Representative ﬂow cytometry dot plots showing the percentage of M1 macrophages (F4/80^+^CD80^+^ cells) in tumor-infiltrating macrophages (cf. Fig. 8I). **(A)** CT26 xenograft. **(B)** 4T1 xenograft.


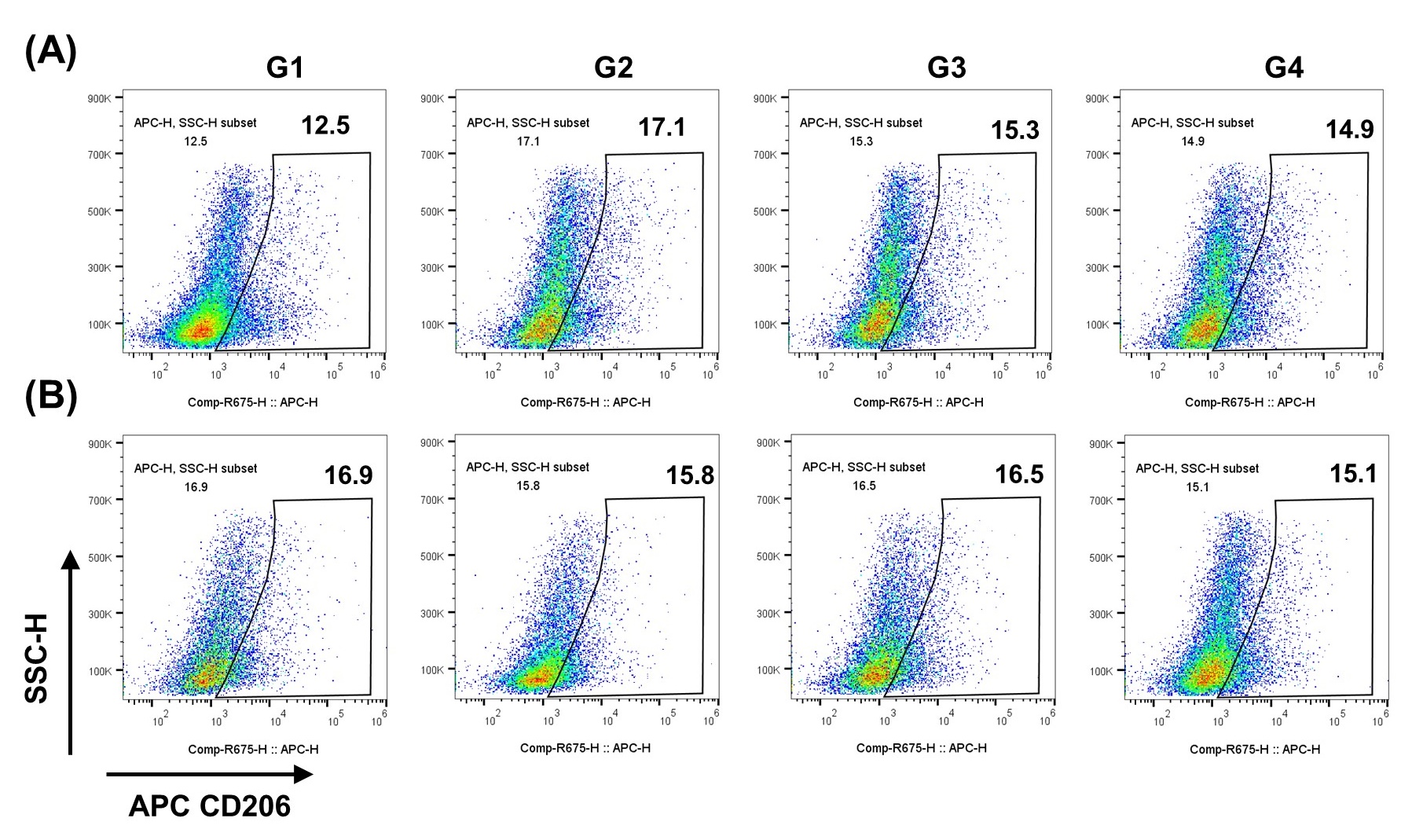


**Supplementary Figure 46.** Representative ﬂow cytometry dot plots showing the percentage of M2 macrophages (F4/80^+^CD206^+^ cells) in tumor-infiltrating macrophages (cf. Supplementary Figure 37C). **(A)** CT26 xenograft. **(B)** 4T1 xenograft.


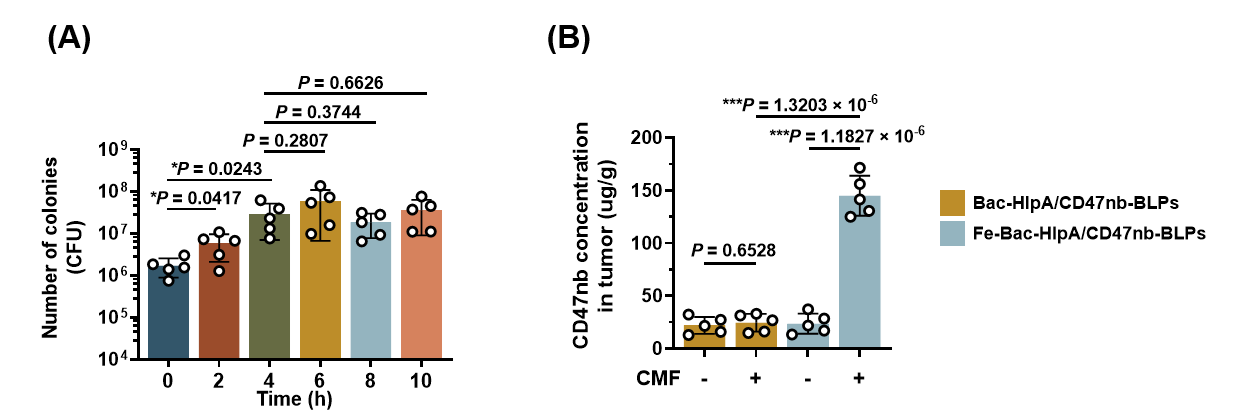


**Supplementary Figure 47**. **(A)** The intratumoral number of live bacteria after treating tumors with one-dimension CMF for different time (0-10 h, n = 5 tumors). Increased bacterial colonization in tumors was observed by controlling AMF-Bac motion to target to tumors using one-dimension CMF. BALB/c mice bearing subcutaneous CT-26-luc tumors in the right hind limb were *i.v.* injected with the Fe-Bac-HlpA/CD47nb-BLPs, and the NdFeB magnet was placed close to the tumor for different time (0-10 h). The tumors were collected and ground at 24 h after injection, and the suspension was serially diluted. The number of live bacteria in the tumor was measured by the spread plate method. **(B)** The intratumoral concentration of CD47nb examined by ELISA (n = 5 tumors). BALB/c mice bearing subcutaneous CT-26-luc tumors in the right hind limb were *i.v.* injected with the Bac-HlpA/CD47nb-BLPs or Fe-Bac-HlpA/CD47nb-BLPs, and the NdFeB magnet was placed close to the tumor for 4 h after injection. AMF treatment (310 kHz and 23.8 kA/m) was given for 80 min at 24 h later. Tumors were collected after another 6 h, and the grinding suspension was added with concentrated extraction buffer (pH 7.4). The mixture was maintained with constant agitation for 2 h at 4°C, and centrifuged for 20 min at 10,000 g. The CD47nb in the supernatant was quantified with His-tag Protein ELISA kit. The data are shown as the mean ± SD. Statistical analysis was performed by a two-tailed unpaired *t* test. ^*^, *P* < 0.05; ^**^, *P* < 0.01; ^***^, *P* < 0.001. Source data are provided as a Source Data file.


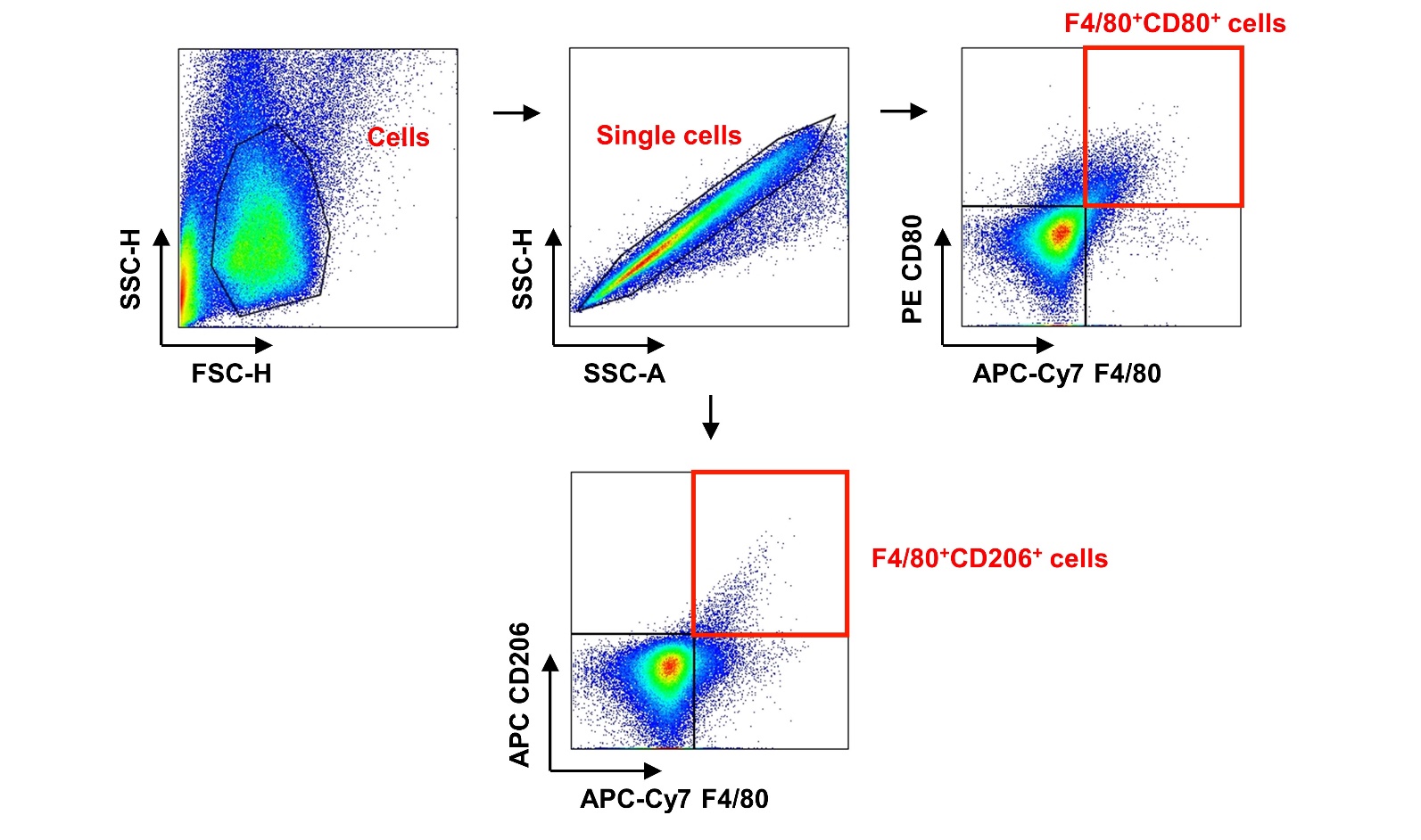


**Supplementary Figure 48.** Gating strategy for M1 and M2 macrophages (F4/80^+^CD80^+^ cells and F4/80^+^CD206^+^ cells) in tumors (cf. Fig. 6B, Supplementary Fig. 25A, and Supplementary Fig. 26).


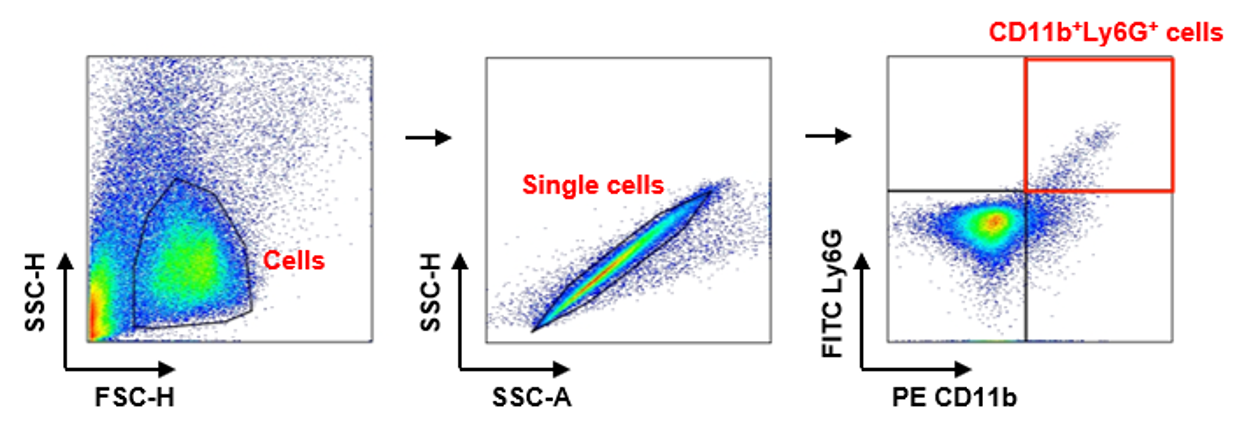


**Supplementary Figure 49.** Gating strategy for tumor-infiltrating neutrophils (CD11b^+^Ly6G^+^ cells) in tumors (cf. Fig. 6E, Supplementary Fig. 27).


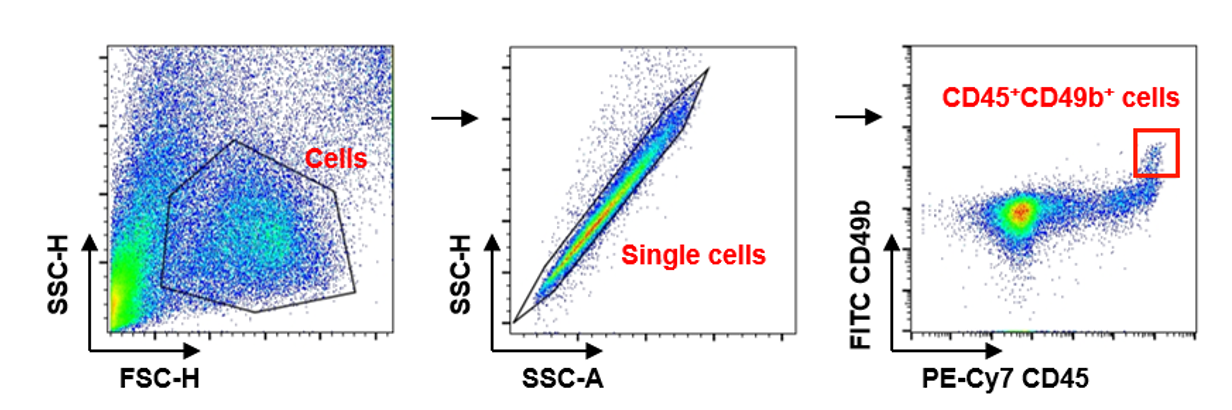


**Supplementary Figure 50.** Gating strategy for tumor-infiltrating NK cells (CD45^+^CD49b^+^ cells) in tumors (cf. Fig. 6F, Supplementary Fig. 28).


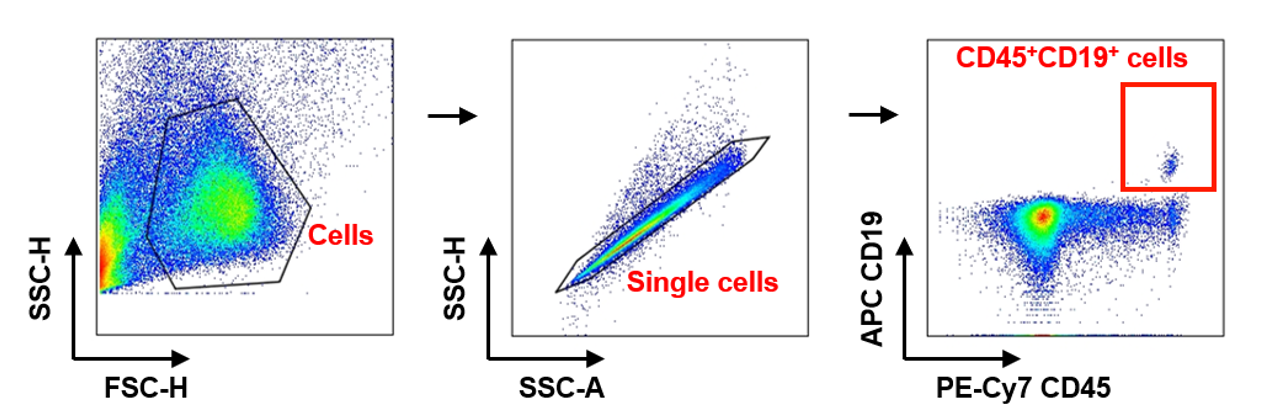


**Supplementary Figure 51.** Gating strategy for tumor-infiltrating B cells (CD45^+^CD19^+^ cells) in tumors (cf. Fig. 6I, Supplementary Fig. 32).


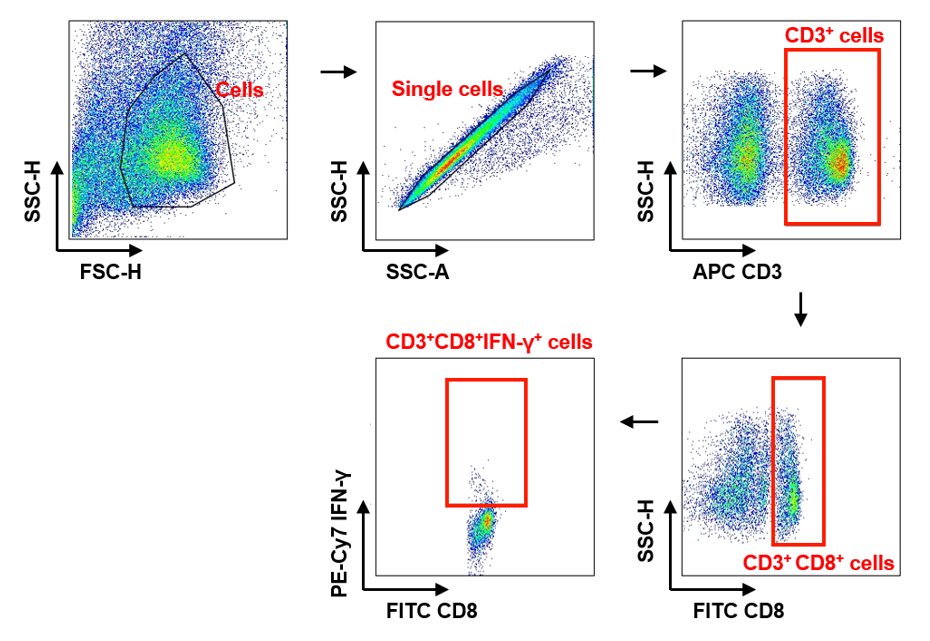


**Supplementary Figure 52.** Gating strategy for CD3^+^CD8^+^IFN-γ^+^ cells in splenocytes after re-stimulation with the CT26-specific antigen peptide (cf. Supplementary Fig. 25E, Supplementary Fig. 33).


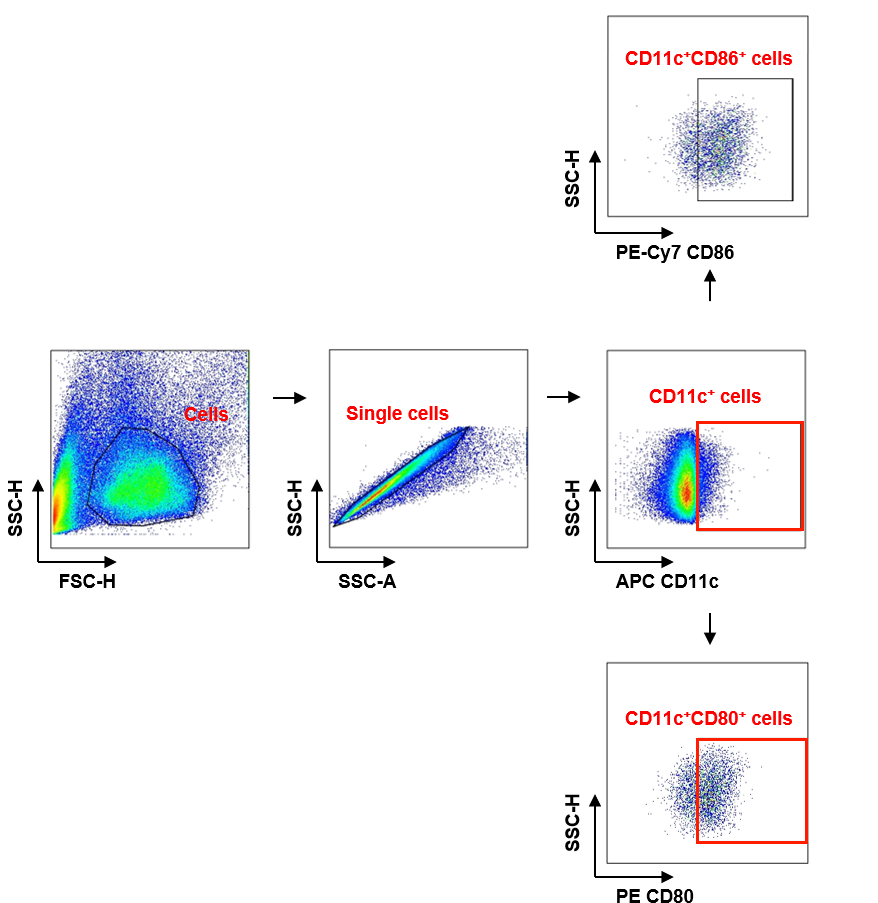


**Supplementary Figure 53.** Gating strategy for matured DC cells (CD11c^+^CD80^+^ and CD11c^+^CD86^+^ cells) in tumors (cf. Supplementary Fig. 25G-I, Supplementary Fig. 34-35).
