# Supplementary material for: Modular-designed engineered bacteria for precision tumor immunotherapy via spatiotemporal manipulation by magnetic field
Source: Nat Commun. 2023 Mar 23;14:1606. doi: 10.1038/s41467-023-37225-1 (PMC10036336; doi:10.1038/s41467-023-37225-1)
Supplement: Supplementary file 2 — Reporting Summary [file 41467_2023_37225_MOESM2_ESM.pdf]

nature portfolio

Corresponding author(s): Guangjun Nie, Xiao Zhao

Last updated by author(s): Mar 3, 2023

Reporting Summary

Nature Portfolio wishes to improve the reproducibility of the work that we publish. This form provides structure for consistency and transparency in reporting. For further information on Nature Portfolio policies, see our Editorial Policies and the Editorial Policy Checklist.

Statistics

For all statistical analyses, confirm that the following items are present in the figure legend, table legend, main text, or Methods section.

n/a	Confirmed	
☐	☒	The exact sample size (*n*) for each experimental group/condition, given as a discrete number and unit of measurement
☐	☒	A statement on whether measurements were taken from distinct samples or whether the same sample was measured repeatedly
☐	☒	The statistical test(s) used AND whether they are one- or two-sided<br>*Only common tests should be described solely by name; describe more complex techniques in the Methods section.*
☒	☐	A description of all covariates tested
☐	☒	A description of any assumptions or corrections, such as tests of normality and adjustment for multiple comparisons
☐	☒	A full description of the statistical parameters including central tendency (e.g. means) or other basic estimates (e.g. regression coefficient) AND variation (e.g. standard deviation) or associated estimates of uncertainty (e.g. confidence intervals)
☐	☒	For null hypothesis testing, the test statistic (e.g. *F*, *t*, *r*) with confidence intervals, effect sizes, degrees of freedom and *P* value noted<br>*Give P values as exact values whenever suitable.*
☒	☐	For Bayesian analysis, information on the choice of priors and Markov chain Monte Carlo settings
☒	☐	For hierarchical and complex designs, identification of the appropriate level for tests and full reporting of outcomes
☒	☐	Estimates of effect sizes (e.g. Cohen's *d*, Pearson's *r*), indicating how they were calculated

Our web collection on statistics for biologists contains articles on many of the points above.

Software and code

Policy information about availability of computer code

Data collection	NovoExpress (version number, 1.4.1.1901) and BD Accuri C6 Software (version number, 1.0.264.21) were used to collect data of flow cytometry. Living Image software (version number, 4.3.1.16427) was used for the bioluminescence assay. ZEN 2012 (version number, 1.1.13346.204) was used for the immunofluorescence detection.
Data analysis	ImageJ (version number, 1.8.0) was used to analyze the data of immunofluorescence. FlowJo (version number, 10.0.0.0), BD Accuri C6 Software (version number, 1.0.264.21) and NovoExpress (version number, 1.4.1.1901) were used to analyze the data of flow cytometry. GraphPad Prism (version number, 8.3.0.538) and IBM SPSS Statistics (version number, 19.0) were used for the statistical analysis.

For manuscripts utilizing custom algorithms or software that are central to the research but not yet described in published literature, software must be made available to editors and reviewers. We strongly encourage code deposition in a community repository (e.g. GitHub). See the Nature Portfolio guidelines for submitting code & software for further information.

Data

Policy information about availability of data

All manuscripts must include a data availability statement. This statement should provide the following information, where applicable:

- Accession codes, unique identifiers, or web links for publicly available datasets
- A description of any restrictions on data availability
- For clinical datasets or third party data, please ensure that the statement adheres to our policy

The authors declare that all the data supporting the results in this study are available within the Article, Supplementary Information or Source Data file. The gating

strategy for flow cytometry experiments can be found in Supplementary Fig. 48-53. Source data are provided with this paper.

Human research participants

Policy information about studies involving human research participants and Sex and Gender in Research.

Reporting on sex and gender	Not applicable.
Population characteristics	Not applicable.
Recruitment	Not applicable.
Ethics oversight	Not applicable.

Note that full information on the approval of the study protocol must also be provided in the manuscript.

Field-specific reporting

Please select the one below that is the best fit for your research. If you are not sure, read the appropriate sections before making your selection.

☒ Life sciences ☐ Behavioural & social sciences ☐ Ecological, evolutionary & environmental sciences

For a reference copy of the document with all sections, see nature.com/documents/nr-reporting-summary-flat.pdf

Life sciences study design

All studies must disclose on these points even when the disclosure is negative.

Sample size	No statistical methods were used to determine sample size. Sample sizes were determined on the basis of the previous experimental experience (Refs: Nature Communications 2021, 12: 2041; Science Translational Medicine 2021, 13: eabc2816; Nature Biomedical Engineering 2017, 1 : 667-679; Advanced Materials 2021 33: e2006007). In vitro experiments were performed with at least 3 biologically independent samples. All in vivo experiments were performed with at least 3 independent animals. Sample sizes were sufficient to perform statistical analyses.
Data exclusions	No samples were excluded.
Replication	All attempts at replication were successful. Experimental repeat numbers are also reported in Figure Legends.
Randomization	All samples/organisms were numbered and randomly grouped by random number table method.
Blinding	All experimental procedures and quantification of results, including injections, isolation of the tumors or organs, tissue histological analysis and flow cytometry, were done by two independent researchers. Meanwhile, all researchers were blinded to group allocation.

Reporting for specific materials, systems and methods

We require information from authors about some types of materials, experimental systems and methods used in many studies. Here, indicate whether each material, system or method listed is relevant to your study. If you are not sure if a list item applies to your research, read the appropriate section before selecting a response.

Materials & experimental systems

n/a	Involved in the study	
☐	☒	Antibodies
☐	☒	Eukaryotic cell lines
☒	☐	Palaeontology and archaeology
☐	☒	Animals and other organisms
☒	☐	Clinical data
☒	☐	Dual use research of concern

Methods

n/a	Involved in the study	
☒	☐	ChIP-seq
☐	☒	Flow cytometry
☒	☐	MRI-based neuroimaging

Antibodies

Antibodies used	APC-anti-mouse CD3 (catalog No. 100235, clone name: 17A2, 1:100), PE-anti-mouse CD4 (catalog No. 100408, clone name: GK1.5, 1:100), FITC-anti-mouse CD8α (catalog No. 100705, clone name: 53-6.7, 1:100), PE/Cy7-anti-mouse IFN-γ (catalog No. 505826, clone name: XMG1.2, 1:100), PE-Cy7-anti-mouse CD45 (catalog No. 103114, clone name: 30-F11, 1: 100), FITC-anti-mouse CD49b (catalog

No. 103504, clone name: HMα2, 1: 100), PE-anti-mouse CD11b (catalog No. 101208, clone name: M1/70, 1: 100), APC-anti-mouse GR1 (catalog No. 108412, clone name: RB6-8C5, 1: 100), FITC-anti-mouse Ly-6G (catalog No. 127606, clone name: 1A8, 1: 100), APC-Cy7-anti-mouse F4/80 (catalog No. 123118, clone name: BM8, 1: 100), PE-anti-mouse CD80 (catalog No. 104708, clone name: 16-10A1, 1: 100), PE/Cy7-anti-mouse CD86 (catalog No. 105014, clone name: GL-1, 1: 100), APC-anti-mouse CD19 (catalog No. 152409, clone name: 1D3/CD19, 1: 100), FITC anti-mouse CD172a (SIRPα) antibody (catalog No. 144005, clone name: P84, 1: 100), APC anti-mouse/human CD11b (catalog No. 101211, clone name: M1/70, 1: 100) were purchased from BioLegend (USA). APC-anti-mouse CD206 (catalog No. E-AB-F1135UE, clone name: C068C2, 1: 100) was purchased from Elabscience (China).

The anti-mouse CD47 antibody (catalog No. BE0270, clone name: MIAP301, 1: 1000 for in vitro use, 20 mg/kg for in vivo use), anti-mouse CD8 antibody (catalog No. BE0061, clone name: 2.43, 15 mg/kg for in vivo use), anti-mouse IFNAR1 antibody (catalog No. BE0241, clone name: MAR1-5A3, 15 mg/kg for in vivo use), rat anti-mouse CSF1 neutralizing antibody (catalog No. BE0204, Clone: 5A1, 100 μg/mouse for in vivo use), rat IgG1 isotype control (catalog No. BE0088, Clone: HRPN, 100 μg/mouse for in vivo use), and anti-FcR antibody (catalog No. BE0307, clone name: 2.4G2, 1.0 μg per 1000000 cells in 100 μL volume) were purchased from BioXcell (USA).

Anti-E. coli O + E. coli K antibody (Catalog No. ab31499, 1:100), Alexa Fluor® 647-conjuaged anti-6X His tag® antibody (Catalog No. ab237337, clone name: EPR20547, 1: 100), anti-syndecan-1 antibody (Catalog No. ab128936, clone: EPR6454, 1: 1000) were purchased from Abcam (UK).

Rabbit anti-Myc tag antibody (catalog NO. bs-0842R, polyclonal antibody, 1: 1000) were purchased from Bioss (USA).

Validation

All primary antibodies were purchased from the supplier as noted above and used without additional validation. The validation of all the antibodies could be found from manufacturers online:
https://www.biolegend.com/en-us/products/apc-anti-mouse-cd3-antibody-8055
https://www.biolegend.com/en-us/products/pe-anti-mouse-cd4-antibody-250
https://www.biolegend.com/en-us/products/fitc-anti-mouse-cd8a-antibody-153
https://www.biolegend.com/en-us/products/pe-cyanine7-anti-mouse-ifn-gamma-antibody-5865
https://www.biolegend.com/en-us/products/pe-cyanine7-anti-mouse-cd45-antibody-1903
https://www.biolegend.com/en-us/products/fitc-anti-mouse-cd49b-antibody-297
https://www.biolegend.com/en-us/products/pe-anti-mouse-human-cd11b-antibody-349
https://www.biolegend.com/en-us/products/apc-anti-mouse-ly-6g-ly-6c-gr-1-antibody-456
https://www.biolegend.com/en-us/products/fitc-anti-mouse-ly-6g-antibody-4775
https://www.biolegend.com/en-us/products/apc-cyanine7-anti-mouse-f4-80-antibody-4072
https://www.biolegend.com/en-us/products/pe-anti-mouse-cd80-antibody-43
https://www.biolegend.com/en-us/products/pe-cyanine7-anti-mouse-cd86-antibody-3046
https://www.biolegend.com/en-us/products/apc-anti-mouse-cd19-antibody-13680
https://www.biolegend.com/en-us/products/fitc-anti-mouse-cd172a-sirpalpha-antibody-7829
https://www.biolegend.com/en-us/products/apc-anti-mouse-human-cd11b-antibody-345
https://www.elabscience.cn/p-apc_anti_mouse_cd206_antibody_c068c2_-192468.html

https://bxcell.com/product/invivomab-mouse-cd47-iap/
https://bioxcell.com/invivomab-anti-mouse-cd8a-be0061#tab_references
https://bioxcell.com/invivomab-anti-mouse-ifnar-1-be0241#tab_references
https://bioxcell.com/invivomab-anti-mouse-csf1-be0204
https://bioxcell.com/invivomab-rat-igg1-isotype-control-anti-horseradish-peroxidase-be0088
https://bioxcell.com/invivomab-anti-mouse-cd16-cd32

https://www.abcam.cn/e-coli-o--e-coli-k-antibody-ab31499.html
https://www.abcam.cn/alexa-fluor-647-6x-his-tag-antibody-epr20547-ab237337.html
https://www.abcam.cn/syndecan-1-antibody-epr6454-ab128936.html

http://www.bioss.com.cn/prolook_03.asp?id=AF08169606000935&pro37=1

Eukaryotic cell lines

Policy information about cell lines and Sex and Gender in Research

Cell line source(s)

MC-38, 4T1, HCT-116, and MCF-7 cells were originally obtained from the American Type Culture Collection (Manassas, VA, USA). CT-26-luci cells were constructed from CT-26 that was originally purchased from American Type Culture Collection (Manassas, VA, USA) through stable transfection of the luciferase gene (Gene ID: 116160065).

Authentication

CT-26, MC-38, 4T1, HCT-116, and MCF-7 cells were not performed with authentication.

Mycoplasma contamination

CT-26, MC-38, 4T1, HCT-116, and MCF-7 cell lines were carried out with mycoplasma detection and were negative for mycoplasma contamination.

Commonly misidentified lines
(See ICLAC register)

CT-26, MC-38, 4T1, HCT-116, and MCF-7 cell lines are not listed in the database.

Animals and other research organisms

Policy information about studies involving animals; ARRIVE guidelines recommended for reporting animal research, and Sex and Gender in Research

Laboratory animals	All animal studies were performed in accordance with ARRIVE guidelines. Female, 6-7-week-old BALB/c mice were obtained from Vital River Laboratory Animal Technology Co. Ltd (China). All animal experiments were approved by the Institutional Animal Care and Use Committee of the National Center for Nanoscience and Technology. Mice were housed in a room with a temperature of 20-22 degrees Celsius and a humidity of 30-70%. Feed and water were available ad libitum. Artificial light was provided in a 12 h light/12 h dark cycle. This study complied with relevant ethical regulations for animal testing and research.
Wild animals	This study did not involve wild animals.
Reporting on sex	All animal experiments were conducted on female mice.
Field-collected samples	The study did not involve samples collected from the field.
Ethics oversight	All animal studies were approved by the Institutional Animal Care and Use Committee of National Center for Nanoscience and Technology.

Note that full information on the approval of the study protocol must also be provided in the manuscript.

Flow Cytometry

Plots

Confirm that:

- [x] The axis labels state the marker and fluorochrome used (e.g. CD4-FITC).
- [x] The axis scales are clearly visible. Include numbers along axes only for bottom left plot of group (a 'group' is an analysis of identical markers).
- [x] All plots are contour plots with outliers or pseudocolor plots.
- [x] A numerical value for number of cells or percentage (with statistics) is provided.

Methodology

Sample preparation	After in vivo therapy, tumor tissues were excised and digested with 1 mg/ml collagenase IV (ThermoFisher, USA) and 100 mg/ml DNase I (ThermoFisher, USA). Digestion was performed at 37°C with 5% CO2 for 30 min. A 70-µm cell filter was used to prepare single cell suspensions, and cells were washed with PBS containing 2% FBS. For flow cytometric sorting and analysis, the digested cells were blocked with anti-FcR (Clone: 2.4G2, BioXcell) and then stained with fluorescence-labelled antibodies against protein markers of different immune cells for 30 min. The dilution of antibodies was according to the manufacturer’s instructions. After washing with 1640 medium containing 2% FBS, the cells were analyzed on a NovocyteTM Flow Cytometer (ACEA) or sorted on a FACSAria II Cell Sorter (BD).<br>On day 9 of the in vivo therapy protocol, splenocytes were isolated from mouse spleens for intracellular IFN-γ flow cytometry analysis. Splenocytes were co-cultured with a CT-26-specific antigen peptide (AH1 [6-14], sequence: SPSYVYHQF) overnight. Splenocytes treated with ionomycin (Abmole, USA) were used as positive controls. 5 h before collection, monensin (MCE, USA) was added to the splenocytes. The cells were collected for labeling with anti-CD3 and anti-CD8 antibodies, followed by fixation and permeation in fixation and permeation buffer (BioLegend, USA). The cells were further stained with an anti-IFN-γ antibody before analysis on a flow cytometer.
Instrument	BD Accuri C6 (BD Biosciences, USA) and Agilent NovoCyte (USA) were used to analyze samples.
Software	BD Accuri C6 Software (version number, 1.0.264.21), FlowJo (version number, 10.0.0.0) and NovoExpress (version number, 1.4.1.1901)
Cell population abundance	Over 10000 cells were analyzed for fluorescent intensity in the defined gate.
Gating strategy	A gate is drawn around the cells. Single cells are determined with the area and the height of the side scatter (SSC). The analysis was carried out in this gate.

- [x] Tick this box to confirm that a figure exemplifying the gating strategy is provided in the Supplementary Information.
